# Combination of ipratropium bromide and salbutamol in children and adolescents with asthma: A meta-analysis

**DOI:** 10.1371/journal.pone.0237620

**Published:** 2021-02-23

**Authors:** Hongzhen Xu, Lin Tong, Peng Gao, Yan Hu, Huijuan Wang, Zhimin Chen, Luo Fang

**Affiliations:** 1 Department of Pulmonology, The Children’s Hospital, Zhejiang University School of Medicine, National Clinical Research Center for Child Health, Hangzhou, Zhejiang, China; 2 Department of Pharmacy, The Children’s Hospital, Zhejiang University School of Medicine, National Clinical Research Center for Child Health, Hangzhou, Zhejiang, China; All India Institute of Medical Sciences, Bhopal, INDIA

## Abstract

**Background:**

A combination of ipratropium bromide (IB) and salbutamol is commonly used to treat asthma in children and adolescents; however, there has been a lack of consistency in its usage in clinical practice.

**Objective:**

To evaluate the efficacy and safety of IB + salbutamol in the treatment of asthma in children and adolescents.

**Methods:**

The MEDLINE, Embase, and Cochrane Library as well as other Chinese biomedical databases (including China Biological Medicine Database, Chinese National Knowledge Infrastructure, Chongqing VIP, and Wanfang Chinese language bibliographic database) were systematically searched from the earliest record date to September 2020 for randomized controlled trials in children and adolescents (≤18 years) with asthma who received IB + salbutamol or salbutamol alone. The primary outcomes included hospital admission and adverse events. A random effects model with a 95% confidence interval (CI) was used. Subgroup analysis was performed according to age, severity of asthma, and co-interventions with other asthma controllers. This study was registered with PROSPERO.

**Results:**

Of the 1061 studies that were identified, 55 met the inclusion criteria and involved 6396 participants. IB + salbutamol significantly reduced the risk of hospital admission compared with salbutamol alone (risk ratio [RR] 0.79; 95% CI 0.66–0.95; p = 0.01; I^2^ = 40%). Subgroup analysis only showed significant difference in the risk of hospital admission in participants with severe asthma exacerbation (RR 0.73; 95% CI 0.60–0.88; p = 0.0009; I^2^ = 4%) and moderate-to-severe exacerbation (RR 0.69; 95% CI 0.50–0.96; p = 0.03; I^2^ = 3%). There were no significant differences in the risk of adverse events between IB + salbutamol group and salbutamol alone group (RR 1.77; 95% CI 0.63–4.98).

**Conclusion:**

IB + salbutamol may be more effective than salbutamol alone for the treatment of asthma in children and adolescents, especially in those with severe and moderate to severe asthma exacerbation. The very low to high quality of evidence indicated that future well-designed double-blind RCTs with large sample are needed for research on evaluating the effectiveness of IB + salbutamol treatment for asthma in children and adolescents.

## Introduction

Asthma is the most common chronic disease among children and is estimated to affect 300 million individuals worldwide [1]. In China, asthma affects 3% of children ≤14 years of age and the prevalence of childhood asthma has increased by 50% over the past 10 years [2]. Asthma-related hospitalization can negatively affect the quality of life of children and their caregivers. Additionally, health care expenditures for asthma-related conditions impose considerable economic burden on society [3,4].

Almost all available guidelines recommend that the repeated administration of inhaled short-acting β_2_-agonists (SABAs, up to 4–10 puffs every 20 minutes for the first hour) is an effective and efficient way to achieve rapid reversal of airflow limitation in patients with mild-to-moderate asthma exacerbation [2,5]. In the latest guideline [6], SABA-only treatment is no longer recommended for asthma in adults or adolescents due to its risk of asthma-related death and urgent asthma-related healthcare. Currently, several available guidelines [6–9] have recommended the addition of ipratropium bromide (IB), a short-acting muscarinic acetylcholine receptor antagonist, to SABAs as an optional treatment for children and adolescents with acute asthma exacerbation. Although IB does not seem to be very efficient in controlling asthma, several studies have demonstrated that a combination of IB and SABAs is associated with fewer hospitalizations and greater improvement in peak expiratory flow (PEF) and forced expiratory volume in one second (FEV_1_) compared with SABA alone in children and adolescents with moderate-to-severe asthma exacerbation [10–15]. The addition of IB to SABA has been recommended in the first hour of treatment for children with moderate-severe exacerbations [6]. However, these recommendations lack uniformity with respect to the optimal age, severity of asthma, and co-intervention with other asthma controllers for such therapy.

The most recent systematic review comparing IB + SABA and SABA alone for the treatment of acute asthma in children and adolescents was published in 2013 and reported a combined treatment benefit as evidenced by a decrease in the risk of hospital admission and improved lung function and clinical scores [14]. However, the review found no effect of age and co-intervention (such as steroid or standard care) on the hospital admission rate to treatment.

Since this last publication, there have been numerous studies published, and thus, this systematic review and meta-analysis was performed to update the evidence comparing salbutamol alone with IB + salbutamol for identifying the impact of the combination treatment in children and adolescents with asthma.

## Methods

### Registration

A prior protocol was developed and registered with PROSPERO (registration number: CRD42020159999). This review was informed by and reported using the Preferred Reporting Items for Systematic Reviews and Meta-Analyses guidelines [16–18] (S1 Appendix).

### Search strategy

An electronic search for studies published up to September 2020 using the MeSH descriptor “asthma,” “respiratory sounds”, “bronchial spasm”, “bronchoconstriction”, “bronchial hyperreactivity”, “respiratory hypersensitivity”, “ipratropium”, “albuterol”, “salbutamol”, “child”, “preschool”, and “pediatrics” were conducted using the following databases: MEDLINE, Embase, Cochrane Library, China Biological Medicine Database, Chinese National Knowledge Infrastructure, Chongqing VIP, and Wanfang Chinese language bibliographic database (S2 Appendix). The search strategy was independently developed by two investigators according to the following selection criteria. Any dispute was resolved by mutual consensus with a third investigator.

Eligible clinical studies were defined based on the following criteria: (1) randomized controlled trials (RCTs); (2) children and adolescents aged ≤18 years; we also included studies if the proportion of adult participants (age>18 years old) was less than 5%; (3) physician-diagnosed asthma by any appropriate diagnostic criteria, we also included children less than 1 year old who were diagnosed with wheezing; (4) comparing IB + salbutamol (either in a fixed dose or delivered separately) with salbutamol alone, regardless treatment duration, mode or frequency of administration, or dosage. We included studies if an additional treatment was equally applied to both IB + salbutamol group and salbutamol alone group, and it was only the intervention that was randomised. There was no limitation of language.

### Outcome measures

The primary outcomes that were measured were hospital admission (as defined by original studies) and any adverse events. Secondary outcomes included pulmonary function (including percentage change from baseline of predicted % forced expiratory volume in one second [FEV_1_]; percentage change from baseline of FEV_1_; and change from baseline in respiratory resistance); clinical score (as defined by original studies, including accessory muscle, asthma, cough, dyspnea, wheeze, wheezing sound, and daytime/night-time symptoms scores); oxygen saturation; need for extra medication (including systemic corticosteroids and repeated bronchodilators); specific adverse events (including dry mouth, nausea, vomiting, and tremor); and relapse rate (defined as a return visit within a certain time that was predefined by original studies).

### Data extraction and assessment of risk of bias

Data extraction was independently performed by two reviewers. Any ambiguities in the selection and extraction were resolved by discussion, with the assistance from a third party if necessary. Once extraction was completed, data were reviewed to identify duplicate studies and duplicate reporting of populations and only the longest follow-up studies were retained. The extracted data included general study characteristics (including first authors, publication years, study center, and sample size); demographic characteristics (including diagnosis, age, and settings); interventions and controls (including frequency and treatment duration); and outcome characteristics (including categories and definitions of outcome and follow-up).

The Cochrane Risk of Bias tool [18] was applied to assess the quality of the included RCTs, including sequence generation, allocation concealment, blinding of participants and personnel, blinding of outcome assessment, incomplete outcome data, selective outcome reporting, and other potential threats to validity. Studies were rated on each variable as low risk, high risk, or unclear risk of bias. If a study received “high risk” judgment in any one domain, it would be classified as “high risk of bias”. Two independent assessors conducted quality assessment, and any disagreement was settled by reaching a consensus or consulting a third researcher.

### Data analysis

Data were synthesized and analyzed using RevMan version 5.3 [16]. A random effects model was used to calculate pooled effect estimates comparing the outcomes between the intervention and control groups. For studies with data that could not be synthesized quantitatively for a meta-analysis, a descriptive synthesis would be performed. Dichotomous outcome results were expressed as risk ratio (RR) with 95% confidence intervals (CI). Continuous scales of measurement were expressed as a mean difference. Heterogeneity was calculated using the I^2^ statistic. For I^2^≥50%, the heterogeneity was classified as important and was interpreted according to study characteristics. Subgroup analysis was performed for primary outcomes based on the following characteristics: 1) age (<6, ≥6 years); 2) severity of disease (mild, moderate, severe, mild-to-moderate, or moderate-to-severe, as defined by original studies); and 3) presence of combined co-intervention (with steroid, without steroid, or with standard care, as defined by original studies) (for details, refer to S3 Appendix). For subgroup analysis, we used ‘mixed’ to indicate a study that contained two or more subgroups of the same characteristics but no split data was reported for each subgroup; we used ‘unclear’ to indicate a study where no relevant description of subgroup was stated. Sensitivity analysis was performed with the assumptions of lost binary data and risk of bias as described in protocol. Publication bias was analyzed using funnel plots and Egger’s test for outcomes when there were more than 10 studies.

The quality of evidence was assessed by the GRADEpro GDT according to risk of bias, inconsistency, indirectness, imprecision, and publication bias [19,20]. A summary of finding table was created for seven selected outcomes including hospital admission, predicted FEV_1_ in % at 60 mins and 120 mins after the combined ipratropium bromide and salbutamol, specific adverse event of dry mouth, nausea, tremor, and vomiting.

## Results

### Results of the search

**Fig 1:**
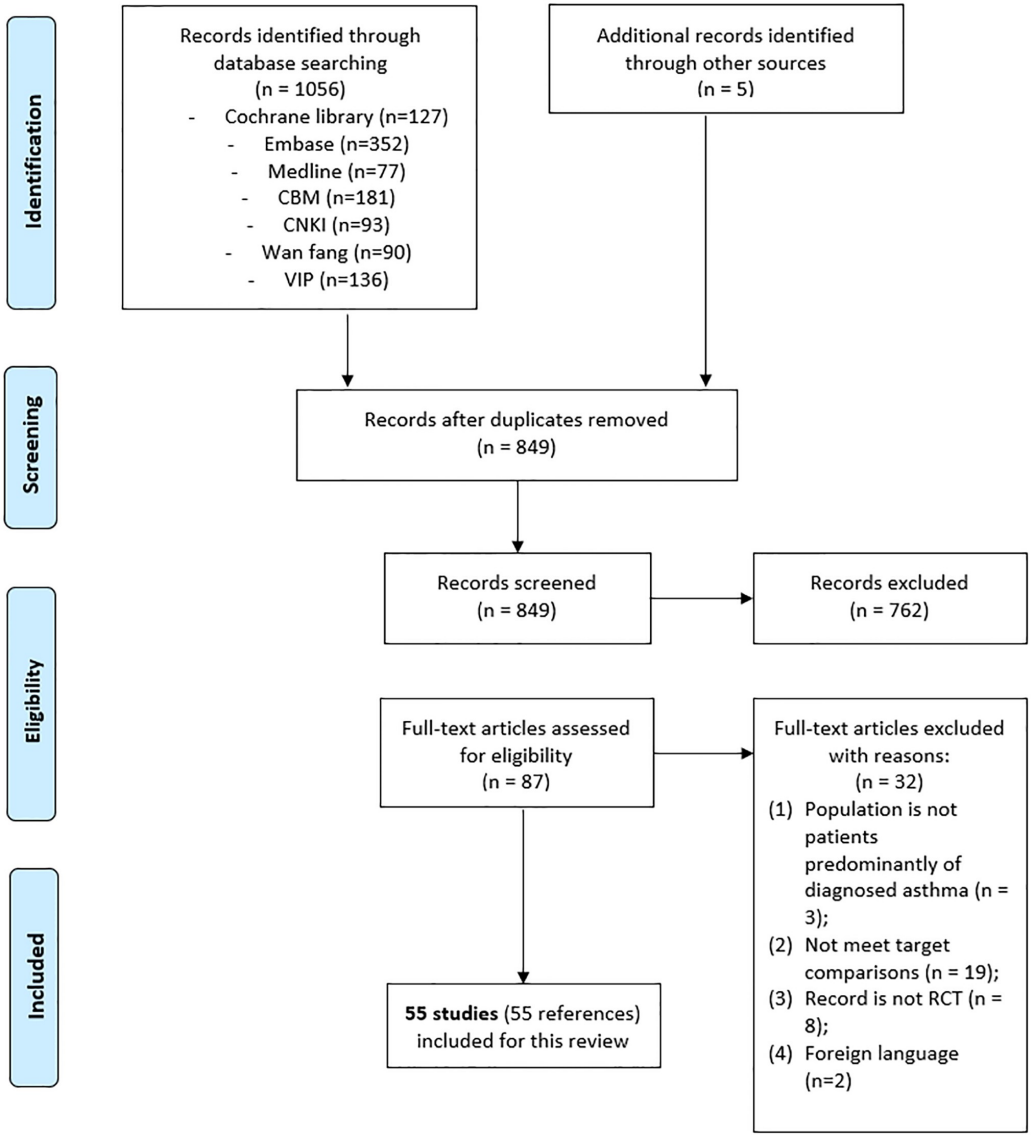
PRISMA flow diagram.

The initial electronic database search identified a total of 1056 references. Another five references were identified after checking the references listed in the relevant systematic reviews and included studies. After removing duplicate publications, 849 studies were included. After evaluating the titles and abstracts at first-level screening, 87 records were included. Assessment of the full text at second-level screening removed another 32 records. Finally, 55 RCTs were included. These RCTs involved 6396 participants and met the inclusion criteria for this review (Fig 1) (for full references, refer to S4 Appendix).

### Characteristics of included studies

The included 55 RCTs (53 published trials and 2 unpublished trials [21,22]) were from both developing and developed countries, including Australia, China, Canada, Chile, Greece, India, Mexico, Pakistan, Spain, Turkey, Thailand, the United Kingdom, and the United States. Of the 55 included RCTs, 58% (n = 32) reported a focus on acute asthma patients, 42% (n = 23) did not mention that they only focused on acute asthma patients. The age group varied across studies from 4 months to 18 years. Asthma severity varied from mild to severe on different scales across the trials. Co-interventions were administered in 31 studies, with 20 with steroids, and 11 with standard care (S3 Appendix). The frequency of IB + salbutamol treatment ranged from every 10 minutes to every 24 hours. Moreover, 23 studies reported that the treatment duration of IB + salbutamol was less than 120 minutes (median = 60 minutes), 18 studies reported that treatment duration ranged between 3 days and 40 weeks (median = 7 days); and 14 studies did not report treatment duration (S5 Appendix). All characteristic information was collected based on reported data from original studies.

### Risk of bias in the included studies

**Fig 2:**
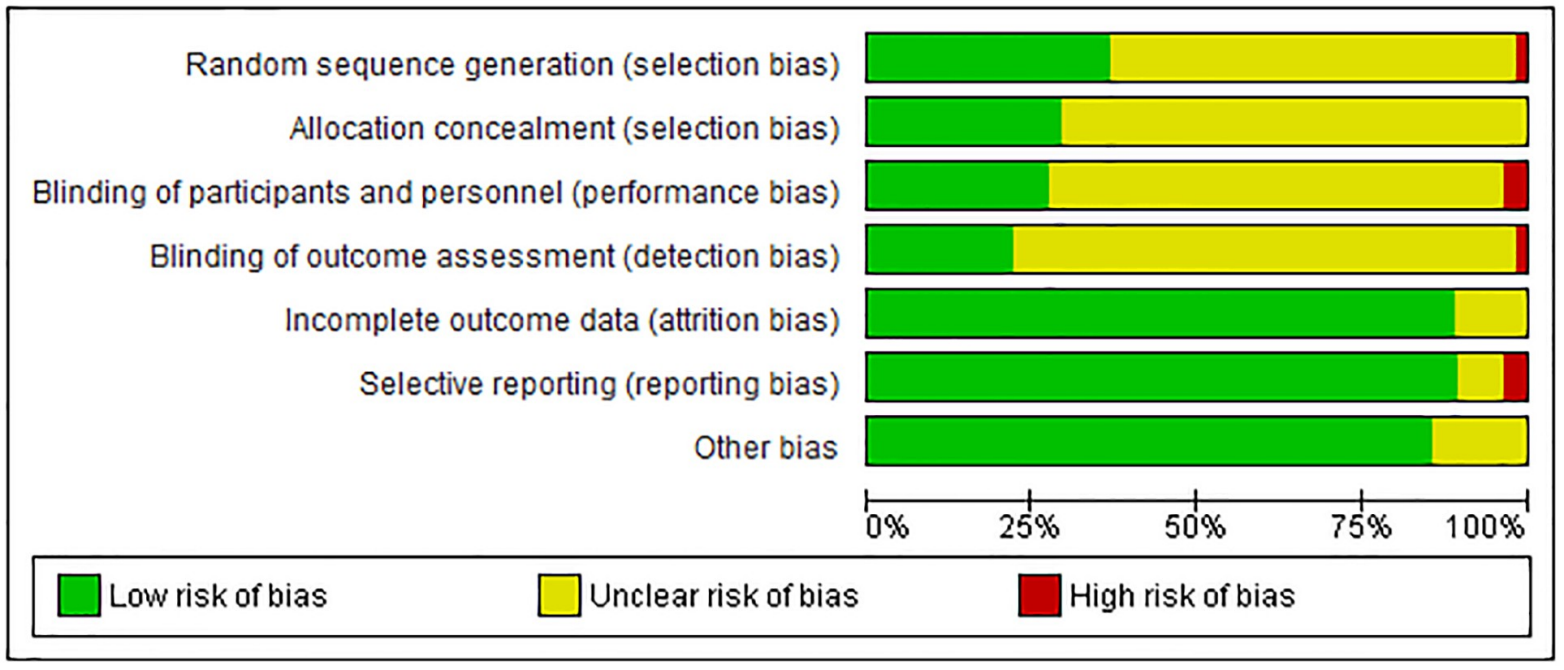
Summary of risk of bias of the included studies.

Quality analysis was performed on the basis of aforementioned methods and tools. Details of the risk of bias assessment are provided in Fig 2. Only one study was assessed as being at low risk of bias in all domains [10]. Five studies were considered to be at high risk of bias, one of which was due to random sequence generation [23], two due to blinding setting [17,24] and the other two due to selective reporting on predefined outcomes [12,25]. The remaining 49 studies were considered to be at unclear risk of bias (for details, refer to S6 Appendix).

### Primary outcome

**Fig 3:**
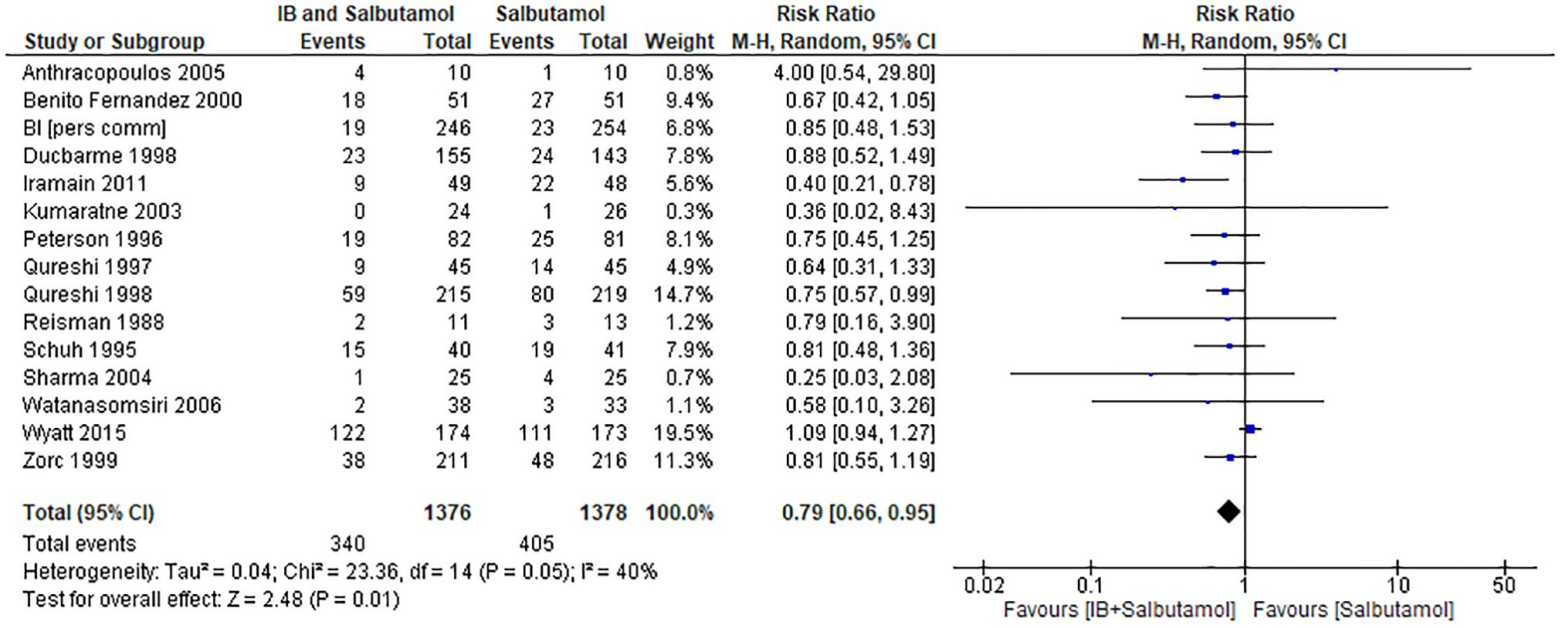
Forest plot for the meta-analysis of hospital admission.

Hospital admission was reported by 16 trials involving 2834 participants [10,11,13,21–24,26–34] with low quality of evidence (Table 1). The meta-analysis was conducted with 15 trials showed that compared with salbutamol alone, the IB + salbutamol group showed a significant reduction in the risk of hospital admission (RR 0.79; 95% CI 0.66–0.95; I^2^ = 40%; p = 0.01; Fig 3). One study [23] was not included in the meta-analysis because it reported a number of zero on hospital admission in intervention and comparison groups.

**Table 1:** Summary of secondary outcome—Pulmonary function at 60 and 120 minutes after treatment.

Study	Mean age, year (range)	Diagnosis	Patients	Intervention & Comparison	Significant key results
**Schuh 1995 Canada**	9.3 (5–17)	Acute asthma	121	IB: 250 μg/dose (group 1: 3 doses; group 2: 1 dose) with salbutamol: 0.15 mg/kg/dose vs. Albuterol: 0.15 mg/kg/dose; every 20 minutes for 60 minutes	IB+ salbutamol improved predict FEV_1_%: 24.72 vs. 13.1, MD 11.61, p<0.05 at 60 mins; predict FEV_1_%: 20.78 vs. 13, MD 7.781, p<0.05) at 120 mins.
**Peterson 1996**^a^ **Canada**	NR (5–12)	asthma	163	IB: 250 μg+Salbutamol: 3 mg vs. Salbutamol: 3 mg; both with Systemic corticosteroids: at discretion of treating physician; q45 minutes x 2 doses	Pulmonary function (predict FEV_1_%: 14.7 vs. 13.1) at 60 mins, not estimable.
**Qureshi 1997 United States**	12 (6–18)	asthma	90	IB: 500 μg+Albuterol: 0.15 mg/kg, maximum 5 mg vs. Albuterol: 0.15 mg/kg, maximum 5 mg. Both with prednisone or prednisolone: 2 mg/kg, maximum 60 mg; oral steroids together with the 2^nd^ dose of albuterol. Albuterol was used every 30 minutes for 3 doses, continued every 30 minutes as needed; IB was used after the first and the third dose of albuterol.	No significant differences in pulmonary function (predict FEV_1_%: 33.6 vs. 24.1) at 60 mins.
**Li 2000 China**	9.3 (NR)	Acute asthma	40	Salbutamol (0.5%): 2.5–5 mg (according to patients’ age) with IB (0.025%): 0.25–0.5 mg (according to patients’ age) vs. Salbutamol (0.5%): 2.5–5 mg (according to patients’ age); 5–10 minutes/time	IB+ salbutamol improved FEV_1_%: 33.29 vs. 23.62, MD 9.67, p<0.05 at 120 mins after the treatment. No significant differences in FEV_1_% at 60 mins after the treatment.
**Ducbarme 1998 Canada**	NR (3–17)	Acute asthma	275	IB: 1 mL (250μg)+Albutamol: 0.15 mg/kg (max. 5 mg) as an initial dose, then followed with IB every 30 minutes for a minimum of 1 hour vs. Albutamol: 0.15 mg/kg (max. 5 mg) as an initial dose followed with placebo every 30 minutes for a minimum of 1 hour. The use of corticosteroids or theophylline was decided by physicians.	No significant differences in pulmonary function (respiratory resistance) at 60 mins and 120 mins after the treatment.

NR, not reported.

^a^. Unpublished trial.

Regarding the subgroup analysis, there was a significant difference in hospital admission according to severity of illness (test for subgroup differences: χ^2^ = 12.79, df = 5, p = 0.03, I^2^ = 60.9%). Furthermore, administration of IB + salbutamol only showed a significant reduction in the hospital admission in participants with severe asthma exacerbation (RR 0.73; 95% CI 0.60–0.88; p = 0.0001; I^2^ = 4%; 1203 participants in nine studies [10,11,22,26,27,31–34] and moderate-to-severe asthma exacerbation (RR 0.69; 95% CI 0.50–0.96; p = 0.03; I^2^ = 3%; 629 participants in four studies [11,13,21,28]. There were no significant differences between IB + salbutamol and salbutamol alone in participants with mild asthma exacerbation (RR 1.43; 95% CI 0.42–4.79; p = 0.57; 117 participants in one study [34]), moderate asthma exacerbation (RR 1.04; 95% CI 0.89–1.22; p = 0.59; I^2^ = 2%; 736 participants in four studies [10,11,33,34]), and mild-to-moderate asthma exacerbation (RR 0.85; 95% CI 0.51–1.43; p = 0.54; 348 participants in two studies [28,29]). Additionally, there were no significant differences in the age subgroup (χ^2^ = 1.16, df = 2, p = 0.56, I^2^ = 0%) or the co-intervention subgroup (χ^2^ = 0.73, df = 3, p = 0.87, I^2^ = 0%) (for details, refer to S7 Appendix).

**Fig 4:**
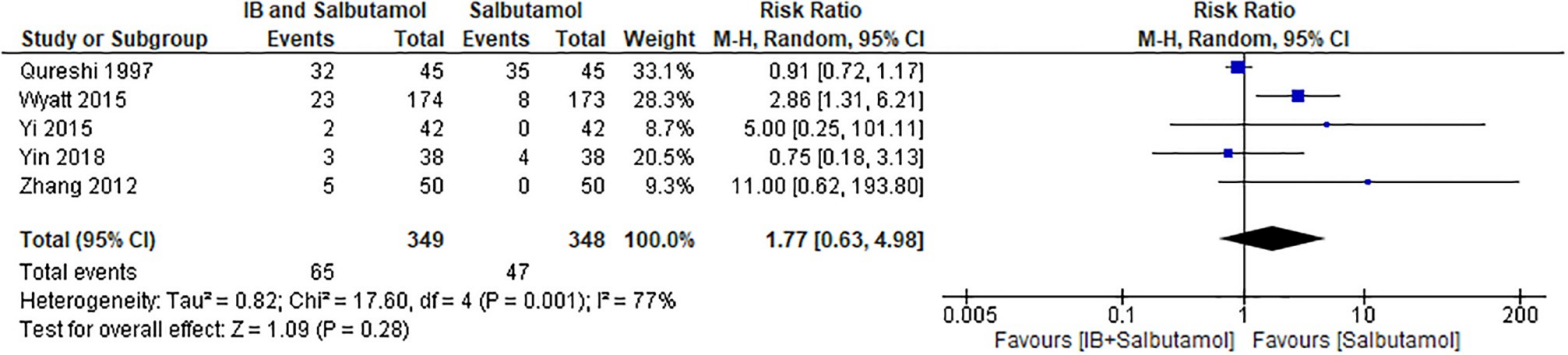
Forest plot for the meta-analysis of any adverse events.

Eight trials (with 1137 participants) reported the number of participants who had adverse events [30,33,35–39] with very low quality of evidence. Three trials reported a number of zero on adverse events in both intervention and comparison groups [35,36,40]. Based on reporting in the remaining five trials [30,33,37–39], 65 out of 349 participants had adverse events in the IB + salbutamol group, and 47 out of 348 participants had adverse events in the salbutamol alone group. The results of meta-analysis on these five trials (with 697 participants) [30,33,37–39] showed no significant differences on the incidence of adverse events between the compared groups (RR 1.77; 95% CI 0.63–4.98; p = 0.28; I^2^ = 77%, Fig 4). The substantial heterogeneity may be explained by the different treatment durations among the five studies in the meta-analysis. In two of the studies [30,33] patients were treated with IB + salbutamol for 60–90 minutes, whereas in the other three studies [37–39], patients were treated for 3–7 days. The differences in treatment durations may have led to clinical heterogeneity.

There were no significant differences in the subgroup analysis for the incidence of adverse events between IB + salbutamol and salbutamol alone in the severity subgroups (test for subgroup differences: χ^2^ = 1.49, df = 1, p = 0.22, I^2^ = 32.7%), age subgroups (test for subgroup differences: χ^2^ = 0.49, df = 1, p = 0.48, I^2^ = 0%), and co-intervention subgroups (test for subgroup differences: χ^2^ = 3.23, df = 2, p = 0.20, I^2^ = 38.1%). Only one study [33] including 347 participants with moderate asthma exacerbation reported a significant reduction in the number of adverse events in the salbutamol alone group compared with the IB + salbutamol group (RR 2.86; 95% CI 1.31–6.21; p = 0.008) (for details, refer to S8 Appendix).

### Secondary outcome

Unfortunately, owing to inconsistent reporting of outcomes in pulmonary function and clinical scores, a meta-analysis could not be performed. Therefore, a descriptive synthesis of aforementioned outcomes was performed instead.

Pulmonary function was reported in 5 studies (Table 1), among which three reported predicted % FEV_1_ at 60 min after treatment [21,30,32] with high quality evidence, one reported predicted % FEV_1_ at 120 min after treatment [32] with moderate quality evidence (S11 Appendix), one reported absolute % FEV_1_ at 60 min and 120 min after treatment [41] with low quality evidence, and one reported respiratory resistance at 60 and 120 min after treatment [28] with moderate quality evidence. Among the three studies reported predicted % FEV_1_, one study significantly favoring IB + salbutamol at 60 and 120 min after treatment [32] and the other two showed no significant difference at 60 min after treatment [21,30]. The study reported absolute % FEV_1_ at 60 min and 120 min [41] significantly favoring IB + salbutamol therapy at 120 min after treatment. The study reported respiratory resistance at 60 and 120 min [19,28] showed no significant difference between IB + salbutamol and salbutamol alone groups.

Nine studies reported clinical scores regarding different symptoms at various timepoints ranging from 15 min to 240 min, with one study did not report treatment duration [42] (S9 Appendix). Among them, one study reported dyspnea scores with moderate quality evidence [43]; one reported respiratory distress scores with moderate quality evidence [44]; five reported wheeze scores with low quality evidence [12,41–43,45]; two reported asthma scores with very low quality evidence [11,29]; two reported wheezing sound scores with very low quality evidence [41,43]; one reported cough scores with low quality evidence [43]; and one reported clinical scores based on clinical examination, activity, and speech with low quality evidence [46], with three of them significantly favoring IB + salbutamol therapy [11,42,43].

Regarding various specific adverse events (S9 Appendix), dry month [30,38,41,42,47,48], nausea [13,21,28,30,32,33], tremor [21,24,31,33,42,48,49], and vomiting [21,22,24,28,30,31,33,49] were reported in more than two trials. IB + salbutamol group showed significant reduction on the incidence of nausea compared with salbutamol alone group (RR 0.60; 95% CI 0.39, 0.93; p = 0.02; I^2^ = 0%; seven studies with 763 participants, high quality evidence). However, none of the other three outcomes showed significant differences between the two groups (p > 0.05) with moderate or low-quality evidence (S10 Appendix). In addition, there was also no significant difference in other adverse events (such as abdominal pain, headache, palpitations, etc) between the two groups (p > 0.05) with moderate or low quality evidence (for details, refer to S9 Appendix).

Additionally, there was no significant difference in oxygen saturation (p = 0.18, very low quality evidence), need for extra medication (repeated bronchodilator treatments (p = 0.32, moderate quality evidence), systemic corticosteroids (p = 0.41, moderate quality evidence)), and relapse rate (p = 0.85, moderate quality evidence) between the two groups (S9 Appendix).

### Sensitivity analysis

In sensitivity analysis omitting enrolled studies in turn, the results remained consistent across different analyses, which suggested that the findings were reliable and robust (for details, refer to S11 Appendix).

### Publication bias

Publication bias of the studies was assessed using funnel plots for hospital admission and relapse rates. The Eggers’ test indicated the presence of funnel plot asymmetry for hospital admission (t = -2.404, p = 0.03), but not for relapse rate (t = -0.851, p = 0.42) (S12 Appendix). The differences in methodological quality might be a potential source of funnel plot asymmetry in hospital admission. The quality of evidence in hospital admission was rated low since the lack of blinding information. In addition, the true heterogeneity in intervention effects may also be a potential source of asymmetry.

## Discussion

There is a lack of consistency in clinical practice regarding the treatment of asthma exacerbation in children and adolescents. Since the last systematic review published in 2013 [14], a considerable number of new studies evaluating the efficacy and safety of IB + salbutamol compared with those of salbutamol alone for the treatment of asthma exacerbation in children and adolescents have been published. This systematic review was conducted to update the findings on this topic and provide clinicians with the most current information to aid in the decision-making process involved in determining the best treatment options for the pediatric population presenting with acute asthma exacerbation.

### Summary of findings

The combination of IB + salbutamol showed significant reduction on hospital admission and nausea, but not on other adverse events including dry mouth, tremor and vomiting. From the descriptive synthesis, IB + salbutamol showed significant improvement on pulmonary function at 120 mins after treatment regarding the predict % FEV_1_ and absolute FEV_1_. The significant improvement on predict % FEV_1_ at 60 mins after treatment of IB + salbutamol was observed only in one study. The overall quality of evidence varied from moderate to very low.

### Comparisons with previous reviews

This systematic review supported the benefits of IB + salbutamol for the treatment of asthma in children and adolescents according to the reduction in hospital admission (RR 0.79; 95% CI 0.66–0.95). We performed subgroup analysis to determine whether age, severity of asthma, and co-intervention influenced the effect of IB + salbutamol treatment on hospital admission. Although the subgroup analyses might have contained overlap and non-randomized participants, the result could probably suggest the benefits in children and adolescents with severe asthma exacerbation (RR 0.71; 95% CI 0.60–0.85) and moderate-to-severe asthma exacerbation (RR 0.69; 95% CI 0.50–0.96), which is consistent with the results of a previous systematic review [14]. Consistent with the findings of Castro-Rodriguez (2015) [50], patient age did not alter the effect of IB + salbutamol on reduction of the risk of hospital admission. However, contrary to the findings of Griffiths (2013) [14], IB + salbutamol showed no significant reduction in the risk of hospital admission in patients with co-intervention of steroid. A possible explanation is the difference in interventions between the present review and Griffiths’ (2013) [14] review. The previous review reported a wider range of intervention that included all types of combined inhaled anticholinergics and SABAs, which may have included studies focused on terbutaline. However, the present review only included IB + salbutamol as an intervention treatment. Therefore, studies with a focus on terbutaline were excluded. Another explanation could be the updated search date. Compared with the review by Griffiths (2013) [14], the present review included additional 6-year literature published between 2013 and 2019. Moreover, Griffiths (2013) [14] used a fixed effects model to analyze data, whereas the present study used a random effects model. The use of different statistical models may also explain the difference in the results.

Consistent with previous systematic reviews [14,50], IB + salbutamol could significantly improve the predicted % and absolute % change in FEV_1_ at both 60 and 120 minutes after treatment compared with salbutamol alone. Contrary to the review by Griffiths (2013) [14], the increase in lung function observed with the combined treatment was not associated with an increase in oxygen saturation. A possible explanation for this is that the pervious review [14] used oxygen saturation < 95% instead of percentage of oxygen saturation as the outcome indicator.

Consistent with previous systematic review [14], nausea, vomiting, and tremors were listed as secondary outcomes because of the direct treatment-related effects of salbutamol or ipratropium bromide. Although the combination of IB and salbutamol was previously found to result in fewer tremors and less nausea compared with salbutamol alone [17], we found consistent results in nausea (RR 0.60; 95% CI 0.39–0.93) but did not identify any significant difference in the other three adverse events between the two groups. Possible explanations could be what has mentioned previously for subgroup results.

The present review found that most Chinese studies reported a clinical response as an outcome after treatment using IB + salbutamol or salbutamol alone in children and adolescents. However, because the clinical response was not clearly defined in the original studies, it was not included as a secondary outcome in the present review.

### Limitations

However, this systematic review has several limitations. Firstly, because of the different diagnostic criteria of childhood asthma, the external validity of the studies is quite poor. Secondly, the lack of random generation and blinding information, significant publication bias, and imprecision resulted a moderate to very low-quality of evidence. Thirdly, the applicability of results from the present review should be concluded with caution. The analyses of patients’ age, severity of asthma, and co-interventions were conducted with subgroup data from original studies and resulted consistent conclusion with 2013 review [14]. In addition, because of insufficient data, we were unable to perform subgroup analyses of other factors of interest, such as dosage regimens and frequency. Moreover, the treatment durations and phases across the included studies varied. The differences may also affect the applicability of the present review results. Although data extrapolation from the non-randomized subgroup population should be cautious, the current conclusion of our meta-analysis may provide new ideas and directions to identify the clinical beneficiaries of combination therapy of IB + salbutamol.

### Further direction

According to the moderate to very low quality of evidence, it could be suggested that further well-conducted and adequately powered RCTs with double-blind settings, large sample size, and standardized outcome measures are needed to evaluate the effectiveness of IB + salbutamol in treatment of asthma in children and adolescents. In addition, the treatment dosage, frequency and duration were varied in trials which may be a potential source of heterogeneity. Therefore, future studies may be suggested to explore the most appropriate treatment dosage and duration for children and adolescents with asthma.

## Conclusion

In conclusion, the results indicate that IB + salbutamol can significantly reduce the risk of hospital admission in children and adolescents, and this combined therapy may have significant clinical benefits in children with severe and moderate-to-severe asthma exacerbation. High quality evidence are required for future research studies in evaluating the clinical benefits of combining IB and salbutamol in asthma children and adolescent in different age, severity of asthma, and co-interventions subgroups.

## Supporting information

S1 Appendix PRISMA checklist. (PDF)

S2 Appendix Search strategy. (PDF)

S3 Appendix Subgroup details. (PDF)

S4 Appendix Full references. (PDF)

S5 Appendix Characteristics of included studies. (PDF)

S6 Appendix Quality of included studies. (PDF)

S7 Appendix Forest plots of hospital admission (subgroup). (PDF)

S8 Appendix Forest plots of any adverse event (subgroup). (PDF)

S9 Appendix Forest plots of secondary outcomes. (PDF)

S10 Appendix Summary of finding table. (PDF)

S11 Appendix Sensitivity analysis. (PDF)

S12 Appendix Publication bias. (PDF)

## References

[1] Masoli M, Fabian D, Holt S, Beasley R, Program GIfA. The global burden of asthma: executive summary of the GINA Dissemination Committee report. Allergy. 2004;59(5):469–78. 10.1111/j.1398-9995.2004.00526.x 15080825

[2] Zhou X, Hong J. Pediatric Asthma Management in China: Current and Future Challenges. Paediatr Drugs. 2018;20(2):105–10. 10.1007/s40272-017-0276-7 29222627

[3] Kamble S, Bharmal M. Incremental direct expenditure of treating asthma in the United States. J Asthma. 2009;46(1):73–80. 10.1080/02770900802503107 19191142

[4] Asher I, Pearce N. Global burden of asthma among children. Int J Tuberc Lung Dis. 2014;18(11):1269–78. 10.5588/ijtld.14.0170 25299857

[5] Bao Y, Chen Z, Liu E, Xiang L, Zhao D, Hong J. Risk Factors in Preschool Children for Predicting Asthma During the Preschool Age and the Early School Age: a Systematic Review and Meta-Analysis. Curr Allergy Asthma Rep. 2017;17(12):85 10.1007/s11882-017-0753-7 29151195

[6] Global Initiative for Asthma (GINA). 2019.

[7] National Asthma Education and Prevention Program: Expert Panel Report 3: Guidelines for the Diagnosis and Management of Asthma. NIH. 2007.

[8] Hong J, Bao Y, Chen A, Li C, Xiang L, Liu C, et al Chinese guidelines for childhood asthma 2016: Major updates, recommendations and key regional data. J Asthma. 2018;55(10):1138–46. 10.1080/02770903.2017.1396474 29227721

[9] Wang J. Efficacy of Ipratropium Bromide Combined with Salbutamol Atomization Inhalation in Treating Bronchial Asthma with Pulmonary Infection in Children. Journal of North Pharmacy. 2019(04):52–3.

[10] Qureshi F, Pestian J, Davis P, Zaritsky A. Effect of nebulized ipratropium on the hospitalization rates of children with asthma. N Engl J Med. 1998;339(15):1030–5. 10.1056/NEJM199810083391503 9761804

[11] Iramain R, Lopez-Herce J, Coronel J, Spitters C, Guggiari J, Bogado N. Inhaled salbutamol plus ipratropium in moderate and severe asthma crises in children. Journal of Asthma. 2011;48(3):298–303. 10.3109/02770903.2011.555037 21332430

[12] Memon BN, Parkash A, Ahmed Khan KM, Gowa MA, Bai C. Response to nebulized salbutamol versus combination with ipratropium bromide in children with acute severe asthma. J Pak Med Assoc. 2016;66(3):243–6. 26968269

[13] Watanasomsiri A, Phipatanakul W. Comparison of nebulized ipratropium bromide with salbutamol vs salbutamol alone in acute asthma exacerbation in children. Annals of Allergy, Asthma and Immunology. 2006;96(5):701–6. 10.1016/S1081-1206(10)61068-X 16729783

[14] Griffiths B, Ducharme FM. Combined inhaled anticholinergics and short-acting beta2-agonists for initial treatment of acute asthma in children. Paediatric Respiratory Reviews. 2013;14(4):234–5. 10.1016/j.prrv.2013.08.002 24070913

[15] Rodrigo GJ, Castro-Rodriguez JA. Anticholinergics in the treatment of children and adults with acute asthma: a systematic review with meta-analysis. Thorax. 2005;60(9):740–6. 10.1136/thx.2005.040444 16055613 PMC1747524

[16] Collaboration NCCTC. Review manager (RevMan)[computer program] Version 53. Copenhagen: The Nordic Cochrane Centre, The Cochrane Collaboration. 2014.

[17] Nibhanipudi K, Hassen G, Smith A. Beneficial effects of warmed humidified oxygen combined with nebulized albuterol and ipratropium in pediatric patients with acute exacerbation of asthma in winter months. Journal of emergency medicine [Internet]. 2009; 37(4):[446–50 pp.]. Available from: https://www.cochranelibrary.com/central/doi/10.1002/central/CN-00730185/full 19062230 10.1016/j.jemermed.2008.05.023

[18] Higgins J, Green S. Cochrane handbook for systematic reviews of interventions Version 5.1. 0 [updated March 2011]. London: The Cochrane Collaboration; 2011. 2016.

[19] GRADEpro G. GRADEpro guideline development tool [software]. McMaster University. 2015.

[20] Schünemann H, Brożek J, Guyatt G. GRADEpro GDT. GRADE Handbook, 2013. 2013.

[21] Peterson R WD, Mitchell I, Klassen T, Lamarre J, Rivard G, et al. Boehringer Ingelheim Trial No 2442430.3. Boehringer Ingelheim. 1994.

[22] Pharmaceuticals BI. A comparison of Combivent UDV (ipratropium 500mcg and salbutamol 2.5mg) and salbutamol UDV alone (2.5mg). Personal communication from Boehringer Ingelheim. 2009.

[23] Calvo GM, Calvo AM, Marin HF, Moya GJ. Is it useful to add an anticholinergic treatment to beta 2-adrenergic medication in acute asthma attack? J Investig Allergol Clin Immunol. 1998;8(1):30–4. 9555617

[24] Sharma A, Madaan A. Nebulized salbutamol vs salbutamol and ipratropium combination in asthma. Indian J Pediatr. 2004;71(2):121–4. 10.1007/BF02723090 15053373

[25] Zhu Q, Wu C, Xiao J, Cao F, Bai X. Effects of ipratropium bromide on pulmonary function, inflammatory factors and VEGF expression in asthmatic children. Practical Pharmacy and Clinical Remedies. 2019;22(06):625–8.

[26] Anthracopoulos MB, Karatza AA, Davlouros PA, Chiladakis JA, Manolis AS, Beratis NG. Effects of two nebulization regimens on heart rate variability during acute asthma exacerbations in children. Journal of Asthma. 2005;42(4):273–9. 10.1081/jas-200057895 16032936

[27] Benito Fernandez J, Mintegui Raso S, Sanchez Echaniz J, Vazquez Ronco MA, Pijoan Zubizarreta JI. Efficacy of early administration of nebulized ipratropium bromide in children with asthmatic crisis. An Esp Pediatr. 2000;53(3):217–22. 11083963

[28] Ducharme FM, Davis GM. Randomized controlled trial of ipratropium bromide and frequent low doses of salbutamol in the management of mild and moderate acute pediatric asthma. Journal of Pediatrics. 1998;133(4):479–85. 10.1016/s0022-3476(98)70054-x 9787684

[29] Kumaratne M, Gunawardane G. Addition of ipratropium to nebulized albuterol in children with acute asthma presenting to a pediatric office. Clinical Pediatrics. 2003;42(2):127–32. 10.1177/000992280304200205 12659385

[30] Qureshi F, Zaritsky A, Lakkis H. Efficacy of nebulized ipratropium in severely asthmatic children1997 1997 [cited RAYYAN-INCLUSION: {"Bei" = >"Included", "SITONG" = >"Included"} | RAYYAN-LABELS: Age | RAYYAN-EXCLUSION-REASONS: wrong population; 29(2):[205‐11 pp.]. https://www.cochranelibrary.com/central/doi/10.1002/central/CN-00136296/full. 10.1016/s0196-0644(97)70269-5 9018183

[31] Reisman J, Galdes-Sebalt M, Kazim F, Canny G, Levison H. Frequent administration by inhalation of salbutamol and ipratropium bromide in the initial management of severe acute asthma in children. Journal of Allergy and Clinical Immunology. 1988;81(1):16–20. 10.1016/0091-6749(88)90214-x 2963059

[32] Schuh S, Johnson DW, Callahan S, Canny G, Levison H. Efficacy of frequent nebulized ipratropium bromide added to frequent high-dose albuterol therapy in severe childhood asthma. Journal of Pediatrics. 1995;126(4):639–45. 10.1016/s0022-3476(95)70368-3 7699549

[33] Wyatt EL, Borland ML, Doyle SK, Geelhoed GC. Metered-dose inhaler ipratropium bromide in moderate acute asthma in children: A single-blinded randomised controlled trial. J Paediatr Child Health. 2015;51(2):192–8. 10.1111/jpc.12692 25039574

[34] Zorc JJ, Pusic MV, Ogborn CJ, Lebet R, Duggan AK. Ipratropium bromide added to asthma treatment in the pediatric emergency department. Pediatrics. 1999;103(4 Pt 1):748–52. 10.1542/peds.103.4.748 10103297

[35] Craven D, Kercsmar CM, Myers TR, O’Riordan MA, Golonka G, Moore S. Ipratropium bromide plus nebulized albuterol for the treatment of hospitalized children with acute asthma. Journal of Pediatrics. 2001;138(1):51–8. 10.1067/mpd.2001.110120 11148512

[36] Lin D. Budesonide combined salbutamol ipratropium bromide in infants with asthma clinical observation. CHINA CLINICAL PRACTICAL MEDICINE. 2010;04(7):106–7.

[37] Yi Y. Yi Bing Tuo Xiu An Lian He Sha Ding An Chun Zhi Liao Xiao Er Xiao Chuan De Liao Xiao Guan Cha [Observation of curative effect of ipratropium bromide combined with salbutamol on children with asthma]. The Journal of Medical Theory and Practice. 2015;28(12):1600–1.

[38] Yin Y. Yi Bing Tuo Xiu An Lian He Sha Ding An Chun Ji An Cha Jian Dui Xiao Chuan Huan Er Xue Qing Xi Bao Yin Zi Ji Fei Gong Neng De Ying Xiang [Effects of ipratropium bromide combined with salbutamol and aminophylline on serum cytokines and lung function in children with asthma]. Journal of North Pharmacy. 2018;15(03):38–9.

[39] Zhang Q. Sha Ding An Chun Lian He Yi Bing Tuo Xiu An Zhi Liao Xiao Er Xiao Chuan De Liao Xiao Guan Cha [Observation of curative effect of salbutamol combined with ipratropium bromide in the treatment of childhood asthma]. Medical Innovation of China. 2012;27:111–2.

[40] LIAO J, LIN J, PENG J. Feasibility of aerosol inhalation of ipratropium bromide in adjuvant treatment of children with asthma. China Medicine and Pharmacy. 2019;9(06):85–8.

[41] Li S, Huang T, Yuan Y, Chen Q, Chen Y. Xiu Hua Yi Bing Tuo Xiu An Lian He Sha Ding An Chun Shui Rong Ye Wu Hua Xi Ru Zhi Liao Er Tong Xiao Chuan Ji Xing Fa Zuo Liao Xiao Guan Cha [Observation of curative effect of ipratropium bromide combined with salbutamol aqueous solution inhalation in the treatment of acute asthma attacks in children]. Chinese Journal of Medicine. 2000;12.

[42] Luo X. Xiu Hua Yi Bing Tuo Pin Xi Ru Zhi Liao Xiao Er Ji Xing Zhi Qi Guan Xiao Chuan [Nebulized inhalation of ipratropium bromide in the treatment of acute bronchial asthma in children]. Modern Practical Medicine. 2004;07:400–1.

[43] Ji D, Wu Y, Luo Y, Li S. Yi Bing Tuo Xiu An Bu Di Nai De He Sha Ding An Chun Lian He Wu Hua Xi Ru Zhi Liao Er Tong Xiao Chuan Ji Xing Fa Zuo [Ipratropium bromide, budesonide and salbutamol combined with aerosol inhalation in the treatment of acute asthma attacks in children]. Chinese Journal of Coal Industry Medicine. 2003;12:1195–6.

[44] Coskun S, Yuksel H, Tikiz H, Danahaliloğlu S. Standard dose of inhaled albuterol significantly increases QT dispersion compared to low dose of albuterol plus ipratropium bromide therapy in moderate to severe acute asthma attacks in children2001 2001 [cited RAYYAN-INCLUSION: {"Bei" = >"Included", "SITONG" = >"Included"} | RAYYAN-LABELS: Unclear age; 43(6):[631‐6 pp.]. https://www.cochranelibrary.com/central/doi/10.1002/central/CN-00376178/full. 10.1046/j.1442-200x.2001.01471.x 11737740

[45] Chakraborti A, Lodha R, Pandey RM, Kabra SK. Randomized controlled trial of ipratropium bromide and salbutamol versus salbutamol alone in children with acute exacerbation of asthma. Indian J Pediatr. 2006;73(11):979–83. 10.1007/BF02758300 17127777

[46] Rayner RJ, Cartlidge PH, Upton CJ. Salbutamol and ipratropium in acute asthma. Arch Dis Child. 1987;62(8):840–1. 10.1136/adc.62.8.840 2959208 PMC1778468

[47] Dai J, Chen K, Zhang R, Fu Z, Xiong D, Li F, et al Yi Bing Tuo Xiu An Lian He Sha Ding An Chun Wu Hua Zhi Liao Er Tong Xiao Chuan [Treatment of childhood asthma with ipratropium bromide combined with salbutamol atomization]. CHINESE JOURNAL OF CONTEMPORARY PEDIATRICS. 2000;01:12–4.

[48] Watson WTA, Shuckett EP, Becker AB, Simons FER. Effect of nebulized ipratropium bromide on intraocular pressures in children. Chest. 1994;105(5):1439–41. 10.1378/chest.105.5.1439 8181333

[49] Beck R, Robertson C, Galdes-Sebaldt M, Levison H. Combined salbutamol and ipratropium bromide by inhalation in the treatment of severe acute asthma. Journal of Pediatrics. 1985;107(4):605–8. 10.1016/s0022-3476(85)80033-0 2931507

[50] Castro-Rodriguez JA, G JR, C ER-M. Principal findings of systematic reviews of acute asthma treatment in childhood. J Asthma. 2015;52(10):1038–45. 10.3109/02770903.2015.1033725 26303207

