# Supplementary material for: Combination of ipratropium bromide and salbutamol in children and adolescents with asthma: A meta-analysis
Source: PLoS One. 2021 Feb 23;16(2):e0237620. doi: 10.1371/journal.pone.0237620 (PMC7901745; doi:10.1371/journal.pone.0237620)
Supplement: S2 Appendix — (PDF) [file pone.0237620.s002.pdf]

A. Medline

1 exp asthma/ (128519)

2 respiratory sounds/ (9000)

3 bronchial spasm/ (4312)

4 exp bronchoconstriction/ (4145)

5 bronchial hyperreactivity/ (7362)

6 respiratory hypersensitivity/ (9629)

7 (asthma* or antiasthma* or anti-asthma* or wheez* or bronchospas* or bronchoconstrict*).tw,kw,kf. (173642)

8 (bronch* adj3 (spasm* or constrict*)).tw,kw,kf. (1214)

9 ((bronchial* or respiratory or airway* or lung*) adj3 (hypersensitiv* or hyperreactiv* or allerg* or insufficiency)).tw,kw,kf. (28880)

10 ((dust or mite*) adj3 (allerg* or hypersensitiv*)).tw,kw,kf. (5521)

11 or/1-10 (219304)

12 (ipratropium bromide adj3 (salbutamol or albuterol)).tw,kw,kf. (277)

13 (combiprasal or combivent* or duolin or duoneb or ipramol).tw,kw,kf. (28)

14 exp ipratropium/ and exp albuterol/ (592)

15 (ipratopium or aerovent or apo-ipravent or apovent or aproven or atem or atroaldo or atronase or atrovent or ipravent or ipraxa or ipvent or itrop or narilet or respontin or rinatec or sch 1000 or sch1000).tw,kw,kf. (330)

16 (salbutamol or aero clenil or ah 3365 or ah3365 or albuterol or almotex or asmadil or asmalin or asmasal or asmatol or asmaven or asmidon or asmol or broncho spray or bronter or butahale or buto-asma or butotal or butovent or buventol or cibutamol or cybutol or dilatamol or ecovent or farcolin or frespire or grafalin or loftan or mozal or novosalmol or parasma or proventil or repetabs or respolin or salamol or salbuair or salbulin or salbumol or salbupart or salbuvent or salden or salmol or saltos or solbutamol or spacehaler or sultanol or venetlin or ventilan or ventodisk* or ventol or volmac).tw,kw,kf. (9865)

17 15 and 16 (45)

18 or/12-14,17 (697)

19 11 and 18 (378)

20 exp child/ or exp child, preschool/ or exp pediatrics/ (1943289)

21 (child* or infant* or pediatric* or paediatric*).tw,kw,kf. (1881804)

22 or/20-21 (2762683)

23 19 and 22 (116)

24 exp clinical trial/ (870473)

25 exp randomized controlled trials/ (139682)

26 exp double-blind method/ (159976)

27	exp single-blind method/ (29077)
28	exp cross-over studies/ (48601)
29	randomized controlled trial.pt. (513863)
30	clinical trial.pt. (524970)
31	controlled clinical trial.pt. (93859)
32	(clinic* adj2 trial).mp. (729693)
33	(random* adj5 control* adj5 trial*).mp. (742876)
34	(crossover or cross-over).mp. (97648)
35	((singl* or doubl* or tripl* or trebl*) adj (blind* or mask*)).mp. (240052)
36	randomi*.mp. (907437)
37	(random* adj5 (assign* or allocat* or assort* or reciev*)).mp. (248520)
38	or/24-37 (1459550)
39	23 and 38 (77)
40	animals/ not humans/ (4704627)
41	39 not 40 (77)

B. Embase:

SEARCH QUERY

(((('asthma'/exp OR 'abnormal respiratory sound' OR 'bronchospasm' OR 'bronchoconstriction'/exp OR (bronchus AND hyperreactivity) OR 'respiratory tract allergy' OR (asthma* OR antiasthma* OR 'anti asthma*' OR wheez* OR bronchospas* OR bronchoconstrict*) OR (bronch* AND (spasm* OR constrict*)) OR ((bronchial* OR respiratory OR airway* OR lung*) AND (hypersensitiv* OR hyperreactiv* OR allerg* OR insufficiency)) OR ((dust OR mite*) AND (allerg* OR hypersensitiv*))) AND ('ipratropium bromide plus salbutamol'/exp OR (ipratropium AND bromide AND (salbutamol OR albuterol)) OR (combiprasal OR combivent* OR duolin OR duoneb OR ipramol) OR ('salbutamol'/exp AND 'ipratropium bromide'/exp) OR (((ipratopium OR aerovent OR 'apo ipravent' OR apovent OR aproven OR atem OR atroaldo OR atronase OR atrovent OR ipravent OR ipraxa OR ipvent OR itrop OR narilet OR respontin OR rinatec OR sch) AND 1000 OR sch1000) AND ('salbutamol'/exp OR salbutamol OR 'aero clenil'/exp OR 'aero clenil' OR (aero AND ('clenil'/exp OR clenil)) OR 'ah 3365'/exp OR 'ah 3365' OR (ah AND 3365) OR 'ah3365'/exp OR ah3365 OR 'albuterol'/exp OR albuterol OR 'almotex'/exp OR almotex OR 'asmadil'/exp OR asmadil OR 'asmalin'/exp OR asmalin OR 'asmasal'/exp OR asmasal OR 'asmatol'/exp OR asmatol OR 'asmaven'/exp OR asmaven OR 'asmidon'/exp OR asmidon OR 'asmol'/exp OR asmol OR 'broncho spray'/exp OR 'broncho spray' OR (broncho AND ('spray'/exp OR spray)) OR 'bronter'/exp OR bronter OR 'butahale'/exp OR butahale OR 'buto asma'/exp OR 'buto asma' OR 'butotal'/exp OR butotal OR 'butovent'/exp OR butovent OR 'buventol'/exp OR buventol OR 'cibutamol'/exp OR cibutamol OR 'cybutol'/exp OR cybutol OR 'dilatamol'/exp OR dilatamol OR 'ecovent'/exp OR ecovent OR 'farcolin'/exp OR farcolin OR 'frespire'/exp OR frespire OR 'grafalin'/exp OR grafalin OR 'loftan'/exp OR loftan OR 'mozal'/exp OR mozal OR 'novosalmol'/exp OR novosalmol OR 'parasma'/exp OR parasma OR 'proventil'/exp OR proventil OR 'repetabs'/exp OR repetabs OR 'respolin'/exp

OR respolin OR 'salamol'/exp OR salamol OR 'salbuair'/exp OR salbuair OR 'salbulin'/exp OR salbulin OR 'salbumol'/exp OR salbumol OR 'salbupart'/exp OR salbupart OR 'salbuvent'/exp OR salbuvent OR 'salden'/exp OR salden OR 'salmol'/exp OR salmol OR 'saltos'/exp OR saltos OR 'solbutamol'/exp OR solbutamol OR 'spacehaler'/exp OR spacehaler OR 'sultanol'/exp OR sultanol OR 'venetlin'/exp OR venetlin OR 'ventilan'/exp OR ventilan OR ventodisk* OR 'ventol'/exp OR ventol OR 'volmac'/exp OR volmac)))) AND (('child'/exp OR 'infant'/exp OR 'pediatrics'/exp) OR (child* OR infant* OR pediatric* OR paediatric*))) AND ((clin* AND trial) OR ((singl* OR doubl* OR trebl* OR tripl*) AND (blind* OR mask*)) OR (random* AND (assign* OR allocat*)) OR randomi* OR crossover OR 'randomized controlled trial'/exp OR 'double blind procedure'/exp OR 'crossover procedure'/exp OR 'single blind procedure'/exp OR 'randomization'/exp)) NOT (('animal'/exp OR 'invertebrate'/exp OR ('animal experiment' OR 'animal model' OR 'animal tissue' OR 'animal cell' OR nonhuman)) NOT (('animal'/exp OR 'invertebrate'/exp OR ('animal experiment' OR 'animal model' OR 'animal tissue' OR 'animal cell' OR nonhuman)) AND (human OR 'normal human' OR 'human cell'))) 352

C. the Cochrane Library

#1 MeSH descriptor: [Asthma] explode all trees 11640

#2 MeSH descriptor: [Respiratory Sounds] explode all trees625

#3 MeSH descriptor: [Bronchial Spasm] explode all trees 391

#4 MeSH descriptor: [Bronchoconstriction] explode all trees 561

#5 MeSH descriptor: [Bronchial Hyperreactivity] explode all trees 578

#6 MeSH descriptor: [Respiratory Hypersensitivity] explode all trees 14512

#7 (asthma* or antiasthma* or "anti-asthma*" or wheez* or bronchospas* or bronchoconstrict):ti,ab,kw (Word variations have been searched) 35870

#8 (bronch* NEAR/3 (spasm* or constrict*)):ti,ab,kw (Word variations have been searched) 492

#9 ((bronchial* or respiratory or airway* or lung*) NEAR/3 (hypersensitiv* or hyperreactiv* or allerg* or insufficiency)):ti,ab,kw (Word variations have been searched) 4655

#10 ((dust or mite*) NEAR/3 (allerg* or hypersensitiv*)):ti,ab,kw (Word variations have been searched) 1045

#11 #1 or #2 or #3 or #4 or #5 or #6 or #7 or #8 or #9 or #10 41569

#12 ("ipratropium bromide" NEAR/3 (salbutamol or albuterol)):ti,ab,kw (Word variations have been searched) 339

#13 (combiprasal or combivent* or duolin or duoneb or ipramol):ti,ab,kw (Word variations have been searched) 59

#14 MeSH descriptor: [Ipratropium] explode all trees 805

#15 MeSH descriptor: [Albuterol] explode all trees 3417

#16 #14 and #15 331

#17 (ipratopium or aerovent or apo-ipravent or apovent or aproven or atem or atroaldo or atronase or atrovent or ipravent or ipraxa or ipvent or itrop or narilet or respontin or rinatec or "sch 1000" or sch1000):ti,ab,kw (Word variations have been searched) 269

#18 (salbutamol or aero clenil or ah 3365 or ah3365 or albuterol or almotex or asmadil or asmalin or asmasal or asmatol or asmaven or asmidon or asmol or broncho spray or bronter or butahale or buto-asma or butotal or butovent or buventol or cibutamol or cybutol or dilatamol or ecovent or farcolin or frespire or grafalin or loftan or mozal or novosalmol or parasma or proventil or repetabs or respolin or salamol or salbuair or salbulin or salbumol or salbupart or salbuvent or salden or salmol or saltos or solbutamol or spacehaler or sultanol or venetlin or ventilan or ventodisk* or ventol or volmac):ti,ab,kw (Word variations have been searched) 6682

#19 #17 and #18 50

#20 #12 or #13 or #16 or #19 589

#21 #11 and #20 328

#22 MeSH descriptor: [Child] explode all trees 55220

#23 MeSH descriptor: [Child, Preschool] explode all trees 29107

#24 MeSH descriptor: [Pediatrics] explode all trees 669

#25 (child* or infant* or pediatric* or paediatric*):ti,ab,kw (Word variations have been searched) 188608

#26 #22 or #23 or #24 or #25 188619

#27 #21 and #26 in Trials 127

D. CBM

("异丙托溴铵"[摘要:智能] OR "溴异丙托品"[摘要:智能] OR "溴化异丙阿托品"[摘要:智能] OR "异丙托品"[摘要:智能] OR "爱全乐"[摘要:智能]) AND("沙丁胺醇"[摘要:智能] OR "羟甲叔丁肾上腺素"[摘要:智能] OR "柳丁氨醇"[摘要:智能] OR "万托林"[摘要:智能]) AND("患儿"[摘要:智能] OR "小儿"[摘要:智能] OR "儿童"[摘要:智能] OR "儿童"[不加权:扩展]) AND (" 哮喘"[不加权:扩展]) 181 条

E. CNKI

(SU=异丙托溴铵 OR SU=溴异丙托品 OR SU=溴化异丙阿托品 OR SU=异丙托品 OR SU=爱全乐) AND (SU=沙丁胺醇 OR SU=羟甲叔丁肾上腺素 OR SU=柳丁氨醇 OR SU=万托林) AND (SU=患儿 OR SU=小儿 OR SU=儿童) AND SU=哮喘

93 条

F. VIP

R=(患儿 OR 小儿 OR 儿童) AND R=(异丙托溴铵 OR 溴异丙托品 OR 溴化异丙阿托品 OR 异丙托品 OR 爱全乐) AND R=("哮喘") AND R=(沙丁胺醇 OR 羟甲叔丁肾上腺素 OR 柳丁氨醇 OR 万托林)

136 条

G. Wangfang

((摘要:("患儿")+摘要:("小儿")+摘要:("儿童"))*(摘要:(异丙托溴铵)+摘要:(溴异丙托品)+摘要:(溴化异丙阿托品)+摘要:(异丙托品)+摘要:(爱全乐))*摘要:("哮喘")*(摘要:(沙丁胺醇)+摘要:(羟甲叔丁肾上腺素)+摘要:(柳丁氨醇)+摘要:(万托林)))

90 条
