# Supplementary material for: Combination of ipratropium bromide and salbutamol in children and adolescents with asthma: A meta-analysis
Source: PLoS One. 2021 Feb 23;16(2):e0237620. doi: 10.1371/journal.pone.0237620 (PMC7901745; doi:10.1371/journal.pone.0237620)
Supplement: S3 Appendix — (PDF) [file pone.0237620.s003.pdf]

Appendix 3 Subgroup of included studies

	Study ID	Subgroup (age)	Subgroup (severity)	Subgroup (co-intervention)
1	Anthracopoulos 2005	≥6	Severe	Unclear
2	Beck 1985	Mixed; ≥6	Severe	Unclear
3	Calvo 1998	Mixed	Unclear	Unclear
4	Chakraborti 2006	Mixed	Mixed	Unclear
5	Chen 2010	Mixed	Severe	With steroid
6	Coskun 2001	Mixed	Moderate to severe	Unclear
7	Craven 2001	Mixed	Mixed	With steroid
8	Dai 2000	≥6	Unclear	Without steroid
9	Ding 2010	≥6	Unclear	Unclear
10	Ducbarme 1998	Mixed	Moderate to severe	Mixed
11	Guo 2015	≥6	Unclear	With steroid
12	He 2011	≥6	Unclear	With steroid
13	Iramain 2011	Mixed	Moderate to severe	Unclear
14	Ji 2003	Mixed	Unclear	With steroid
15	Kong 2003	Mixed	Unclear	With standard care
16	Kumaratne 2003	Mixed	Mild to moderate	Unclear
17	Li 2000	≥6	Moderate to severe	Unclear
18	Li 2011	Mixed	Unclear	With standard care
19	Liang 2018	< 6	Unclear	With standard care
20	Liao 2019	≥6	Mixed	With steroid
21	Lin 2010	< 6	Unclear	With steroid

22	Liu 2012	Mixed	Unclear	With steroid
23	Liu 2016	Mixed	Unclear	Unclear
24	Luo 2004	Unclear	Unclear	With steroid
25	Luo 2014	Mixed	Unclear	With standard care
26	Memon 2016	Mixed	Severe	Unclear
27	Ni 2003	Mixed	Unclear	With steroid
28	Nibhanipudi 2009	≥6	Unclear	Unclear
29	Nong 2011	< 6	Unclear	With standard care
30	Pang 2014	Mixed	Unclear	With standard care
31	Qureshi 1997	Mixed	Unclear	With steroid
32	Qureshi 1998	Mixed	Moderate; Severe	With steroid
33	Rayner 1987	Mixed	Unclear	Mixed
34	Reisman 1988	Mixed	Severe	Unclear
35	Schuh 1995	Mixed	Severe	Without steroid
36	Sha 2011	< 6	Unclear	With steroid
37	Sharma 2004	≥6	Severe	Unclear
38	Sienra Monge 2000	Mixed	Unclear	Unclear
39	Storr 1986	Mixed	Mixed	With steroid
40	Wang 2019	< 6	Moderate to severe	With standard care
41	Wang 2019a	Mixed	Unclear	Unclear
42	Watanasomsiri 2006	Mixed	Moderate to severe	With steroid
43	Watson 1994	Mixed; ≥6	Moderate to severe	Unclear
44	Wu 2009	< 6	Moderate	With steroid

45	Wyatt 2015	Mixed	Moderate	With steroid
46	Yi 2015	≥6	Unclear	With standard care
47	Yin 2014	Mixed	Unclear	With standard care
48	Yin 2018	Mixed	Unclear	Unclear
49	Yuksel 2001	＜6	Unclear	Unclear
50	Zhang 2012	Mixed	Unclear	With standard care
51	Zhu 2019	≥6	Unclear	With standard care
52	Zorc 1999	Mixed	Mild/Moderate/Severe	With steroid
53	Benito Femandeza 2000	Mixed	Severe	With steroid
54	BI [pers comm]	Mixed	Severe	Without steroid
55	Peterson 1996	Mixed	Moderate to severe	With steroid

Mixed = the study contained a mix of two and more subgroups but did not provide split data.

Unclear = the study did not state relevant information about subgroups.
