# Supplementary material for: Combination of ipratropium bromide and salbutamol in children and adolescents with asthma: A meta-analysis
Source: PLoS One. 2021 Feb 23;16(2):e0237620. doi: 10.1371/journal.pone.0237620 (PMC7901745; doi:10.1371/journal.pone.0237620)
Supplement: S4 Appendix — (PDF) [file pone.0237620.s004.pdf]

**Appendix 4 References of included studies**

Anthracopoulos MB, Karatza AA, Davlouros PA, Chiladakis JA, Manolis AS, Beratis NG. Effects of two nebulization regimens on heart rate variability during acute asthma exacerbations in children. *Journal of Asthma.* 2005;42(4):273-279.

Beck R, Robertson C, Galdes-Sebaldt M, Levison H. Combined salbutamol and ipratropium bromide by inhalation in the treatment of severe acute asthma. *Journal of Pediatrics.* 1985;107(4):605-608.

Benito Fernandez J, Mintegui Raso S, Sanchez Echaniz J, Vazquez Ronco MA, Pijoan Zubizarreta JI. Efficacy of early administration of nebulized ipratropium bromide in children with acute asthma attack. [English]. *Anales Espanoles de Pediatria.* 2000;53(3):217-222.

Calvo GM, Calvo AM, Marin HF, Moya GJ. Is it useful to add an anticholinergic treatment to beta 2-adrenergic medication in acute asthma attack? *Journal of Investigational Allergology & Clinical Immunology.* 1998;8(1):30-34.

Chakraborti A, Lodha R, Pandey RM, Kabra SK. Randomized controlled trial of ipratropium bromide and salbutamol versus salbutamol alone in children with acute exacerbation of asthma. *Indian journal of pediatrics.* 2006;73(11):979-983.

Coskun S, Yuksel H, Tikiz H, Danahaliloğlu S. Standard dose of inhaled albuterol significantly increases QT dispersion compared to low dose of albuterol plus ipratropium bromide therapy in moderate to severe acute asthma attacks in children. *Pediatrics international.* 2001;43(6):631-636.

Craven D, Kercsmar CM, Myers TR, O'Riordan MA, Golonka G, Moore S. Ipratropium bromide plus nebulized albuterol for the treatment of hospitalized children with acute asthma. *Journal of Pediatrics.* 2001;138(1):51-58.

Ducharme FM, Davis GM. Randomized controlled trial of ipratropium bromide and frequent low doses of salbutamol in the management of mild and moderate acute pediatric asthma. *Journal of Pediatrics.* 1998;133(4):479-485.

Iramain R, Lopez-Herce J, Coronel J, Spitters C, Guggiari J, Bogado N. Inhaled salbutamol plus ipratropium in moderate and severe asthma crises in children. *Journal of Asthma.* 2011;48(3):298-303.

Kumaratne M, Gunawardane G. Addition of ipratropium to nebulized albuterol in children with acute asthma presenting to a pediatric office. *Clinical Pediatrics.* 2003;42(2):127-132.

Memon BN, Parkash A, Khan KMA, Gowa MA, Bai C. Response to nebulized salbutamol versus combination with ipratropium bromide in children with acute severe asthma. *Journal of the Pakistan Medical Association.* 2016;66(3):243-246.

Nibhanipudi K, Hassen G, Smith A. Beneficial effects of warmed humidified oxygen combined with nebulized albuterol and ipratropium in pediatric patients with acute exacerbation of asthma in winter months. *Journal of emergency medicine.* 2009;37(4):446-450.

Peterson R WD, Mitchell I, Klassen T, Lamarre J, Rivard G, et al. Boehringer Ingelheim Trial No 2442430.3. *Boehringer Ingelheim.* 1994.

Pharmaceuticals BI. A comparison of Combivent UDV (ipratropium 500mcg and salbutamol 2.5mg) and salbutamol UDV alone (2.5mg). *Personal communication from Boehringer Ingelheim.* 2009.

Qureshi F, Zaritsky A, Lakkis H. Efficacy of nebulized ipratropium in severely asthmatic children. *Annals of emergency medicine.* 1997;29(2):205-211.

Qureshi F, Pestian J, Davis P, Zaritsky A. Effect of nebulized ipratropium on the hospitalization rates of children with asthma. *New England Journal of Medicine.* 1998;339(15):1030-1035.

Rayner RJ, Cartlidge PH, Upton CJ. Salbutamol and ipratropium in acute asthma. *Archives of Disease in Childhood.* 1987;62(8):840-841.

Reisman J, Galdes-Sebalt M, Kazim F, Canny G, Levison H. Frequent administration by inhalation of salbutamol and ipratropium bromide in the initial management of severe acute asthma in children. *Journal of Allergy and Clinical Immunology.* 1988;81(1):16-20.

Sienra MJ, Bermejo GM, del RNB, Rosas VM, Reyes RN. Degree and duration of bronchodilatation with an agonist beta 2 administered alone versus an agonist beta 2 administered with ipratropium bromide in children with acute asthma. *Revista alergia mexico (tecamachalco, puebla, mexico : 1993).* 2000;47(1):26-29.

Schuh S, Johnson DW, Callahan S, Canny G, Levison H. Efficacy of frequent nebulized ipratropium bromide added to frequent high-dose albuterol therapy in severe childhood asthma. *Journal of Pediatrics.* 1995;126(4):639-645.

Sharma A, Madaan A. Nebulized salbutamol vs salbutamol and ipratropium combination in asthma. *Indian Journal of Pediatrics.* 2004;71(2):121-124.

Storr J, Lenney W. Nebulised ipratropium and salbutamol in asthma. *Archives of Disease in Childhood.* 1986;61(6):602-603.

Watanasomsiri A, Phipatanakul W. Comparison of nebulized ipratropium bromide with salbutamol vs salbutamol alone in acute asthma exacerbation in children. *Annals of Allergy, Asthma and Immunology.* 2006;96(5):701-706.

Watson WTA, Shuckett EP, Becker AB, Simons FER. Effect of nebulized ipratropium bromide on intraocular pressures in children. *Chest.* 1994;105(5):1439-1441.

Wyatt EL, Borland ML, Doyle SK, Geelhoed GC. Metered-dose inhaler ipratropium bromide in moderate acute asthma in children: A single-blinded randomised controlled trial. *Journal of Paediatrics & Child Health.* 2015;51(2):192-198.

Yuksel H, Coskun S, Polat M, Onag A. Lower arrythmogenic risk of low dose albuterol plus ipratropium. *Indian Journal of Pediatrics.* 2001;68(10):945-949.

Zorc JJ, Pusic MV, Ogborn CJ, Lebet R, Duggan AK. Ipratropium bromide added to asthma treatment in the pediatric emergency department. *Pediatrics.* 1999;103(4 Pt 1):748-752.

丁妞. 沙丁胺醇联合异丙托溴铵治疗儿童哮喘的临床疗效观察. *实用预防医学.* 2010;17(12).

何山. 氧驱动布地奈德与复方异丙托溴铵雾化吸入治疗儿童哮喘发作 61 例疗效观察. *临床和实验医学杂志.* 2011;10(18).

倪树芳, 姚文江. 沙丁胺醇联合异丙托溴铵治疗小儿哮喘 55 例. *医药导报.* 2003;22(2).

农冠荣, 黄梅青. 沙丁胺醇与异丙托溴铵联合治疗婴幼儿哮喘的疗效观察. *中外健康文摘.* 2011;08(16):78-79.

刘彩霞, 景卫利, 程雪, 罗卉丽, 黄梅. 异丙托溴铵对小儿哮喘患者白细胞介素 13、转化生长因子 β1 及血管内皮生长因子表达的影响. *中国医药导报*. 2016;13(18).

刘敏茹, 姜亚平. 布地奈德、沙丁胺醇与异丙托溴铵溶液配伍雾化吸入治疗儿童哮喘急性发作期 62 例疗效观察. *陕西医学杂志*. 2012(9).

吴慧芬, 洪佳璇. 不同药物联合吸入治疗儿童哮喘急性发作疗效比较. *浙江医学*. 2009;31(2).

姬东霞, 吴育雄, 罗宇元, 李珊. 异丙托溴铵、布地奈德和沙丁胺醇联合雾化吸入治疗儿童哮喘急性发作. *中国煤炭工业医学杂志*. 2003;6(12):1195-1196.

孔云. 沙丁胺醇与溴化异丙阿托品联合雾化吸入治疗儿童哮喘急性发作疗效观察. *中国基层医药*. 2003(04):61.

尹延凤. 肺炎支原体感染与儿童哮喘的相关性和临床疗效分析. *热带医学杂志*. 2014;14(5).

尹鋆. 异丙托溴铵联合沙丁胺醇及氨茶碱对哮喘患儿血清细胞因子及肺功能的影响. *北方药学*. 2018;15(3).

庞青卫. 观察氧气驱动万托林及爱全乐雾化吸入治疗小儿哮喘的疗效. *医学信息*. 2014(17):436-437.

廖九祥, 林进生, 彭锦英. 异丙托溴铵雾化吸入辅助治疗儿童哮喘的可行性. *中国医药科学*. 2019;009(6).

张琴. 沙丁胺醇联合异丙托溴铵治疗小儿哮喘的疗效观察. *中国医学创新*. 2012;9(27).

戴继宏, 陈坤华, 张儒谊, et al. 溴化异丙托品联合沙丁胺醇雾化治疗儿童哮喘. *中国当代儿科杂志*. 2000;2(1).

易宜群. 异丙托溴铵联合沙丁胺醇治疗小儿哮喘的疗效观察. *医学理论与实践*. 2015;28(12).

朱乔波, 吴春红, 肖佳荔, 曹芳, 白星. 加用异丙托溴铵对哮喘患儿肺功能、炎症因子及 VEGF 表达的影响. *实用药物与临床*. 2019;022(6).

李敏, 杨映天, 张丹. 万托林联合爱全乐氧雾治疗儿童哮喘症急性发作的疗效观察. *中国医药指南*. 2011;9(12).

李硕, 黄庭宏, 袁艺, 陈琪, 陈育智. 溴化异丙托品联合沙丁胺醇水溶液雾化吸入治疗儿童哮喘急性发作疗效观察. *中国医刊*. 2000;35(12):25-26.

林东浩. 沙丁胺醇布地奈德联合异丙托溴铵治疗婴幼儿哮喘的临床观察. *中国临床实用医学*. 2010(7).

梁晖. 沙丁胺醇及异丙托溴铵联合雾化吸入治疗小儿哮喘的疗效. *中国冶金工业医学杂志*. 2018;35(1).

沙吉达. 布地奈德加沙丁胺醇及异丙托溴铵雾化吸入治疗小儿哮喘的疗效观察. *心理医生：下*. 2011(8).

王晶晶. 异丙托溴铵联合沙丁胺醇雾化吸入治疗支气管哮喘伴肺部感染患儿的疗效. *北方药学*. 2019;0(4).

王武明, 邓春晖, 慈美荣, 刘小庆, 苏培媛, 谭静. 异丙托溴铵联合沙丁胺醇和二羟丙茶碱对小儿哮喘肺功能和气道炎性介质的改善效果分析. *中国医学前沿杂志（电子版）.* 2019;11(7):105-108.

罗惠琴, 张松丽, 陈国华, 刘国清. 异丙托溴铵联合沙丁胺醇治疗小儿哮喘的效果观察. *中国当代医药.* 2014;21(8).

罗秀员. 溴化异丙托品雾化吸入治疗小儿急性支气管哮喘. *现代实用医学.* 2004;16(7).

郭凤仙, 张玲, 田启运. 雾化吸入复方异丙托溴铵和布地奈德辅助治疗儿童哮喘急性发作疗效观察. *中国现代药物应用.* 2015;9(5).

陈保安, 罗会玉. 复方异丙托溴铵联合布地奈德雾化吸入治疗儿童哮喘重度发作. *河南职工医学院学报.* 2010;22(5).
