# Supplementary material for: Combination of ipratropium bromide and salbutamol in children and adolescents with asthma: A meta-analysis
Source: PLoS One. 2021 Feb 23;16(2):e0237620. doi: 10.1371/journal.pone.0237620 (PMC7901745; doi:10.1371/journal.pone.0237620)
Supplement: S5 Appendix — (PDF) [file pone.0237620.s005.pdf]

Appendix 5 Characteristics of included studies

Study ID	Design/ Location	Diagnosis	No. of patients	Age (mean, yrs)	Setting	Length of illness (mean, yrs)	Dosage of IB+Salbutamol	Dosage of Salbutamol	Duration of treatment	Co-intervention	Outcomes
Anthracopoulos 2005	Greece	Asthma	20	9.7	hospital (outpatient)	NR	IB: 5 µg/kg/dose (max: 250µg) Salbutamol: 0.1mg/kg/dose (max: 5mg)	Salbutamol: 0.15mg/kg/dose (max: 5mg)	60 minutes/every 20 minutes	NR	Hospital admission; Change in % predicted FEV_1
Beck 1985	Canada	Asthma	25	10.8	hospital (outpatient)	7.2	Salbutamol: initial dose of 150µg/kg(max 5mg) followed by 50µg/kg(max 1.7mg); IB: 250µg	Salbutamol: initial dose of 150µg/kg(max 5mg) followed by 50µg/kg(max 1.7mg); 0.9% saline: 1.0ml	Salbutamol: an initial dose followed by six consecutive doses at 20-minute intervals. Ipratropium bromide: at 60 minutes.	NR	Change in % predicted FEV_1; Specific adverse events
Calvo 1998	Chile	Asthma	80	NR	NR	NR	IB: 40 mcg Salbutamol: 200 mcg	Salbutamol: 200 mcg	q15 minutes x 4 then q20 minutes x 3	Some children received systemic corticosteroids (prednisone 1 mg/kg/dose: max = 40 mg) at 60 minutes after beginning treatment if "no clinical or	Hospital admission; Specific adverse events; Change in clinical score

										laboratory improvement”	
Chakraborti 2006	India	Asthma	60	9.3	hospital (non-specific)	4.4	IB: 80 µg<br>Salbutamol: 400 µg	Placebo: 80 µg<br>Salbutamol: 400 µg	NR	NR	Change in % predicted FEV_1; Clinical score; Need for repeated bronchodilator treatments
Chen 2010	China	Asthma	68	8.6	NR	NR	Budesonide: 2 ml<br>Compound IB: NR	Salbutamol: 0.5 ml<br>Budesonide: 2 ml	Twice daily, 5-10 minuts/time	Patients received regular treatment such as oxygen inhalation, sodium hydrocortisone succinate intravenous and antiviral therapy.	None
Coskun 2001	Turkey	Asthma	43	7	hospital (outpatient)	NR	IB: 250 µg<br>Albuterol: 0.075 mg/kg, maximum 2.5 mg	Albuterol: 0.15 mg/kg, maximum 2.5 mg (at start), followed with 0.1 mg/kg (repeated twice)	60 minutes/every 20 minutes	NR	Change in respiratory resistance; Oxygen saturation
Craven 2001	the United States	Acute asthma	210	Median 4.2	hospital (inpatient)	NR	Salbutamol: 2.5 mg;<br>IB: 250 µg (1.25 cc)	Salbutamol: 2.5 mg<br>1.25 cc sterile preservative	40 weeks<br>Phase I: every 4h;<br>Phase II: every	NR	Lengths of hospital stay; Any adverse events

								free isotonic saline solution	6h;<br>Phase III: every 8h.		
Dai 2000	China	Asthma	20	NR	hospital (inpatient)	NR	Salbutamol: 0.15 mg/kg,maximum dose:5 mg, for the1st time;<br>Salbutamol: 0.05 mg/kg, maximum dose:1.7 mg, for the 2nd/3rd/4th times;<br>IB: 0.25mg/kg, for the 4th time;<br>Normal saline was added to each treatment dose to achieve a total volume of 2mL.	Salbutamol: 0.15 mg/kg, maximum dose: 5 mg, for the1st time<br>Salbutamol: 0.05 mg/kg, maximum dose: 1.7 mg, for the 2nd/3rd/4th times.<br>Normal saline was added to each treatment dose to achieve a total volume of 2 mL.	4 times at 20 min intervals	NR	Specific adverse events
Ding 2010	China	Asthma	92	NR	hospital (non-specific)	NR	Salbutamol: 0.03 ml/kg;<br>IB: 1 mL	Salbutamol: 0.03 ml/kg	3-7 days, 2 times/day	Standard care	Clinical response
Ducbarme 1998	Canada	Asthma	275	NR	hospital (non-specific)	NR	3mL (with added saline) Salbutamol: 0.15 mg/kg (max. 5 mg);	3mL (with added saline) Salbutamol: 0.15 mg/kg (max. 5 mg)	60 minutes/every 30 mins	NR	Hospital admission;<br>Clinical response;<br>Oxygen saturation;

							IB: 1 mL (250µg)				Need for systemic corticosteroids; Relapse rate
Guo 2015	China	Asthma	136	NR	hospital (non-specific)	NR	2mL	0.5mL	3-5 days, 15 min/day; 2 times/day	Standard care + Budesonide (2 mL)	Clinical response
He 2011	China	Asthma	122	NR	hospital (non-specific)	1.4	Compound IB: 2.5 ml Budesonide: 2 ml	Salbutamol: 0.5 ml Budesonide: 2 ml	Twice daily	Patients recieved regular treatment, such as oxygen inhalation and antibiotics against infection.	Clinical response
Iramain 2011	Spain	Asthma	106	9.1	hospital (outpatient)	NR	IB: 250 µg(less than 20 kg); 500 µg(over 20 kg) Salbutamol: 2.5 mg(less than 20 kg); 5 mg(over 20 kg)	Salbutamol: 2.5 mg(less than 20 kg); 5 mg(over 20 kg)	120 minutes/every 20 minutes	NR	Hospital admission; Change in % predicted FEV_1; Change in clinical score; Oxygen saturation
Ji 2003	China	Acute asthma	70	NR	hospital (outpatient)	NR	Salbutamol: <4 yrs, 0.25mL; 4-8 yrs, 0.5mL; 8-12 yrs, 0.75mL. IB: <4 yrs, 0.5mL; 4-8 yrs, 1mL;	Salbutamol: <4 yrs, 0.25mL; 4-8 yrs, 0.5mL; 8-12 yrs, 0.75mL.	30 minutes/2 times/day	Budesonide 1mg, 2 times/day	Change in clinical score

							8-12 yrs, 1.5mL.				
Kong 2003	China	Acute asthma	60	NR	hospital (inpatient and/or outpatient)	NR	2.5mL (with added saline). Salbutamol: <4 yrs, 0.25mL; 4-8 yrs, 0.5mL; 8-12 yrs, 0.75mL. IB: <3 yrs, 0.5mL; 3-6 yrs, 0.75mL; >6 yrs, 1mL.	Salbutamol: 2.5mL (with added saline): <4 yrs, 0.25mL; 4-8 yrs, 0.5mL; 8-12 yrs, 0.75mL.	60 minutes/10 min/time, 2-3 time/day	Standard care	Clinical response
Kumaratne 2003	California	Acute asthma	50	1.95	community	NR	4 mL (with added saline) Salbutamol: 0.15 mg/kg; IB: 250 µg(under 15 kg); 500 µg(over 15 kg)	Salbutamol: 0.15 mg/kg	2-4L/min, 20min/time	NR	Hospital admission; Change in clinical score; Relapse rate
Li 2000	China	Acute Asthma	40	9.3	hospital (outpatient)	NR	IB: 0.25 - 0.5 mg Salbutamol: 2.5-5 mg	Salbutamol: 2.5-5 mg The above drugs should be adjusted appropriately according to patient's age.	5-10 minuts	NR	Percent change in FEV_1; Change in clinical score; Specific adverse events
Li 2011	China	Acute asthma	68	NR	hospital (non-specific)	NR	Saline: 1.75mL; Salbutamol:	Saline: 1.75mL; Salbutamol: 0.25mL.	3-5 days/10-15 min/time, 3 times/day	Standard care	Length of hospital stay;

							0.25mL;<br>IB: 0.4mL.				Clinical response
Liang 2018	China	Asthma	80	1.72	hospital (non-specific)	0.13	IB: 0.06 mg/kg; Salbutamol: 0.3 mg/kg	Salbutamol: 0.3 mg/kg	5 days/1 time/day	Standard care	Clinical response
Liao 2019	China	Asthma	132	8.9	hospital (non-specific)	0.17	Salbutamol: 10mL;<br>IB: 0.5mL	Salbutamol: 10mL	7 days/6 hours/time	Budesonide 1mg	Any adverse events;<br>Clinical response;<br>Change in % predicted FEV_1;<br>Change in clinical score
Lin 2010	China	Acute asthma	100	2.1	hospital (inpatient)	NR	Salbutamol:<br><4 yrs, 0.25mL;<br>4-8 yrs, 0.5mL.<br>IB:<br><4 yrs, 0.5mL;<br>4-8 yrs, 1mL.	Salbutamol:<br><4 yrs, 0.25mL;<br>4-8 yrs, 0.5mL.	7 days/10-15 min/time, 2 times/day	Standard care + Budesonide (0.5 mg, every 10-15 mins, twice/day)	Any adverse events;<br>Clinical response
Liu 2012	China	Acute asthma	62	7.4	hospital (inpatient)	NR	Salbutamol:<br>4-8 yrs, 2.5mg;<br>8-12 yrs, 3.75mg;<br>>12 yrs, 5mg.<br>IB:<br><6 yrs, 125mg;<br>≥6 yrs, 250mg.	Salbutamol:<br>4-8 yrs, 2.5mg;<br>8-12 yrs, 3.75mg;<br>>12 yrs, 5mg.	5 days/2 times/day	Standard care + budesonide	Lengths of hospital stay;<br>Percent change in FEV_1
Liu 2016	China	Asthma	60	7.34	hospital (inpatient and/or outpatient)	NR	Salbutamol: 0.1-0.2 mg<br>IB: 1.25ml (3-6 yrs)<br>2.5ml (6-15 yrs)	Salbutamol: 0.1-0.2 mg<br>Ambroxol: 2-3 mg/kg	7days,Once in 4 hours when necessary, no more than 8 times daily.	NR	Percent change in FEV_1;<br>Specific adverse events

							Ambroxol: 2-3 mg/kg				
Luo 2004	China	Asthma	114	7.5	hospital (inpatient)	NR	2mL (with added saline) Salbutamol: <4 yrs, 0.25mL; 4-8 yrs, 0.5mL; 8-12 yrs, 0.75mL; > 12 yrs, 1.0mL. IB: <4 yrs, 0.5mL; 4-8 yrs, 1.0mL; 8-12 yrs, 1.5mL; > 12 yrs, 2.0mL.	2mL (with added saline) Salbutamol: <4 yrs, 0.25mL; 4-8 yrs, 0.5mL; 8-12 yrs, 0.75mL; > 12 yrs, 1.0mL.	10-20 min/time, 2 times/day	Standard care + Budesonide (1 mL)	Length of hospital stay; Change in clinical score; Specific adverse events
Luo 2014	China	Asthma	156	7.4	hospital (inpatient)	1.2	IB solution: 0.5 ml (IB 0.125 ml) Salbutamol: 0.02 ml/kg 0.9 % Nacl was added to each treatment dose to achieve a total volume of 2 mL. 5 ml/time	Salbutamol: 0.02 ml/kg 0.9% Nacl was added to each treatment dose to achieve a total volume of 2 mL. 5 ml/time	one month, Twice daily	Standard care, such as prevention of pulmonary infection or relieve symptoms with aminophylline.	Clinical response; Percent change in FEV_1; Change in clinical score
Memon 2016	Pakistan	Asthma	200	9.2	hospital (outpatient)	NR	IB: 250 µg/kg/dose Salbutamol: 0.03 ml/kg/dose	Salbutamol: 0.03 ml/kg/dose	45 minutes/every 15 minutes	NR	Change in clinical score

Ni 2003	China	Bronchial asthma	141	NR	hospital (non-specific)	NR	2mL (with added 0.9%NaCl) Salbutamol: <4 yrs, 0.25mL; 4-8 yrs, 0.5mL; 8-12 yrs, 0.75mL. IB: <4 yrs, 0.25mL; 4-8 yrs, 0.5mL; 8-12 yrs, 0.75mL.	2mL (with added 0.9%NaCl): <4 yrs, 0.25mL; 4-8 yrs, 0.5mL; 8-12 yrs, 0.75mL.	3 days/2-4 time/day	Standard care	None
Nibhanipudi 2009	the United States	Asthma	60	11	hospital (outpatient)	NR	Albuterol: 2.5 mg Ipratropium: 0.5 mg	Albuterol: 2.5 mg	NR	NR	None
Nong 2011	China	Asthma	84	1.7	hospital (non-specific)	0.14	Salbutamol: 0.3 mg/kg; IB: 0.06 mg/kg	Salbutamol: 0.3 mg/kg	5 days, 1 time/day	Standard care	Clinical response
Pang 2014	China	Asthma	98	5.8	hospital (non-specific)	NR	adjusted according to patient age	According to patient age	3 times/day, 15min/time	Standard care	Clinical response
Qureshi 1997	the United States	Asthma	90	12	hospital (outpatient)	NR	Albuterol: 0.15 mg/kg, maximum 5 mg IB: 500 µg	Albuterol: 0.15 mg/kg, maximum 5 mg	90 minutes/Albuterol: every 30 minutes for 3 doses, continued every 30 minutes as needed; Ipratropium	Prednisone or prednisolone 2 mg/kg, maximum dose 60 mg together with the second dose of nebulized albuterol.	Hospital admission; Any adverse events; Change in % predicted FEV_1; Specific adverse events; Relapse rate

									bromide: the first and the third dose.		
Qureshi 1998	the United States	Asthma	480	8.4	hospital (outpatient)	NR	Albuterol: 2.5 mg for weighing<20kg; 5 mg for weighing≥20kg IB: 500 µg	Albuterol: 2.5 mg for weighing<20kg; 5 mg for weighing≥20kg Placebo: Normal saline: 2.5 ml	60 minutes/Albuterol: 3 doses, every 20 minutes; Ipratropium bromide: second and third dose	Corticosteroid (2 mg of prednisone or prednisolone per kilogram of body weight, to a maximal dose of 60 mg) the second dose	Hospital admission; Clinical response; Oxygen saturation; Relapse rate
Rayner 1987	the United Kingdom	Asthma	40	6.5	hospital (non-specific)	NR	Salbutamol: 2.5 mg, or 5 mg for those over 6 years old Ipratropium: 250 µg in 2 ml physiological saline	Salbutamol:2.5 mg, or 5 mg for those over 6 years old Placebo: saline: 3 ml	Salbutamol: on admission and four hourly thereafter; Ipratropium: 30 minutes after first dose of salbutamol and eight hourly thereafter.	NR	Length of hospital stay; Change in clinical score; Need for systemic corticosteroids
Reisman 1988	Canada	Asthma	25	10.1	hospital (outpatient)	NR	Salbutamol: 150 µg/kg, maximum 5 mg, followed by six doses of 50 µg/kg, manimum 1.7mg IB: 250 µg	Salbutamol: 150 µg/kg, maximum 5 mg, followed by six doses of 50 µg/kg, manimum 1.7mg Placebo: saline: 1 ml	120 minutes/Salbutamol: every 20 minutes; Ipratropium bromide: time 0, 40 minutes and 80 minutes	NR	Hospital admission; Change in % predicted FEV_1; Specific adverse events

Sienra Monge 2000	Mexico	Asthma	60	NR	NR	NR	IB: 120 mcg; Salbutamol: 200 mcg	Salbutamol 200 mcg	q10 minutes x 3	NR	Hospital admission; % change in FEV_1
Schuh 1995	Canada	Asthma	121	9.3	hospital (outpatient)	27.6 hours	Albuterol: 0.15 mg/kg per dose IB: 250 µg/dose (gruop 1: 3 doses; group 2: 1 dose)	Albuterol: 0.15 mg/kg per dose Placebo: 1 ml	60 minutes/every 20 minutes	Neither corticosteroids nor other bronchodilator s	Hospital admission; Any adverse events; Clinical response; Change in % predicted FEV_1; Change in clinical score; Oxygen saturation; Need for repeated bronchodilator treatments; Relapse rate
Sha 2011	China	asthma	106	NR	NR	NR	Salbutamol: 0.25mL; IB: 0.5 mL	0.25mL	2-3 time/day	Budesonide (0.5mL) and water for injection (1mL)	Length of hospital stay; Clinical response
Sharma 2004	India	Asthma	50	10.5	hospital (outpatient)	NR	Salbutamol:150 µg/kg/dose, maximum of 5 mg IB: 250 µgm/dose	Salbutamol: 150 µg/kg/dose, maximum of 5 mg	60 minutes/every 20 minutes	NR	Hospital admission; Clinical response; Change in clinical score; Specific adverse events; Relapse rate

Storr 1986	the United Kingdom	Asthma	138	5	hospital (non-specific)	NR	Salbutamol: 5 mg Ipratropium: 0.25 mg	Salbutamol: 5 mg	NR	Steroids: not responding satisfactorily to nebulised treatment; Aminophylline: children in severe respiratory distress.	Need for systemic corticosteroids
Wang 2019	China	Bronchial asthma	130	3.7	hospital (non-specific)	2.37 days	Salbutamol: 100 µg; IB: 0.4-1.0 mL	100 µg	28 days, 3-4 times/day	Diprophylline Injection: 0.25g in 5% Glucose injection, 1 time/day.	Percent change in FEV_1
Wang 2019a	China	Bronchial asthma	66	6.44	NR	NR	IB: 2 ml; Salbutamol: 2 ml; The above drugs should be adjusted appropriately according to weight and condition.	Salbutamol:2 ml; The above drugs should be adjusted appropriately according to weight and condition.	Twice daily for one week	none	Length of hospital stay; Clinical response
Watanasomsiri 2006	Thailand	Asthma	74	7	hospital (outpatient)	3.2	Salbutamol: 1.2 mg for weight<10kg; 2.5 mg for weight≥10kg IB: 250 µg	Salbutamol: 1.2 mg for weight<10kg; 2.5 mg for weight≥10kg	60 minutes/every 20 minutes, additional doses of salbutamol were given every 30	Oral steroid 0.5 mg/kg	Hospital admission; Any adverse events; Change in clinical score; Specific

									minutes as needed.		adverse events; Relapse rate
Watson 1994	Canada	Asthma	20	12.5	hospital (non-specific)	NR	Albuterol: 0.1 mg/kg, maximum 5 mg IB: 250 µg if weighed<40kg; 500 µg if weighted>40kg	Albuterol: 0.1 mg/kg, maximum 5 mg	At start	NR	Specific adverse events
Wu 2009	China	Asthma	112	3.1	hospital (outpatient)	NR	3mL (with added 0.9% NaCl) Salbutamol: 0.3-0.5 ml; IB: 0.5-1 ml	3mL (with added 0.9% NaCl) Salbutamol: 0.3-0.5 ml	30 minutes and 3 days/2 times/day	Standard care + Budesonide (1 mL)	Clinical response
Wyatt 2015	Australia	Asthma	418	Median 4.3	hospital (outpatient)	NR	Salbutamol: 600 µg for 2-5 years; 1200 µg for 6-15 years IB: 84 µg for 2-5 years; 168 µg for 6-15 years	Salbutamol: 600 µg for 2-5 years; 1200 µg for 6-15 years	60 minutes/every 20 minutes	Prednisolone 1 mg/kg to a maximum of 50 mg	Hospital admission; Any adverse events; Specific adverse events
Yi 2015	China	Asthma	84	6.7	hospital (non-specific)	2.8	NR	NR	5 days, 2 times/day	Standard care	Clinical response; Specific adverse events
Yin 2014	China	Bronchial asthma	61	5.7	hospital (non-specific)	NR	Salbutamol: 200-500µg; IB: 0.5-1 potion.	200-500µg	NR	Standard care + Azithromycin sequential therapy	Clinical response
Yin 2018	China	Asthma	76	7	hospital (non-specific)	7.23	Salbutamol: 0.1-0.2 mg IB: 1.25 ml (3-6	Salbutamol: 0.1-0.2 mg Aminophylline:	7days, Once in 4 hours when necessary, no	NR	Any adverse events; Percent change

							yrs)<br>2.5 ml (6-15 yrs)<br>Aminophylline: 2-3 mg/kg, dilute with glucose solution (5 %, 500 mL) by intravenous infusion	2-3 mg/kg, dilute with glucose solution (5 %, 500 mL) by intravenous infusion	more than 8 times daily.		in FEV_1; Specific adverse events
Yuksel 2001	Turkey	Wheezy	31	0.85	NR	NR	Salbutamol: 0.075 mg/kg per time;<br>IB: 0.25 mg	initial dose of 0.15 mg/kg (max. 3 mg), plus two consecutive doses of 0.15 mg/kg.	60 minutes/every 20 minutes	NR	Change in respiratory resistance; Oxygen saturation
Zhang 2012	China	Bronchial asthma	100	6.1	hospital (outpatient)	NR	Salbutamol: 0.03 ml/kg;<br>IB: 1 mL	Salbutamol: 0.03 ml/kg	3-7 days, twice/day	Standard care	Clinical response; Specific adverse events
Zhu 2019	China	Asthma	90	10.2	hospital (non-specific)	NR	IB: 250 ug<br>Salbutamol: oral<br>Aminophylline: intravenous drip	Salbutamol: oral<br>Aminophylline: intravenous drip	14 days, twice/day	1. Patients recieved regular treatment such as oxygen inhalation or antibiotics against infection or antiviral therapy.	Clinical response; Percent change in FEV_1

										2. Symptomatic treatment was given to correct acid-base and electrolyte balance as well as nutritional support.	
Zorc 1999	the United States	Asthma	427	7.6	hospital (outpatient)	NR	Nebulized albuterol: 2.5 mg in 3 mL saline or 5.0 mg in 6 mL based on weight < or ≥ 30 kg;<br>IB: 250 µg	Nebulized albuterol: 2.5 mg or 5.0 mg based on weight < or ≥ 30 kg;<br>0.9% saline: 1 ml/dose.	60 minutes/every 20 minutes	Predinisone	Length of ED stay; Relapse rate
Benito Femandeza 2000	Spain	Asthma	102	5.7	NR	NR	IB: 250 mcg*2 ; Salbutamol: 2 dose of 0.2 mg/kg, 6 mg max, 30 minutes apart	Salbutamol: 2 dose of 0.2 mg/kg, 6 mg max, 30 minutes apart	2 weeks	Methylprednisolone 1 mg/kg, 40 mg max (oral administration)	Hospital admission; Change in clinical score; Oxygen saturation
BI [pers comm]	Philippines	Asthma	500	NR	NR	NR	IB: 500 mcg; Salbutamol: 2.5 mg	Salbutamol: 2.5 mg UDV	1 UDV every 20 minutes for 3 doses	NR	Hospital admission; Oxygen saturation
Peterson 1996	Canada	Asthma	163	NR	NR	NR	IB: 250 mcg q45 minutes/2 doses;<br>Salbutamol: 3 mg q45	Salbutamol 3 mg q45 minutes/2 doses	120 minutes	Additional inhalation of salbutamol + placebo/ipratropium at 90 minutes if	Change in % predicted FEV_1; Oxygen saturation; Need for repeated

							minutes/2 doses			FEV_1< 85% of predicted; Systemic corticosteroids: at discretion of treating physician; Other medications: documented	bronchodilator treatments; Specific adverse events; Need for systemic corticosteroids
