# Supplementary material for: Combination of ipratropium bromide and salbutamol in children and adolescents with asthma: A meta-analysis
Source: PLoS One. 2021 Feb 23;16(2):e0237620. doi: 10.1371/journal.pone.0237620 (PMC7901745; doi:10.1371/journal.pone.0237620)
Supplement: S6 Appendix — (PDF) [file pone.0237620.s006.pdf]

Appendix 6 Quality of included studies (part 1 of 2)

Study ID	Randomisation	Support for judgement	Allocation concealment	Support for judgement	Blinding of participants and personnel	Support for judgement
Anthracopoulos 2005	Unclear risk	Quote: "The study design was a randomized, double-blind trial, with two study groups." (p.274)	Unclear risk	Comments: The author did not describe the information.	Low risk	Quote: "Treatments were prepared by an independent party based on the patient s weight, according to a preset code. Nebulization treatments administered were identical regarding volume and color. Both patient and investigator were blinded to group assignment." (p.274)
Beck 1985	Unclear risk	Quote: "Patients were entered in a double-blind random manner into one of two treatment groups." (p.605)	Unclear risk	Comments: The author did not describe the information.	Unclear risk	Quote: "Patients were entered in a double-blind random manner into one of two treatment groups....This solution was drawn from coded vials, the code for which was held by an independent person in the pharmacy department. " (p.605)
Calvo 1998	High risk	Consecutive assignment	Low risk	Number-coded solutions supplied by pharmacy.	Low risk	Identical placebo
Chakraborti 2006	Low risk	Quote: "Block randomization was done by computer-generated blocks of 6 each." (p.980)	Low risk	Quote: "For blinding, similar looking MDIs were labeled A, B, C, D, E, F; 3 of which contained ipratropium and 3 contained placebos. As per random numbers patients were given drugs from these MDIs. A person not involved in the study evaluation did the labeling. The analysis was performed by investigator (RMP), unaware of the labeling of the two groups as drug or control." (p.980)	Unclear risk	Quote: "All patients were administered 4 actuations of salbutamol through similar looking MDI and spacer. Then 4 actuations of either drug (ipratropium) or placebo were given through MDI and spacer as determined by block randomization (A, B, C, D, E, or F)." (p.980)
Chen 2010	Unclear	Quote:"... randomly divided into two groups" (P555)	Unclear	The author did not describe the allocation concealment.	Unclear	The author did not describe the blinding of participants and personal.

Coskun 2001	Low risk	Quote: "The patients were divided into two groups by using a simple randomization procedure. In this procedure, they had been numbered and enrolled into group 1 and group 2 sequentually." (p.632)	Unclear risk	Comments: The author did not describe the information.	Low risk	Quote: "patient and physicians were masked to the treatment strategy." (p.632)
Craven 2001	Low risk	randomised using random number table method. Quote: …a table of random numbers was used to assign children…	Low risk	randomised using random number table method. Quote: …a table of random numbers was used to assign children…	Unclear risk	double blinded. Quote: …children by simple randomisation in blocks of 10 to the intervention or placebo group in double-blind fashion.
Dai 2000	Unclear	Comments: the author described an random component, but no more detail about method. Quote:"The patients were randomly and blindly divided into 2 groups."(P12)	Unclear	The author did not describe the allocation concealment.	Unclear	Comments: the author did not detailedly describe the method about blinding. Quote:"The patients were randomly and blindly divided into 2 groups."(P12)
Ding 2010	Unclear risk	randomised. Quote: 92 asthma patients were randomly divided into three groups.	Unclear risk	no information provided	Unclear risk	no information provided
Ducbarme 1998	Low risk	randomised using a simple randomisation procedure. Quote: patients were assigned to treatment groups according to a simple randomisation procedure from a computer-generated list of random numbers with a fixed seed.	Low risk	randomised using a simple randomisation procedure. Quote: patients were assigned to treatment groups according to a simple randomisation procedure from a computer-generated list of random numbers with a fixed seed.	Low risk	double blinded. Quote: we conducted a blinded, randomised…in which the patient and parents, treating physician, and assessor were masked to the treatment strategy.
Guo 2015	Unclear risk	randomised, but no details provided. Quote: randomly divided into experiment group and control group.	Unclear risk	no information provided	Unclear risk	no information provided
He 2011	Unclear	Comments: the author described a random component, but no more detail about random methods.	Unclear	The author did not describe the allocation concealment.	Unclear	The author did not describe the blinding of participants and personal.

		Quote:"...randomly divided into experiment group and control group"(P1445)				
Iramain 2011	Low risk	Quote: "Patients were randomized using a computer-generated random sequence." (p.299)	Unclear risk	Comments: The author did not describe the information.	Low risk	Quote: "The hospital pharmacy department prepared two types of numbered plastic bottles. The two solutions had the same smell, color, and fluid level in order to prevent differentiation. Neither the investigators nor the patients knew which solution the bottles contained. The bottles were kept in the emergency department ready for immediate use." (p.299)
Ji 2003	Unclear risk	randomised, but no details provided. Quote: randomly divided into experiment group and control group.	Unclear risk	no information provided	Unclear risk	no information provided
Kong 2003	Unclear risk	randomised, but no details provided. Quote: randomly divided into 2 groups…	Unclear risk	no information provided	Unclear risk	no information provided
Kumaratne 2003	Low risk	randomised, using random number table method. Quote: patients with an asthma score of 6 or greater were randomly assigned to the control group or the ipratropium group using random tables.	Low risk	randomised, using random number table method. Quote: patients with an asthma score of 6 or greater were randomly assigned to the control group or the ipratropium group using random tables.	Unclear risk	no information provided
Li 2000	Unclear	Comments: the author described an random component, but no more detail about mothed. Quote:"The study design was a randomized, double-blind trial, with two study groups".(P25)	Unclear	The author did not describe the allocation concealment.	Unclear	The author did not describe the blinding of participants and personal.
Li 2011	Unclear risk	randomised, but no details provided. Quote: randomly divided into 2 groups.	Unclear risk	no information provided	Unclear risk	no information provided

Liang 2018	Low risk	randomised using random number table method. Quote: randomly divided into experiment group (n=40) and control group (n=40) according to random number table…	Low risk	randomised using random number table method. Quote: randomly divided into experiment group (n=40) and control group (n=40) according to random number table…	Unclear risk	no information provided
Liao 2019	Unclear risk	randomised, but no details provided. Quote: randomly divided into experiment group and control group.	Unclear risk	no information provided	Unclear risk	no information provided
Lin 2010	Unclear risk	randomised, but no details provided. Quote: randomly divided into 2 groups.	Unclear risk	no information provided	Unclear risk	no information provided
Liu 2012	Unclear risk	randomised, but no details provided. Quote: 62 acute asthma patients were randomly divided into 2 groups.	Unclear risk	no information provided	Unclear risk	no information provided
Liu 2016	Low risk	Quote:" ...divided into experiment group and control group according to random number table"(P150)	Unclear	The author did not describe the allocation concealment.	Unclear	The author did not describe the blinding of participants and personal.
Luo 2004	Unclear risk	randomised, but no details provided. Quote: randomly divided into 2 groups.	Unclear risk	no information provided	Unclear risk	no information provided
Luo 2014	Unclear	Comments:the author described an random component, but no more detail about method. Quote:"... randomly divided into two groups."(P97)	Unclear	NR	Unclear	NR
Memon 2016	Unclear risk	Quote: "The randomised controlled trial (RCT) was conducted....." (p.244)	Unclear risk	Comments: The author did not describe the information.	Unclear risk	Comments: The author did not describe the information.
Ni 2003	Unclear risk	randomised, but no details provided. Quote: randomly divided into 3 groups…	Unclear risk	no information provided	Unclear risk	no information provided

Nibhanipudi 2009	Unclear risk	Quote: "Patients were randomized into four groups: Groups A, B, C, and D." (p.447)	Unclear risk	Comments: The author did not describe the information.	High risk	Quote: "The study is an ... non-blinded study." (p.447)
Nong 2011	Low risk	randomised, using random number table method. Quote: patients were randomly assigned to experiment group and control group with 42 patients in each group using random tables.	Low risk	randomised, using random number table method. Quote: patients were randomly assigned to experiment group and control group with 42 patients in each group using random tables.	Unclear risk	no information provided
Pang 2014	Unclear risk	randomised, but no details provided. Quote: randomly divided into 2 groups.	Unclear risk	no information provided	Unclear risk	no information provided
Qureshi 1997	Low risk	Quote: "Patients were assigned with the use of a table of random numbers to one of two treatment groups." (p.206)	Unclear risk	Comments: The author did not describe the information.	Low risk	Quote: "The pharmacy department provided numbered plastic bags that contained either two vials of normal saline or two vials of ipratropium bromide (500 µg per dose). The two agents were identical in appearance and aroma, and a supply of the study drug was maintained in the ED to avoid any time delay between study enrollment and treatment. Investigators and patients were blinded to both group assignment and vial contents." (p.206)
Qureshi 1998	Low risk	Quote: "Pharmacy staff used a table of random numbers to assign children by block randomization to treatment and control groups." (p.1031)	Low risk	Quote: "Pharmacy staff used a table of random numbers to assign children.....They also provided numbered plastic bags." (p.1031)	Low risk	Quote: "The contents of the two types of vials were identical in appearance and aroma. Both the investigators and the patients were unaware of the group assignments and vial contents." (p.1031)
Rayner 1987	Unclear risk	Quote: "...were randomly allocated to two groups in a double blind trial." (p.840)	Unclear risk	Comments: The author did not describe the information.	Unclear risk	Quote: "...were randomly allocated to two groups in a double blind trial." (p.840)
Reisman 1988	Unclear risk	Quote: "Subjects were entered in a double-blind, randomized	Unclear risk	Comments: The author did not describe the information.	Unclear risk	Quote: "Subjects were entered in a double-blind, randomized

		manner into one of two treatment groups." (p.17)				manner into one of two treatment groups." (p.17)
Sienra Monge 2000	Unclear risk	Information not available	Unclear risk	Information not available	Low risk	Double-blind
Schuh 1995	Low risk	Quote: "The randomization code was generated by the hospital pharmacy department from a standard table of random numbers in blocks, so that after each nine patients there would be three subjects allocated to each group." (p.640)	Unclear risk	Comments: The author did not describe the information.	Low risk	Quote: "The pharmacy department provided three vials that contained either ipratropium or placebo (0.9% saline solution) labeled Nos. 1, 2, and 3. Albuterol-ipratropium and albuterol-saline mixtures are indistinguishable in both the liquid and the nebulized states. The patient, the research nurse, and the investigator were masked to the group assignment." (p.640)
Sha 2011	Unclear risk	randomised, but no details provided. Quote: randomly divided into 2 groups.	Unclear risk	no information provided	Unclear risk	no information provided
Sharma 2004	Unclear risk	Quote: "This was a prospective randomized study and children were divided into two groups of twenty five children each." (p.122)	Unclear risk	Comments: The author did not describe the information.	High risk	Quote: "The study was not blind so as not to interfere with emergency treatment of patients." (p.122)
Storr 1986	Unclear risk	Quote: "The children were randomly allocated to a salbutamol or a combined treatment group." (p.602)	Unclear risk	Comments: The author did not describe the information.	Unclear risk	Quote: "Nebuliser solutions were of equal tonicity and were administered double blind." (p.602)
Wang 2019	Low risk	randomised using random number table method. Quote: randomly divided into experiment group (n=65) and control group (n=65) according to random number table…	Low risk	randomised using random number table method. Quote: randomly divided into experiment group (n=65) and control group (n=65) according to random number table…	Unclear risk	no information provided
Wang 2019a	Low risk	Quote:" ...divided into 2 groups according to random number table." (P53)	Unclear	The author did not describe the allocation concealment.	Unclear	The author did not describe the blinding of participants and personal.
Watanasomsiri 2006	Unclear risk	Quote: "A dispensing record was kept to document adherence to	Low risk	Quote: "The pharmacy supplied the solutions, and	Low risk	Quote: "The pharmacy supplied the solutions, and these solutions were drawn

		the randomization scheme." (p.702)		these solutions were drawn from vials by an independent nurse while the patients, parents, and physicians were unaware of the group assignments and vial contents. The code was broken at the end of the study." (p.702)		from vials by an independent nurse while the patients, parents, and physicians were unaware of the group assignments and vial contents. The code was broken at the end of the study." (p.702)
Watson 1994	Unclear risk	Quote: "ln a double-blind, randomized three-way crossover fashion......" (p.1439)	Unclear risk	Comments: The author did not describe the information.	Unclear risk	Quote: "ln a double-blind, randomized three-way crossover fashion......" (p.1439)
Wu 2009	Low risk	randomised using random number table method. Quote: randomly divided into 3 groups according to random number table…	Low risk	randomised using random number table method. Quote: randomly divided into 3 groups according to random number table…	Unclear risk	no information provided
Wyatt 2015	Low risk	Quote: "The subjects were randomised into two groups, using blocked (n = 6) computerised random number generation, and allocated in numerical order." (p.193)	Low risk	Quote: "The individual allocations were concealed in opaque envelopes that were opened by the ED nurse prior to initiating treatment." (p.193)	Low risk	Quote: "Nursing staff treated the patient as per these instructions. The doctor managing the patient was not present during the administration of treatment and thereby remained blinded to which group the patient was allocated to and which treatment they received. Additionally, to maintain the single blind, the exact treatment given was not documented in the patient record. A double-blind methodology was unable to be used, as placebo metered-dose inhalers (MDIs) are unlicensed for use in children." (p.193)
Yi 2015	Unclear risk	randomised, but no details provided. Quote: children with asthma was randomly divided into experiment group and control group with 42 in each group.	Unclear risk	no information provided	Unclear risk	no information provided

Yin 2014	Unclear risk	randomised, but no details provided. Quote: children with asthma that evoked by mycopiasma pneumoniae infection was randomly divided into group A (n=30) and group B (n=31).	Unclear risk	no information provided	Unclear risk	no information provided
Yin 2018	Unclear	Comments: the author described a random component, but no more detail about random methods. Quote:" ...randomly divided into two groups ." (P38)	Unclear	The author did not describe the allocation concealment.	Unclear	The author did not describe the blinding of participents and personal.
Yuksel 2001	Unclear risk	randomised. Quote: After physical examination, randomised selected infacts by assessor (nurse) were consecutively treated…	Low risk	Quote: patients were assigned to treatment groups according to a simple randomisation procedure. Subjects were selected consecutively and numbered as 1,2,3 etc.	Low risk	double blinded. Quote: …patients, parents and physicians were masked to the treatment strategy.
Zhang 2012	Unclear risk	randomised, but no details provided. Quote: children with asthma was randomly divided into experiment group and control group.	Unclear risk	no information provided	Unclear risk	no information provided
Zhu 2019	Low risk	Quote:" ...randomly divided into two groups by random number method" (P626)	Unclear	The author did not describe the allocation concealment.	Unclear	The author did not describe the blinding of participents and personal.
Zorc 1999	Low risk	Quote: "Before the study, vials were block randomized in groups of eight by a standard computerized method in the Investigational Pharmacy." (p.749)	Low risk	Quote: "After consent was obtained, each patient was assigned a study vial that had been prepared in advance by the pharmacy." (p.749)	Low risk	Quote: "Each numbered amber vial contained either normal saline or a solution of ipratropium bromide in a concentration of 250 μg/ml normal saline. Both solutions are clear, odorless, and indistinguishable in the liquid and nebulized states......Investigators, physicians, nurses, and patients were blind to the randomization code." (p.749)

Benito Femandeza 2000	Low risk	Block randomisation	Low risk	The department of pharmacy provided identical packages.	Low risk	Both solutions had an identical smell and physical appearance.
BI [pers comm]	Unclear risk	Described as randomised; other information not available.	Unclear risk	Information not available.	Unclear risk	Described as double blind; other information not available.
Peterson 1996	Low risk	Computer-generated random numbers.	Low risk	Nuber-coded solutions supplied by the pharmacy.	Low risk	Identical placebo

Appendix 6 Quality of included studies (part 2 of 2)

**Study ID**	**Blinding of outcome assessor**	**Support for judgement**	**Incomplete data**	**Support for judgement (drop-out)**	**Selective reporting**	**Support for judgement**	**Other bias**	**Support for judgement (study funding)**
Anthracopoulos 2005	Low risk	Quote: "Hospital admission was judged by the emergency room pediatrician (not a study group member)." (p.277)	Low risk	Comments: All the patients were included in the result analysis.	Low risk	All outcomes stated in methods were reported.	Low risk	None obvious.
Beck 1985	Unclear risk	Comments: The author did not describe the information.	Low risk	Comments: All the patients were included in the result analysis.	Low risk	All outcomes stated in methods were reported.	High risk	Quote: "Boehringer Ingelheim (Canada) for supplied the ipratropium bromide for this study." (p.607)
Calvo 1998	Low risk	Identical placebo	Low risk	All participants completed.	Low risk	None noted.	Low risk	None identified.
Chakraborti 2006	Unclear risk	Comments: The author did not describe the information.	Low risk	Comments: All the patients were included in the result analysis.	Low risk	All outcomes stated in methods were reported.	Low risk	None obvious.
Chen 2010	Unclear	The author did not describe the blinding of outcome assessor.	Low risk	No missing outcome data.	Low risk	All outcomes stated in methods were reported.	Low risk	None obvious.
Coskun 2001	Unclear risk	Comments: The author did not describe the information.	Low risk	Comments: All the patients were included in the result analysis.	Low risk	All outcomes stated in methods were reported.	Low risk	None obvious.

Craven 2001	Unclear risk	no information provided	Low risk	no drop-out reported	Low risk	All outcomes stated in methods were reported.	Unclear risk	no funding information, but the drug was supplied by RBC centre.
Dai 2000	Unclear	Comments: the author did not detailedly describe the method about blinding. Quote:"The patients were randomly and blindly divided into 2 groups."(P12)	Unclear	No missing outcome data.	Low risk	All outcomes stated in methods were reported.	Low risk	None obvious.
Ding 2010	Unclear risk	no information provided	Low risk	no drop-out reported	Low risk	All outcomes stated in methods were reported.	Low risk	no funding information, assume to be no funding.
Ducbarme 1998	Low risk	double blinded. Quote: we conducted a blinded, randomised…in which the patient and parents, treating physician, and assessor were masked to the treatment strategy.	Low risk	no drop-out reported	Low risk	All outcomes stated in methods were reported.	Unclear risk	supported by the medical research council of Canada.
Guo 2015	Unclear risk	no information provided	Low risk	no drop-out reported	Low risk	All outcomes stated in methods were reported.	Low risk	no funding information, assume to be no funding.
He 2011	Unclear	The author did not describe the blinding of outcome assessor.	Low risk	No missing outcome data.	Low risk	All defined outcomes were reported	Low risk	None obvious.
Iramain 2011	Low risk	Quote: "The decision to discharge or admit patients and the treatment after discharge was made by pediatricians who did not know the	Low risk	Quote: "nine were excluded from the enrolled 106 patients......" (p.300)	Low risk	All outcomes stated in methods were reported.	Unclear risk	Quote: "Part of the study has been financed by grant CYTED 209RT0377 from the Spanish Science and Technology Program for Development." (p.302)

		treatment used." (p.299)						
Ji 2003	Unclear risk	no information provided	Low risk	no drop-out reported	Low risk	All outcomes stated in methods were reported.	Low risk	no funding information, assume to be no funding.
Kong 2003	Unclear risk	no information provided	Low risk	no drop-out reported	Low risk	All outcomes stated in methods were reported.	Low risk	no funding information, assume to be no funding.
Kumaratne 2003	Unclear risk	no information provided	Low risk	no drop-out reported	Low risk	All outcomes stated in methods were reported.	Low risk	no funding information, assume to be no funding.
Li 2000	Unclear	The author did not describe the blinding of outcome assessor.	Unclear	No missing outcome data.	Low risk	All outcomes stated in methods were reported.	Low risk	None obvious.
Li 2011	Unclear risk	no information provided	Low risk	no drop-out reported	Low risk	All outcomes stated in methods were reported.	Low risk	no funding information, assume to be no funding.
Liang 2018	Unclear risk	no information provided	Low risk	no drop-out reported	Low risk	All outcomes stated in methods were reported.	Low risk	no funding information, assume to be no funding.
Liao 2019	Unclear risk	no information provided	Low risk	two drop-out with reason reported (less than 10%). Quote: two patients did not complete the whole treatment.	Low risk	All outcomes stated in methods were reported.	Low risk	no funding information, assume to be no funding.
Lin 2010	Unclear risk	no information provided	Low risk	no drop-out reported	Low risk	All outcomes stated in methods were reported.	Low risk	no funding information, assume to be no funding.
Liu 2012	Unclear risk	no information provided	Low risk	no drop-out reported	Low risk	All outcomes stated in methods were reported.	Low risk	no funding information, assume to be no funding.
Liu 2016	Unclear	The author did not descibe the blinding of outcome assessor.	Low risk	No missing outcome data.	Low risk	All defined outcomes were reported.	Low risk	None obvious

Luo 2004	Unclear risk	no information provided	Low risk	no drop-out reported	Low risk	All outcomes stated in methods were reported.	Low risk	no funding information, assume to be no funding.
Luo 2014	Unclear	NR	Low risk	No missing outcome data.	Low risk	All outcomes stated in methods were reported.	Low risk	None obvious.
Memon 2016	Unclear risk	Comments: The author did not describe the information.	Low risk	Comments: All the patients were included in the result analysis.	High risk	Quote: "Response to treatment was assessed after 15 minutes of the last dose and a change in severity category (improvement) from baseline to lower category was taken as improvement." (p.244) Comments: The number of change of category were not reported.	Low risk	Quote: "Funded by National Institute of Child Health (NICH), Karachi, Pakistan." (p.243)
Ni 2003	Unclear risk	no information provided	Low risk	no drop-out reported	Low risk	All outcomes stated in methods were reported.	Low risk	no funding information, assume to be no funding.
Nibhanipudi 2009	Unclear risk	Comments: The author did not describe the information.	Low risk	Comments: All the patients were included in the result analysis.	Low risk	All outcomes stated in methods were reported.	Low risk	None obvious.
Nong 2011	Unclear risk	no information provided	Low risk	no drop-out reported	Low risk	All outcomes stated in methods were reported.	Low risk	no funding information, assume to be no funding.
Pang 2014	Unclear risk	no information provided	Low risk	no drop-out reported	Low risk	All outcomes stated in methods were reported.	Low risk	no funding information, assume to be no funding.
Qureshi 1997	Unclear risk	Comments: The author did not describe the information.	Low risk	Comments: All the patients were included in the result analysis.	Low risk	All outcomes stated in methods were reported.	Low risk	None obvious.
Qureshi 1998	Low risk	Quote: "A decision to admit or discharge the child was made by the	Low risk	Quote: "In 46 children, wheezing resolved before the second dose	Low risk	All outcomes stated in methods were reported.	Low risk	Quote: "Supported by a grant from the Department of Pediatrics, Children's Hospital of the King's

		attending physician according to objective changes in the measurements of clinical and pulmonary function and according to the oxygen saturation (<94 percent or 94 percent in room air)." (p.1032)		of the study medication had been given...A total of 434 children (215 in the treatment group and 219 in the control group) completed the study." (p.1032)				Daughters, to purchase supplies." (p.1034)
Rayner 1987	Unclear risk	Comments: The author did not describe the information.	Unclear risk	Quote: "Two children failed to comply with the nebuliser treatment and one required intravenous treatment immediately after being given salbutamol, leaving 18 in the control group and 19 in the treatment group." (p.840)	Low risk	All outcomes stated in methods were reported.	Low risk	None obvious.
Reisman 1988	Low risk	Quote: "The decision whether to admit patients to hospital was at the discretion of the emergency room supervising physician, who was not an investigator in the study." (p.18)	Low risk	Quote: "The one subject not completing the study was too tired to cooperate with the spirometry." (p.18)	Low risk	All outcomes stated in methods were reported.	Low risk	None obvious.
Sienra Monge 2000	Low risk	Double-blind	Unclear risk	40 children referred to in the abstract but results are given for a study population of 30.	Unclear risk	Unable to ascertain this.	Low risk	None identified.

Schuh 1995	Low risk	Quote: "Children with respiratory distress at the end of the study were admitted. The decision to hospitalize the patients was made by the ED staff pediatrician not involved in the trial and unaware of the outcome measure changes (except for the oxygen saturation)." (P.640)	Low risk	Quote: "One parent changed his mind and demanded withdrawal before the start of the experimental therapy." (p.642)	Low risk	All outcomes stated in methods were reported.	High risk	Quote: "Supported by the Ontario Ministry of Health (Emergency Medical Services) and Boehringer Ingelheim." (p.639)
Sha 2011	Unclear risk	no information provided	Low risk	no drop-out reported	Low risk	All outcomes stated in methods were reported.	Low risk	no funding information, assume to be no funding.
Sharma 2004	High risk	Quote: "The study was not blind so as not to interfere with emergency treatment of patients." (p.122)	Low risk	Comments: All the patients were included in the result analysis.	Low risk	All outcomes stated in methods were reported.	Low risk	None obvious.
Storr 1986	Unclear risk	Comments: The author did not describe the information.	Low risk	Comments: All the patients were included in the result analysis.	Low risk	All outcomes stated in methods were reported.	Low risk	Quote: "JS was supported by a grant from the RAH Centenary Fund." (p.603)
Wang 2019	Unclear risk	no information provided	Low risk	no drop-out reported	Low risk	All outcomes stated in methods were reported.	Low risk	no funding information, assume to be no funding.
Wang 2019a	Unclear	The author did not describe the blinding of outcome assessor.	Low risk	No missing outcome data.	Low risk	All outcomes stated in methods were reported.	Low risk	None obvious.
Watanasomsiri 2006	Low risk	Quote: "Patients were evaluated and treated by a research physician under the supervision of the investigator.The	Low risk	Quote: "Two children were subsequently dropped from the study for technical reasons (errors in protocol), and	Low risk	All outcomes stated in methods were reported.	Low risk	Quote: "This study was supported by grant from the Faculty of Medicine, Thammasat University." (p.701)

		decision to hospitalize the patient was made by the research physician." (p.702)		1 child's guardian requested the child be withdrawn before the second dose of nebulized solution." (p.703)				
Watson 1994	Unclear risk	Comments: The author did not describe the information.	Low risk	Comments: All the patients were included in the result analysis.	Low risk	All outcomes stated in methods were reported.	High risk	Quote: "Supported in part by the Children's Hospital Research Foundation and Boerhinger Ingelheim, Canada." (p.1439)
Wu 2009	Unclear risk	no information provided	Low risk	no drop-out reported	Low risk	All outcomes stated in methods were reported.	Low risk	no funding information, assume to be no funding.
Wyatt 2015	Unclear risk	Comments: The author did not describe the information.	Unclear risk	Quote: "Of these, 436 were recruited, 418 randomised and 347 were suitable for analysis." (p.194) Figure 1 described the detail of incompleted reason.	Low risk	All outcomes stated in methods were reported.	Low risk	Quote: "This study has been internally funded by the Emergency Department, Princess Margaret Hospital for Children." (p.197)
Yi 2015	Unclear risk	no information provided	Low risk	no drop-out reported	Low risk	All outcomes stated in methods were reported.	Low risk	no funding information, assume to be no funding.
Yin 2014	Unclear risk	no information provided	Low risk	no drop-out reported	Low risk	All outcomes stated in methods were reported.	Low risk	no funding information, assume to be no funding.
Yin 2018	Unclear	The author did not descibe the blinding of outcome assessor.	Low risk	No missing outcome data.	Low risk	All outcomes stated in methods were reported.	Low risk	None obvious.
Yuksel 2001	Unclear risk	no information provided	Low risk	two drop-out with reason reported (less than 10%). Quote: one patient diagnosed as wheezy infant had foreign body aspiration	Low risk	All outcomes stated in methods were reported.	Low risk	no funding information, assume to be no funding.

				and admitted to the emergency department with wheezing and another had high dose of AB inhalation in NAB group were excluded from the study.				
Zhang 2012	Unclear risk	no information provided	Low risk	no drop-out reported	Low risk	All outcomes stated in methods were reported.	Low risk	no funding information, assume to be no funding.
Zhu 2019	Unclear	The author did not describe the blinding of outcome assessor.	Low risk	No missing outcome data.	High risk	Advers effects were not reported.	Low risk	None obvious.
Zorc 1999	Unclear risk	Comments: The author did not describe the information.	Low risk	Comments: All the patients were included in the result analysis.	Low risk	All outcomes stated in methods were reported.	Low risk	Quote: "This work was funded by the Thomas Wilson Sanitarium for Children of Baltimore City; Pediatric General Clinical Research Center, Johns Hopkins Hospital, Baltimore, Maryland; and Grant RR00052, Division of Research Resources, National Institutes of Health." (p.752)
Benito Femandeza 2000	Low risk	Both solutions had an identical smell and physical appearance.	Low risk	All participants accounted for in analysis (no withdrawals).	Unclear risk	Unable to ascertain	Low risk	None obvious.
BI [pers comm]	Unclear risk	Described as double blind; other information not available.	Low risk	2 withdrawals due to adverse events (1 in each group).	Unclear risk	Cannot ascertain this.	Unclear risk	Cannot ascertain this.
Peterson 1996	Unclear risk	Cannot be ascertained.	Unclear risk	Cannot be ascertained.	Unclear risk	Cannot be ascertained.	Unclear risk	Insufficient details provided (unpublished data).
