# Supplementary material for: Combination of ipratropium bromide and salbutamol in children and adolescents with asthma: A meta-analysis
Source: PLoS One. 2021 Feb 23;16(2):e0237620. doi: 10.1371/journal.pone.0237620 (PMC7901745; doi:10.1371/journal.pone.0237620)

Appendix 7. Meta-analysis forest plots of primary outcome – hospital admission (subgroup)

Subgroup of age

Study or Subgroup	Experimental Events	Experimental Total	Control Events	Control Total	Weight	Risk Ratio M-H, Random, 95% CI
**3.1.1 less than 6 yrs**						
Ducbarme 1998	4	49	8	43	100.0%	0.44 [0.14, 1.36]
**Subtotal (95% CI)**		**49**		**43**	**100.0%**	**0.44 [0.14, 1.36]**
Total events	4		8			
Heterogeneity: Not applicable						
Test for overall effect: Z = 1.43 (P = 0.15)						
**3.1.2 6 yrs old and over**						
Anthracopoulos 2005	4	10	1	10	26.8%	4.00 [0.54, 29.80]
Iramain 2011	6	33	14	32	47.9%	0.42 [0.18, 0.95]
Sharma 2004	1	25	4	25	25.3%	0.25 [0.03, 2.08]
**Subtotal (95% CI)**		**68**		**67**	**100.0%**	**0.67 [0.16, 2.80]**
Total events	11		19			
Heterogeneity: Tau² = 0.93; Chi² = 4.76, df = 2 (P = 0.09); I² = 58%						
Test for overall effect: Z = 0.55 (P = 0.58)						
**3.1.3 mixed**						
Benito Fernandez 2000	18	51	27	51	9.0%	0.67 [0.42, 1.05]
BI [pers comm]	19	246	23	254	6.3%	0.85 [0.48, 1.53]
Ducbarme 1998	23	155	24	143	7.3%	0.88 [0.52, 1.49]
Iramain 2011	9	49	21	48	5.0%	0.42 [0.21, 0.82]
Kumaratne 2003	0	24	1	26	0.3%	0.36 [0.02, 8.43]
Peterson 1996	19	82	25	81	7.6%	0.75 [0.45, 1.25]
Qureshi 1997	9	45	14	45	4.4%	0.64 [0.31, 1.33]
Qureshi 1998	59	215	80	219	15.2%	0.75 [0.57, 0.99]
Reisman 1988	2	11	3	13	1.1%	0.79 [0.16, 3.90]
Schuh 1995	32	79	19	41	9.8%	0.87 [0.57, 1.34]
Watanasomsiri 2006	2	38	3	33	0.9%	0.58 [0.10, 3.26]
Wyatt 2015	122	174	111	173	21.9%	1.09 [0.94, 1.27]
Zorc 1999	38	211	48	216	11.2%	0.81 [0.55, 1.19]
**Subtotal (95% CI)**		**1380**		**1343**	**100.0%**	**0.81 [0.68, 0.96]**
Total events	352		399			
Heterogeneity: Tau² = 0.03; Chi² = 18.92, df = 12 (P = 0.09); I² = 37%						
Test for overall effect: Z = 2.48 (P = 0.01)						

Test for subgroup differences: Chi² = 1.16, df = 2 (P = 0.56), I² = 0%

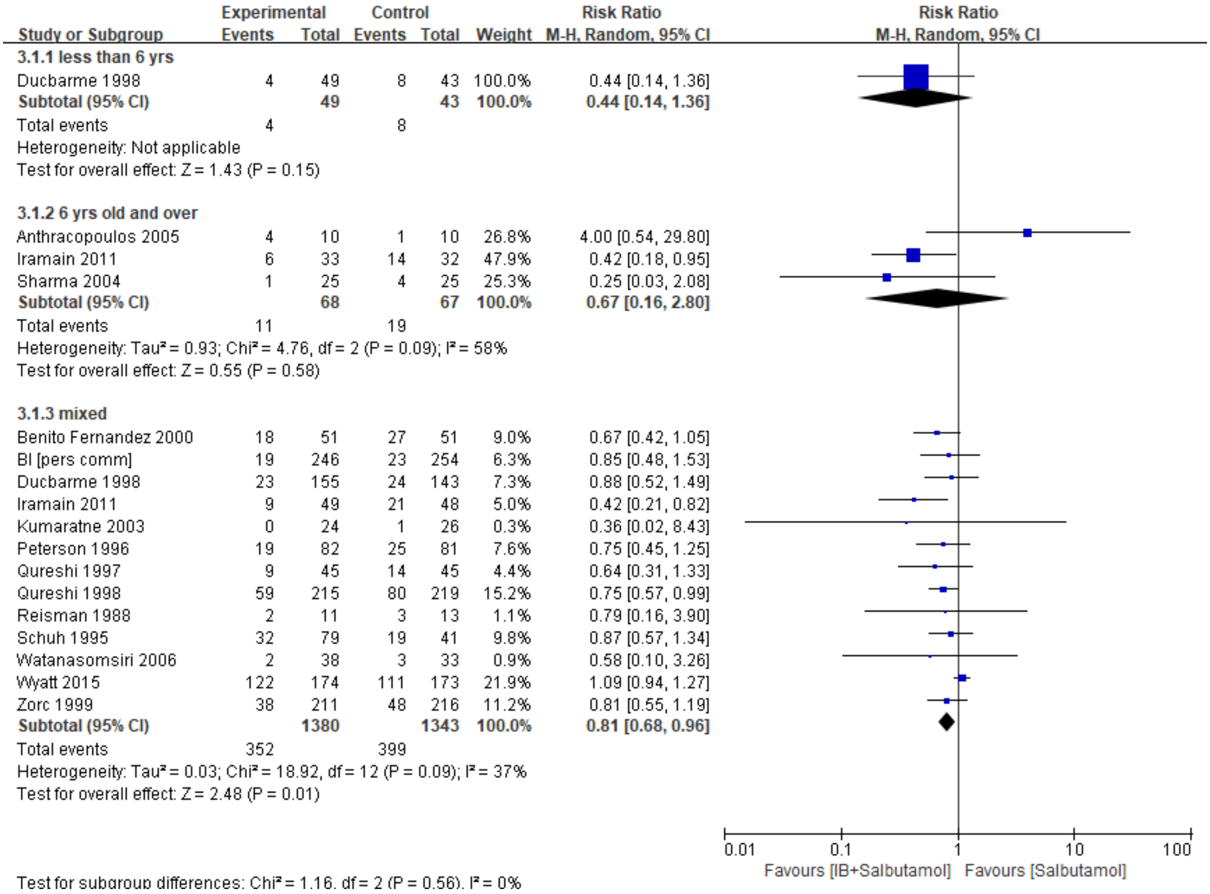

Subgroup of severity

Study or Subgroup	Experimental Events	Experimental Total	Control Events	Control Total	Weight	Risk Ratio M-H, Random, 95% CI
**4.1.1 mild**						
Zorc 1999	6	60	4	57	100.0%	1.43 [0.42, 4.79]
**Subtotal (95% CI)**		**60**		**57**	**100.0%**	**1.43 [0.42, 4.79]**
Total events	6		4			
Heterogeneity: Not applicable						
Test for overall effect: Z = 0.57 (P = 0.57)						
**4.1.2 moderate**						
Iramain 2011	1	15	2	17	0.5%	0.57 [0.06, 5.64]
Qureshi 1998	8	79	9	84	3.1%	0.95 [0.38, 2.33]
Wyatt 2015	122	174	111	173	87.8%	1.09 [0.94, 1.27]
Zorc 1999	18	98	25	96	8.6%	0.71 [0.41, 1.21]
**Subtotal (95% CI)**		**366**		**370**	**100.0%**	**1.04 [0.89, 1.22]**
Total events	149		147			
Heterogeneity: Tau² = 0.00; Chi² = 3.06, df = 3 (P = 0.38); I² = 2%						
Test for overall effect: Z = 0.54 (P = 0.59)						
**4.1.3 severe**						
Anthracopoulos 2005	4	10	1	10	0.9%	4.00 [0.54, 29.80]
Benito Fernandez 2000	18	51	27	51	16.2%	0.67 [0.42, 1.05]
BI [pers comm]	19	246	23	254	10.1%	0.85 [0.48, 1.53]
Iramain 2011	6	34	16	31	5.4%	0.34 [0.15, 0.76]
Qureshi 1998	51	136	71	135	40.7%	0.71 [0.54, 0.93]
Reisman 1988	2	11	3	13	1.4%	0.79 [0.16, 3.90]
Schuh 1995	32	79	19	41	18.3%	0.87 [0.57, 1.34]
Sharma 2004	1	25	4	25	0.8%	0.25 [0.03, 2.08]
Zorc 1999	7	22	12	29	6.2%	0.77 [0.36, 1.63]
**Subtotal (95% CI)**		**614**		**589**	**100.0%**	**0.73 [0.60, 0.88]**
Total events	140		176			
Heterogeneity: Tau² = 0.00; Chi² = 8.35, df = 8 (P = 0.40); I² = 4%						
Test for overall effect: Z = 3.33 (P = 0.0009)						
**4.1.4 mild to moderate**						
Ducbarme 1998	23	156	24	142	97.3%	0.87 [0.52, 1.47]
Kumaratne 2003	0	24	1	26	2.7%	0.36 [0.02, 8.43]
**Subtotal (95% CI)**		**180**		**168**	**100.0%**	**0.85 [0.51, 1.43]**
Total events	23		25			
Heterogeneity: Tau² = 0.00; Chi² = 0.30, df = 1 (P = 0.59); I² = 0%						
Test for overall effect: Z = 0.61 (P = 0.54)						
**4.1.5 moderate to severe**						
Ducbarme 1998	23	155	24	143	36.1%	0.88 [0.52, 1.49]
Iramain 2011	9	49	21	48	22.5%	0.42 [0.21, 0.82]
Peterson 1996	19	82	25	81	37.9%	0.75 [0.45, 1.25]
Watanasomsiri 2006	2	38	3	33	3.5%	0.58 [0.10, 3.26]
**Subtotal (95% CI)**		**324**		**305**	**100.0%**	**0.69 [0.50, 0.96]**
Total events	53		73			
Heterogeneity: Tau² = 0.00; Chi² = 3.10, df = 3 (P = 0.38); I² = 3%						
Test for overall effect: Z = 2.22 (P = 0.03)						
**4.1.6 unclear**						
Qureshi 1997	9	36	14	31	100.0%	0.55 [0.28, 1.10]
**Subtotal (95% CI)**		**36**		**31**	**100.0%**	**0.55 [0.28, 1.10]**
Total events	9		14			
Heterogeneity: Not applicable						
Test for overall effect: Z = 1.69 (P = 0.09)						

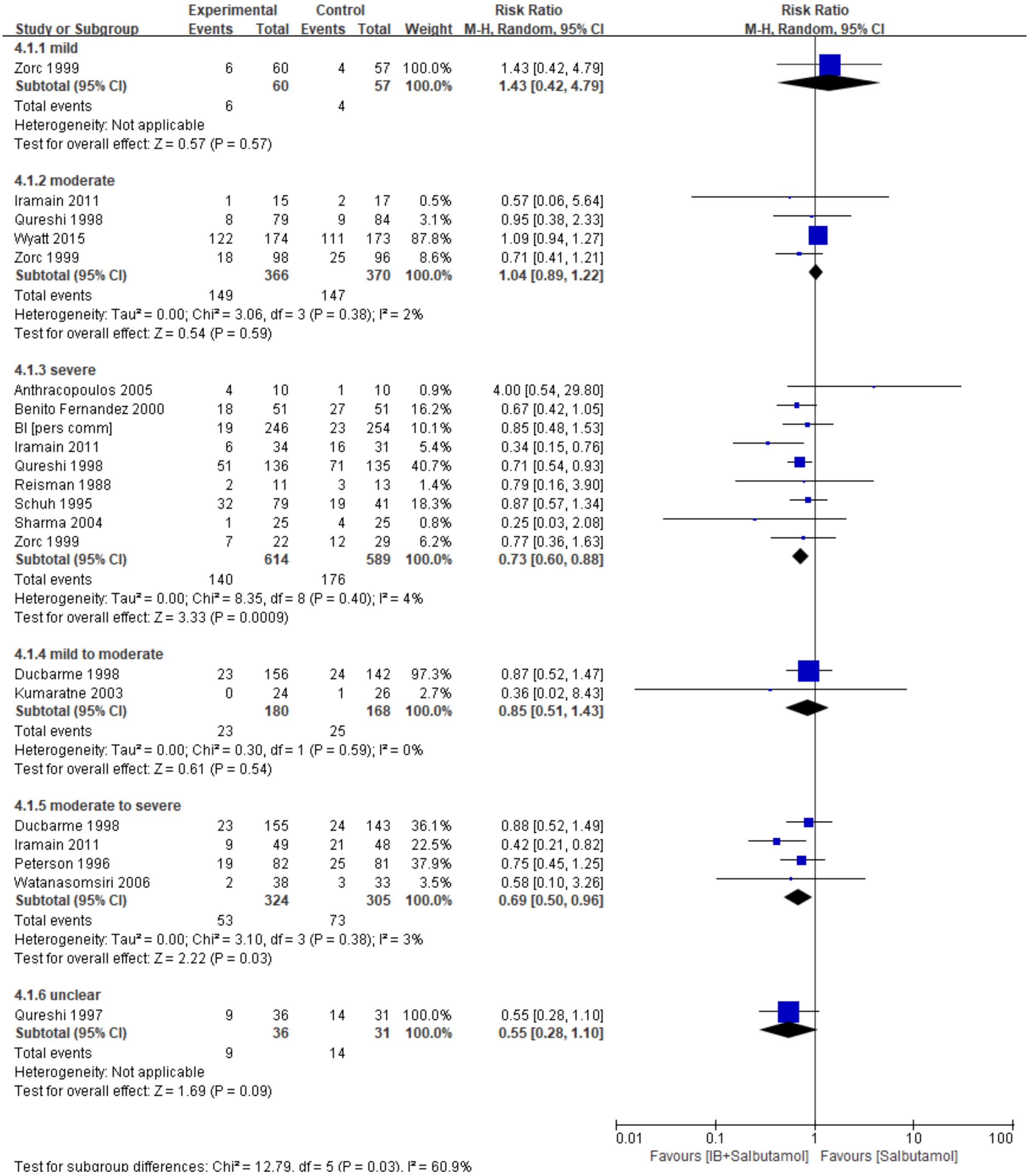

Test for subgroup differences: Chi² = 12.79, df = 5 (P = 0.03), I² = 60.9%

Subgroup of co-intervention

Study or Subgroup	Experimental Events	Experimental Total	Control Events	Control Total	Weight	Risk Ratio M-H, Random, 95% CI
**5.1.1 with steroid**						
Benito Fernandez 2000	18	51	27	51	13.7%	0.67 [0.42, 1.05]
Peterson 1996	19	82	25	81	11.8%	0.75 [0.45, 1.25]
Qureshi 1997	9	45	14	45	7.1%	0.64 [0.31, 1.33]
Qureshi 1998	59	215	80	219	21.3%	0.75 [0.57, 0.99]
Watanasomsiri 2006	2	38	3	33	1.5%	0.58 [0.10, 3.26]
Wyatt 2015	122	174	111	173	28.2%	1.09 [0.94, 1.27]
Zorc 1999	38	211	48	216	16.4%	0.81 [0.55, 1.19]
**Subtotal (95% CI)**		**816**		**818**	**100.0%**	**0.82 [0.66, 1.02]**
Total events	267		308			
Heterogeneity: Tau² = 0.04; Chi² = 12.88, df = 6 (P = 0.04); I² = 53%						
Test for overall effect: Z = 1.77 (P = 0.08)						
**5.1.2 without steroid**						
Schuh 1995	32	79	19	41	100.0%	0.87 [0.57, 1.34]
**Subtotal (95% CI)**		**79**		**41**	**100.0%**	**0.87 [0.57, 1.34]**
Total events	32		19			
Heterogeneity: Not applicable						
Test for overall effect: Z = 0.62 (P = 0.53)						
**5.1.3 mixed**						
Ducbarme 1998	23	155	24	143	100.0%	0.88 [0.52, 1.49]
**Subtotal (95% CI)**		**155**		**143**	**100.0%**	**0.88 [0.52, 1.49]**
Total events	23		24			
Heterogeneity: Not applicable						
Test for overall effect: Z = 0.46 (P = 0.65)						
**5.1.4 unclear**						
Anthracopoulos 2005	4	10	1	10	6.7%	4.00 [0.54, 29.80]
BI [pers comm]	19	246	23	254	39.7%	0.85 [0.48, 1.53]
Iramain 2011	9	49	21	48	34.5%	0.42 [0.21, 0.82]
Kumaratne 2003	0	24	1	26	2.9%	0.36 [0.02, 8.43]
Reisman 1988	2	11	3	13	10.0%	0.79 [0.16, 3.90]
Sharma 2004	1	25	4	25	6.1%	0.25 [0.03, 2.08]
**Subtotal (95% CI)**		**365**		**376**	**100.0%**	**0.67 [0.39, 1.15]**
Total events	35		53			
Heterogeneity: Tau² = 0.11; Chi² = 6.59, df = 5 (P = 0.25); I² = 24%						
Test for overall effect: Z = 1.46 (P = 0.14)						

Test for subgroup differences: Chi² = 0.73, df = 3 (P = 0.87), I² = 0%

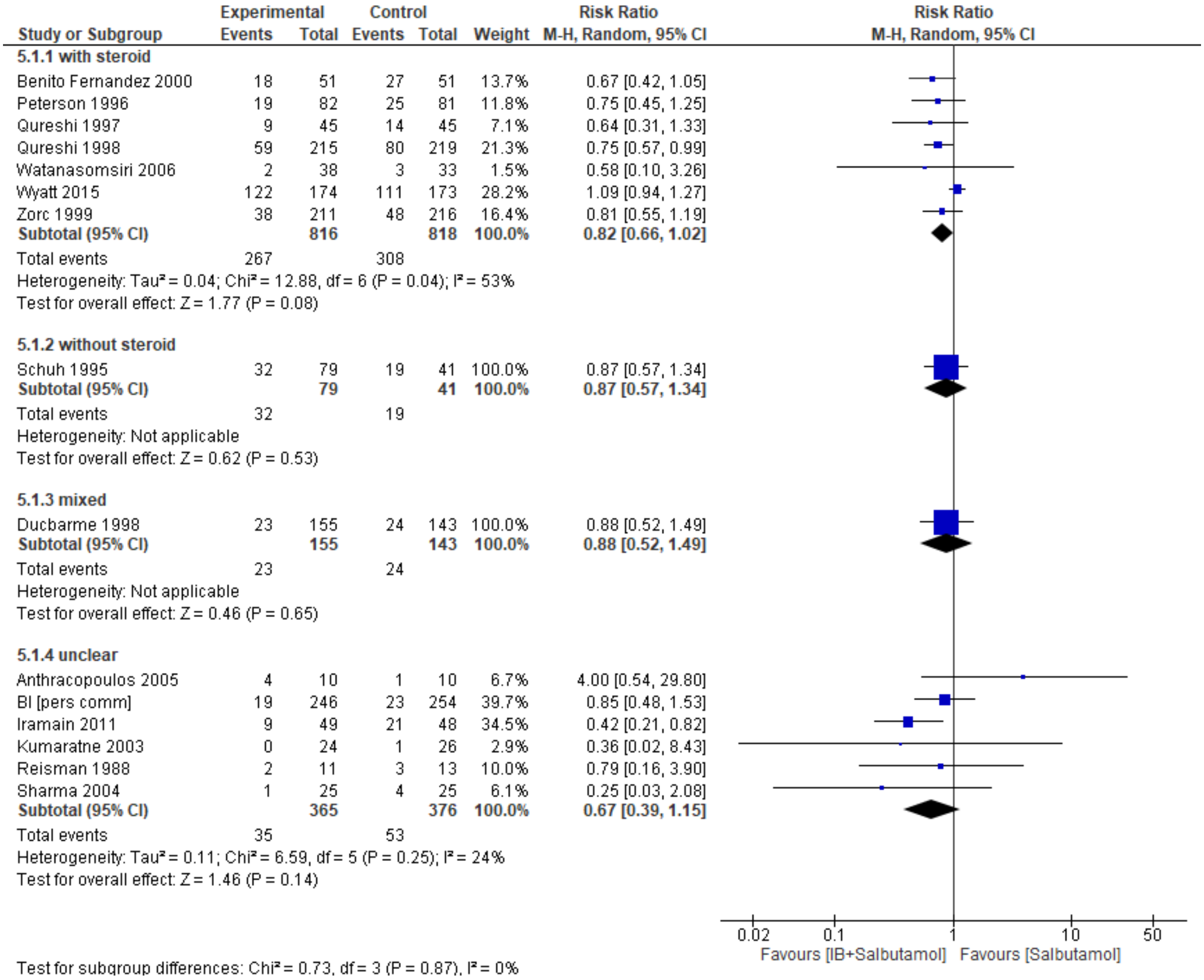

Supplement: S7 Appendix — (PDF) [file pone.0237620.s007.pdf]
