# Supplementary material for: Combination of ipratropium bromide and salbutamol in children and adolescents with asthma: A meta-analysis
Source: PLoS One. 2021 Feb 23;16(2):e0237620. doi: 10.1371/journal.pone.0237620 (PMC7901745; doi:10.1371/journal.pone.0237620)
Supplement: S8 Appendix — (PDF) [file pone.0237620.s008.pdf]

Appendix 8. Meta-analysis forest plots of primary outcome – any adverse event

Subgroup of age

Study or Subgroup	Experimental Events	Experimental Total	Control Events	Control Total	Weight	Risk Ratio M-H, Random, 95% CI
6 yrs old and over						
Yi 2015	2	42	0	42	100.0%	5.00 [0.25, 101.11]
**Subtotal (95% CI)**		**42**		**42**	**100.0%**	**5.00 [0.25, 101.11]**
Total events	2		0			
Heterogeneity: Not applicable						
Test for overall effect: Z = 1.05 (P = 0.29)						
mixed						
Qureshi 1997	32	45	35	45	36.4%	0.91 [0.72, 1.17]
Wyatt 2015	23	174	8	173	31.1%	2.86 [1.31, 6.21]
Yin 2018	3	38	4	38	22.4%	0.75 [0.18, 3.13]
Zhang 2012	5	50	0	50	10.1%	11.00 [0.62, 193.80]
**Subtotal (95% CI)**		**307**		**306**	**100.0%**	**1.60 [0.55, 4.68]**
Total events	63		47			
Heterogeneity: Tau² = 0.80; Chi² = 15.71, df = 3 (P = 0.001); I² = 81%						
Test for overall effect: Z = 0.86 (P = 0.39)						

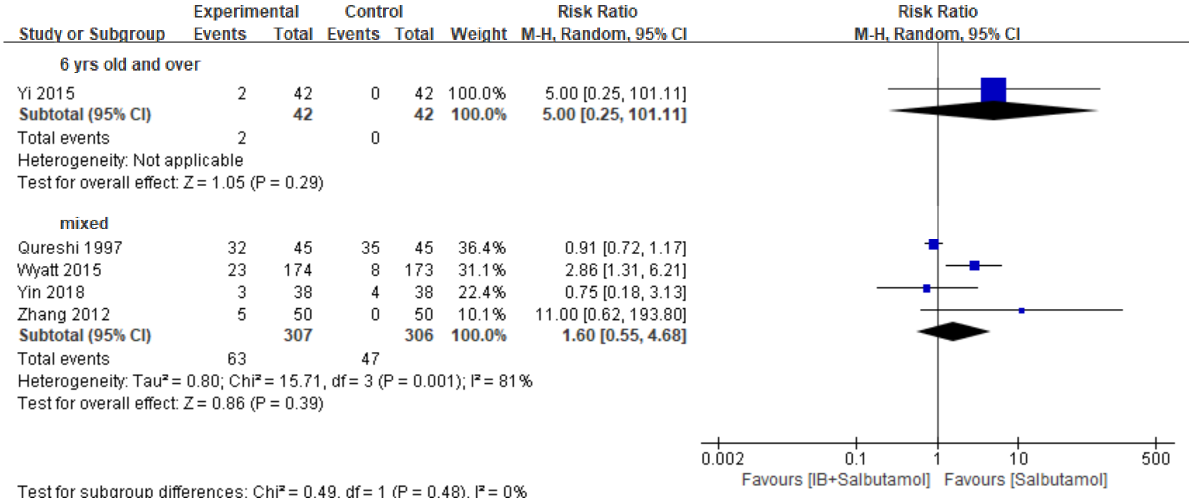

Test for subgroup differences: Chi² = 0.49, df = 1 (P = 0.48), I² = 0%

Subgroup of severity

Study or Subgroup	Experimental Events	Experimental Total	Control Events	Control Total	Weight	Risk Ratio M-H, Random, 95% CI
**moderate**						
Wyatt 2015	23	174	8	173	100.0%	2.86 [1.31, 6.21]
**Subtotal (95% CI)**		**174**		**173**	**100.0%**	**2.86 [1.31, 6.21]**
Total events	23		8			
Heterogeneity: Not applicable						
Test for overall effect: Z = 2.65 (P = 0.008)						
**unclear**						
Qureshi 1997	32	45	35	45	54.6%	0.91 [0.72, 1.17]
Yi 2015	2	42	0	42	9.2%	5.00 [0.25, 101.11]
Yin 2018	3	38	4	38	26.2%	0.75 [0.18, 3.13]
Zhang 2012	5	50	0	50	10.0%	11.00 [0.62, 193.80]
**Subtotal (95% CI)**		**175**		**175**	**100.0%**	**1.30 [0.48, 3.53]**
Total events	42		39			
Heterogeneity: Tau² = 0.46; Chi² = 5.48, df = 3 (P = 0.14); I² = 45%						
Test for overall effect: Z = 0.52 (P = 0.61)						

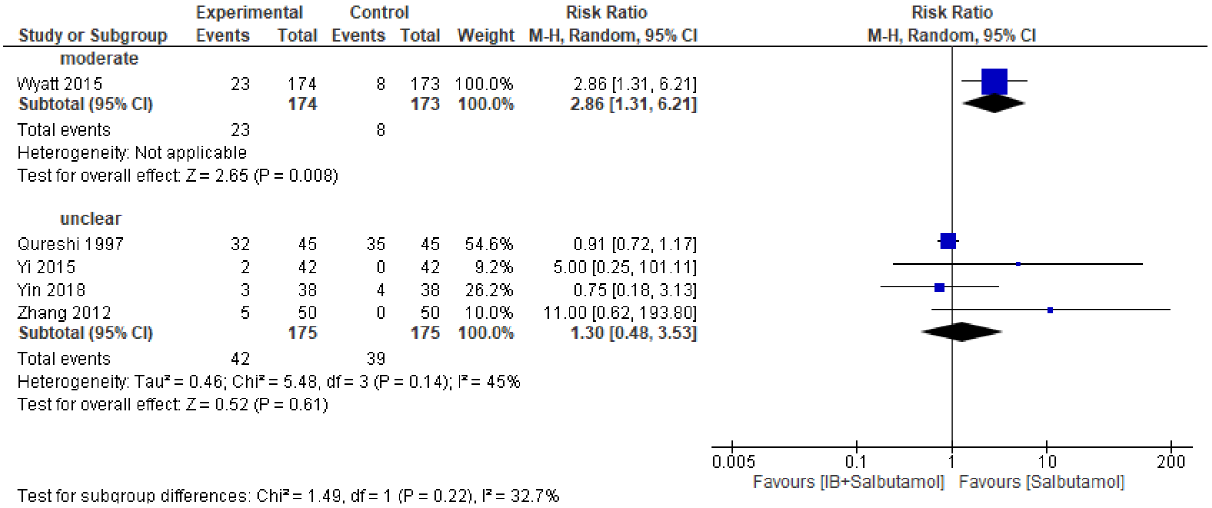

Test for subgroup differences: Chi² = 1.49, df = 1 (P = 0.22), I² = 32.7%

Subgroup of co-intervention

Study or Subgroup	Experimental Events	Experimental Total	Control Events	Control Total	Weight	Risk Ratio M-H, Random, 95% CI
**with steroid**						
Qureshi 1997	32	45	35	45	53.6%	0.91 [0.72, 1.17]
Wyatt 2015	23	174	8	173	46.4%	2.86 [1.31, 6.21]
**Subtotal (95% CI)**		**219**		**218**	**100.0%**	**1.55 [0.39, 6.10]**
Total events	55		43			
Heterogeneity: Tau² = 0.90; Chi² = 11.39, df = 1 (P = 0.0007); I² = 91%						
Test for overall effect: Z = 0.63 (P = 0.53)						
**with standard care**						
Yi 2015	2	42	0	42	47.7%	5.00 [0.25, 101.11]
Zhang 2012	5	50	0	50	52.3%	11.00 [0.62, 193.80]
**Subtotal (95% CI)**		**92**		**92**	**100.0%**	**7.55 [0.95, 60.21]**
Total events	7		0			
Heterogeneity: Tau² = 0.00; Chi² = 0.14, df = 1 (P = 0.71); I² = 0%						
Test for overall effect: Z = 1.91 (P = 0.06)						
**unclear**						
Yin 2018	3	38	4	38	100.0%	0.75 [0.18, 3.13]
**Subtotal (95% CI)**		**38**		**38**	**100.0%**	**0.75 [0.18, 3.13]**
Total events	3		4			
Heterogeneity: Not applicable						
Test for overall effect: Z = 0.39 (P = 0.69)						

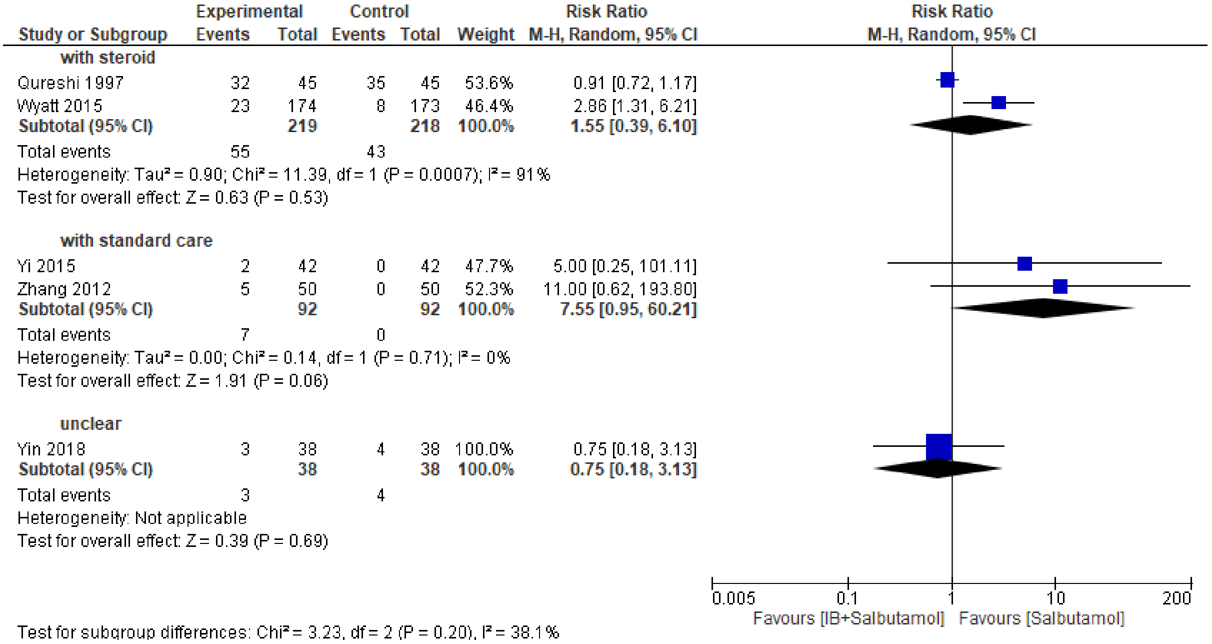

Test for subgroup differences: Chi² = 3.23, df = 2 (P = 0.20), I² = 38.1%
