# Supplementary material for: Combination of ipratropium bromide and salbutamol in children and adolescents with asthma: A meta-analysis
Source: PLoS One. 2021 Feb 23;16(2):e0237620. doi: 10.1371/journal.pone.0237620 (PMC7901745; doi:10.1371/journal.pone.0237620)
Supplement: S9 Appendix — (PDF) [file pone.0237620.s009.pdf]

Appendix 9. Meta-analysis forest plots of secondary outcome.

Oxygen saturation (%)

Study or Subgroup	IB and Salbutamol Mean	SD	Total	Salbutamol Mean	SD	Total	Weight	Mean Difference IV, Random, 95% CI
Chakraborti 2006	96.97	2.08	30	96.4	1.69	30	26.3%	0.57 [-0.39, 1.53]
Coskun 2001	97.8	2.9	23	97.6	3.9	20	18.2%	0.20 [-1.88, 2.28]
Iramain 2011	95	2.4	49	92.2	1.9	48	26.9%	2.80 [1.94, 3.66]
Qureshi 1998	97.4	1.8	79	97.3	1.8	84	28.6%	0.10 [-0.45, 0.65]
**Total (95% CI)**			**181**			**182**	**100.0%**	**0.97 [-0.45, 2.39]**

Heterogeneity: Tau² = 1.76; Chi² = 27.45, df = 3 (P < 0.00001); I² = 89%
Test for overall effect: Z = 1.34 (P = 0.18)

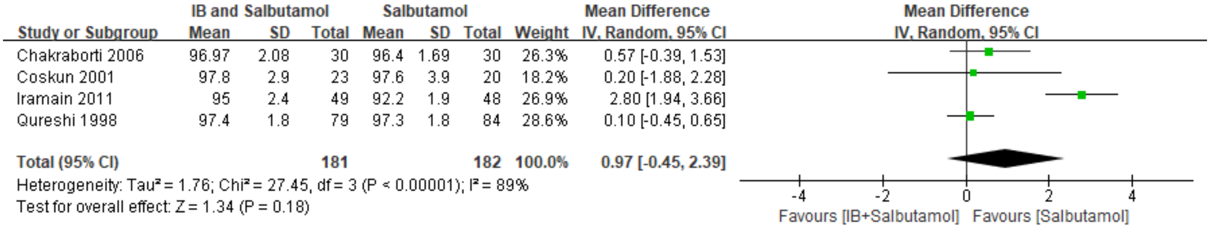

Need for repeated bronchodilator treatments

Study or Subgroup	IB and Salbutamol Events	Total	Salbutamol Events	Total	Weight	Risk Ratio M-H, Random, 95% CI
Benito Fernandez 2000	6	51	9	51	1.3%	0.67 [0.26, 1.74]
Peterson 1996	68	82	73	81	79.1%	0.92 [0.81, 1.04]
Schuh 1995	31	39	30	41	19.6%	1.09 [0.85, 1.39]
**Total (95% CI)**		**172**		**173**	**100.0%**	**0.95 [0.85, 1.06]**
Total events	105		112			

Heterogeneity: Tau² = 0.00; Chi² = 1.95, df = 2 (P = 0.38); I² = 0%
Test for overall effect: Z = 0.99 (P = 0.32)

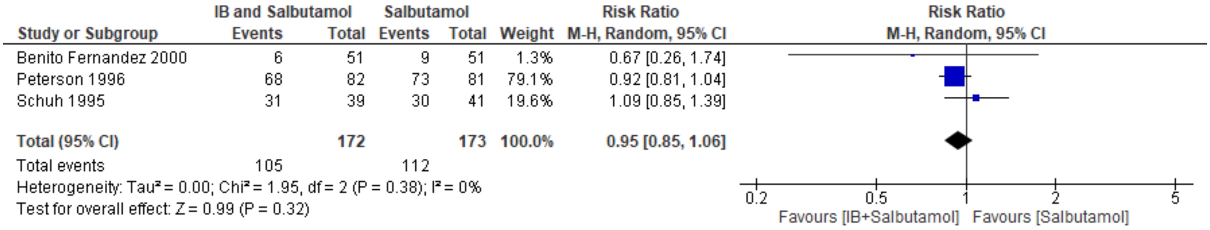

Need for systemic corticosteroids

Study or Subgroup	IB and Salbutamol Events	Total	Salbutamol Events	Total	Weight	Risk Ratio M-H, Random, 95% CI
Ducbarme 1998	85	155	86	143	70.8%	0.91 [0.75, 1.11]
Rayner 1987	14	19	14	18	20.3%	0.95 [0.66, 1.36]
Storr 1986	19	68	18	70	8.9%	1.09 [0.63, 1.89]
**Total (95% CI)**		**242**		**231**	**100.0%**	**0.93 [0.79, 1.10]**
Total events	118		118			

Heterogeneity: Tau² = 0.00; Chi² = 0.36, df = 2 (P = 0.83); I² = 0%
Test for overall effect: Z = 0.82 (P = 0.41)

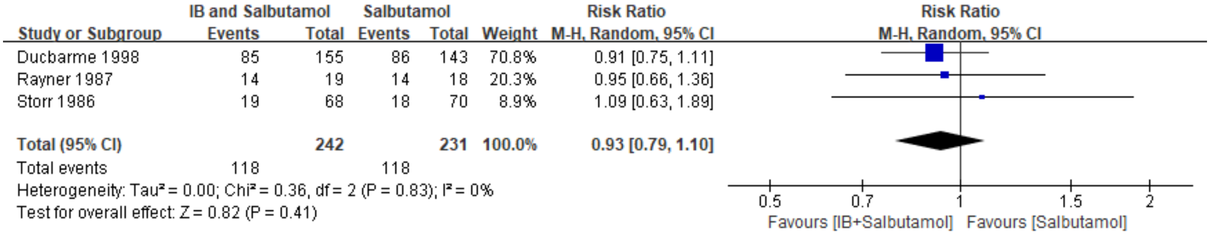

Relapse rate

Study or Subgroup	Experimental Events	Total	Control Events	Total	Weight	Risk Ratio M-H, Random, 95% CI
BI [pers comm]	2	246	1	254	2.5%	2.07 [0.19, 22.63]
Ducbarme 1998	20	155	13	143	32.3%	1.42 [0.73, 2.75]
Kumaratne 2003	0	24	1	26	1.4%	0.36 [0.02, 8.43]
Peterson 1996	7	63	9	57	16.6%	0.70 [0.28, 1.77]
Qureshi 1997	1	45	0	45	1.4%	3.00 [0.13, 71.74]
Qureshi 1998	8	215	6	219	13.0%	1.36 [0.48, 3.85]
Reisman 1988	2	11	3	13	5.5%	0.79 [0.16, 3.90]
Schuh 1995	1	40	3	41	2.9%	0.34 [0.04, 3.15]
Sharma 2004	1	25	4	25	3.1%	0.25 [0.03, 2.08]
Watanasomsiri 2006	4	38	7	33	10.9%	0.50 [0.16, 1.55]
Zorc 1999	9	211	4	216	10.4%	2.30 [0.72, 7.37]
**Total (95% CI)**		**1073**		**1072**	**100.0%**	**1.04 [0.71, 1.51]**
Total events	55		51			

Heterogeneity: Tau² = 0.00; Chi² = 9.22, df = 10 (P = 0.51); I² = 0%
Test for overall effect: Z = 0.19 (P = 0.85)

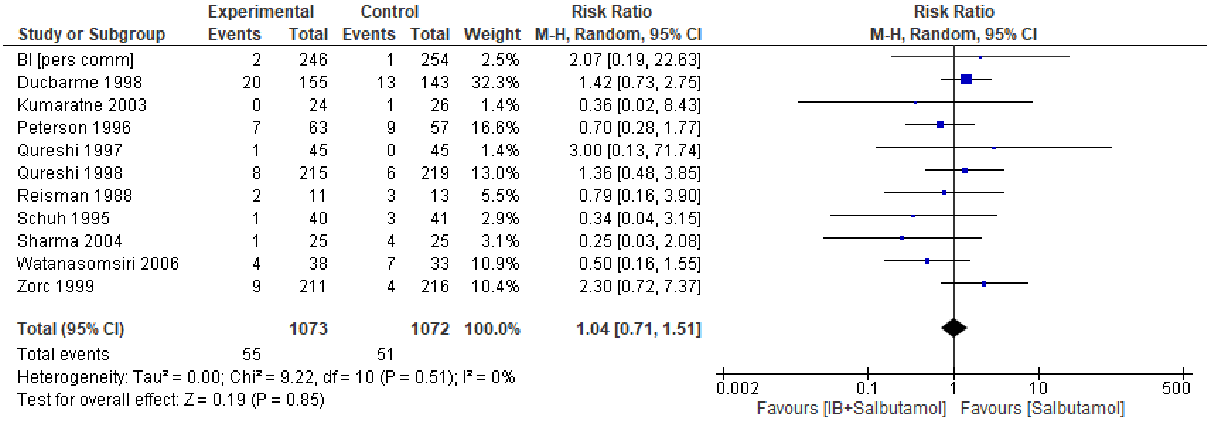

Specific adverse events

Specific adverse events	N of Studies	N of participants	RR [95% CI]	P value
Abdominal pain	1	347	2.98 [0.12, 72.72]	0.50
Back or chest pain	1	90	1.50 [0.26, 8.55]	0.65
Bad taste	2	64	1.60 [0.51, 5.05]	0.43
Blurred vision	1	40	4.00 [0.49, 32.72]	0.20
Dizziness	1	90	0.40 [0.08, 1.96]	0.26
Dry mouth	6	380	1.01 [0.75, 1.36]	0.94
Flushed face	1	20	4.17 [0.23, 77.11]	0.34
Headache	2	161	1.26 [0.48, 3.29]	0.64
Hoarseness	1	114	1.93 [0.62, 6.05]	0.26
Hyperactivity	1	347	2.98 [0.31, 28.40]	0.34
Increased heart rate	1	114	0.80 [0.38, 1.71]	0.57
Nausea	6	993	0.60 [0.39, 0.93]	0.02*
Palpitations	2	130	1.42 [0.70, 2.90]	0.33
Presyncope	1	347	2.98 [0.12, 72.72]	0.50
Restless	1	76	0.20 [0.01, 4.03]	0.29
Transient tachycardia	1	20	0.17 [0.01, 3.08]	0.23
Transient eye irritation	1	50	0.20 [0.01, 3.97]	0.29
Tremor	7	763	1.09 [0.72, 1.64]	0.68
Vomit	8	1491	1.10 [0.56, 2.13]	0.79

**significant result with p<0.05.*

Specific adverse events (dry mouth, nausea, tremor, and vomit)

	IB and Salbutamol		Salbutamol			Risk Ratio
**Study or Subgroup**	**Events**	**Total**	**Events**	**Total**	**Weight**	**M-H, Random, 95% CI**
**Dry mouth**						
Dai 2000	2	11	0	9	1.0%	4.17 [0.23, 77.11]
Li 2000	2	20	3	20	3.1%	0.67 [0.12, 3.57]
Luo 2004	7	58	2	56	3.7%	3.38 [0.73, 15.57]
Qureshi 1997	27	45	28	45	80.4%	0.96 [0.69, 1.34]
Watson 1994	6	20	7	20	10.9%	0.86 [0.35, 2.10]
Yin 2018	1	38	0	38	0.9%	3.00 [0.13, 71.40]
**Subtotal (95% CI)**		**192**		**188**	**100.0%**	**1.01 [0.75, 1.36]**
Total events	45		40			
Heterogeneity: Tau² = 0.00; Chi² = 4.59, df = 5 (P = 0.47); I² = 0%						
Test for overall effect: Z = 0.07 (P = 0.94)						
**Nausea**						
Ducbarme 1998	12	122	21	119	44.4%	0.56 [0.29, 1.08]
Peterson 1996	1	82	1	81	2.6%	0.99 [0.06, 15.53]
Qureshi 1997	2	45	3	45	6.4%	0.67 [0.12, 3.80]
Schuh 1995	8	40	15	41	35.7%	0.55 [0.26, 1.14]
Watanasomsiri 2006	1	38	0	33	1.9%	2.62 [0.11, 62.10]
Wyatt 2015	3	174	4	173	8.9%	0.75 [0.17, 3.28]
**Subtotal (95% CI)**		**501**		**492**	**100.0%**	**0.60 [0.39, 0.93]**
Total events	27		44			
Heterogeneity: Tau² = 0.00; Chi² = 1.17, df = 5 (P = 0.95); I² = 0%						
Test for overall effect: Z = 2.26 (P = 0.02)						
**Tremor**						
Beck 1985	7	13	5	12	19.8%	1.29 [0.56, 2.99]
Luo 2004	4	58	3	56	7.4%	1.29 [0.30, 5.49]
Peterson 1996	4	82	3	81	7.3%	1.32 [0.30, 5.70]
Reisman 1988	6	11	7	13	24.4%	1.01 [0.48, 2.12]
Sharma 2004	4	25	8	25	13.1%	0.50 [0.17, 1.45]
Watson 1994	7	20	8	20	21.2%	0.88 [0.39, 1.95]
Wyatt 2015	10	174	2	173	6.9%	4.97 [1.11, 22.36]
**Subtotal (95% CI)**		**383**		**380**	**100.0%**	**1.09 [0.72, 1.64]**
Total events	42		36			
Heterogeneity: Tau² = 0.04; Chi² = 6.79, df = 6 (P = 0.34); I² = 12%						
Test for overall effect: Z = 0.41 (P = 0.68)						
**Vomit**						
Beck 1985	1	13	1	12	5.8%	0.92 [0.06, 13.18]
BI [pers comm]	3	246	0	254	4.7%	7.23 [0.38, 139.18]
Ducbarme 1998	9	154	12	138	33.4%	0.67 [0.29, 1.55]
Peterson 1996	4	82	2	81	13.0%	1.98 [0.37, 10.49]
Qureshi 1997	2	45	3	45	12.1%	0.67 [0.12, 3.80]
Reisman 1988	1	11	2	13	7.7%	0.59 [0.06, 5.68]
Sharma 2004	1	25	3	25	8.2%	0.33 [0.04, 2.99]
Wyatt 2015	9	174	2	173	15.1%	4.47 [0.98, 20.41]
**Subtotal (95% CI)**		**750**		**741**	**100.0%**	**1.10 [0.56, 2.13]**
Total events	30		25			
Heterogeneity: Tau² = 0.17; Chi² = 8.53, df = 7 (P = 0.29); I² = 18%						
Test for overall effect: Z = 0.27 (P = 0.79)						

Test for subgroup differences: Chi² = 4.87, df = 3 (P = 0.18), I² = 38.4%

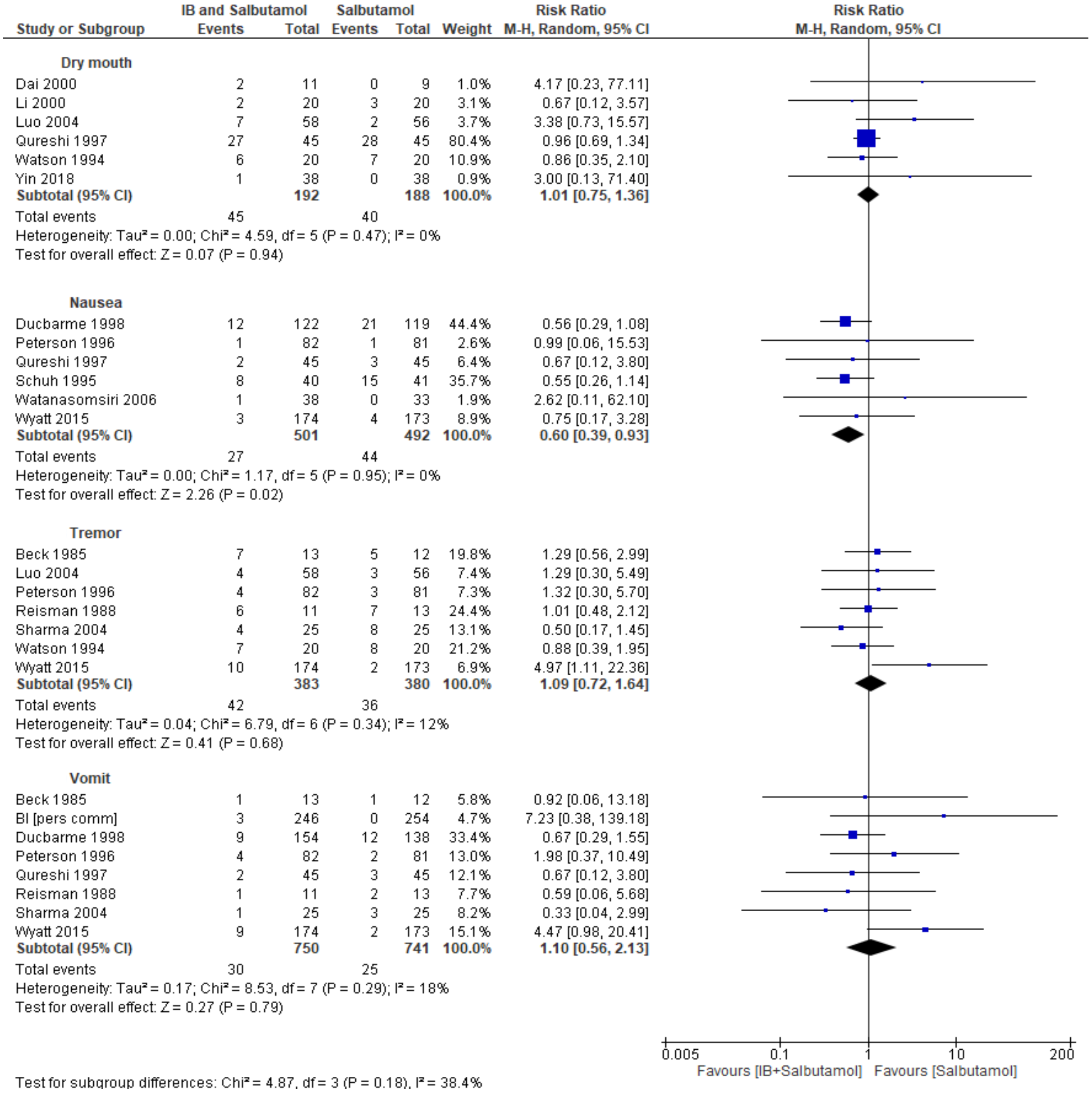

Summary of secondary outcome – pulmonary function at 30 min, 240 min, 5 days and 7 days after treatment

Study	Mean age, year (range)	Diagnosis	Patients	Intervention & Comparison	Significant key results
Iramain 2011 Spain	9.1 (2-18)	asthma	106	IB: 250 μg (< 20 kg); 500 μg (≥20 kg)+salbutamol: 2.5 mg (< 20 kg); 5 mg (≥20 kg) vs. Salbutamol: 2.5 mg (< 20 kg); 5 mg (≥20 kg), every 20 minutes for 120 minutes	IB+ salbutamol improved predict FEV1 % significantly at 240 mins (97 vs. 69) after treatment.
Liao 2019 China	8.9 (6-11)	asthma	132	IB: 62.5 μg+ Salbutamol: 2.5 mg vs. Salbutamol: 2.5 mg; 4 times/day for 7 days	IB+ salbutamol improved predict FEV1 % (98.49 vs. 91.65) significantly after 7 days of treatment.
Anthracopoulos 2005 Greece	9.7 (7-13)	asthma	20	IB: 5 μg/kg/dose (max: 250μg)+Salbutamol: 0.1mg/kg/dose (max: 5mg) vs. Salbutamol: 0.1mg/kg/dose (max: 5mg); 3 doses within 1 hour	No significant differences in predict FEV1 % at 15 minutes after each nebulization treatment.
Chakraborti 2006 India	9.3 (NR)	asthma	60	IB: 80 μg + Salbutamol: 400 μg vs. Salbutamol: 400 μg+Placebo: 80 μg	No significant differences in FEV1 % at 30 mins after the treatment.
Liu 2012 China	7.4 (5-12)	acute asthma	62	IB: 125 mg (<6 yrs); 250 mg (≥6 yrs) +Salbutamol: 2.5 mg (4-8 yrs); 3.75 mg(8-12 yrs); 5 mg (>12 yrs) vs. Salbutamol: 2.5 mg (4-8 yrs); 3.75 mg(8-12 yrs); 5 mg (>12 yrs). Both with Budesonide: 0.5 mg. Twice/day for 5 days	IB+ salbutamol improved FEV1 % (96.29 vs. 90.03) significantly after 5 days of treatment.

Summary of secondary outcome – clinical scores

Study	Mean age, year (range)	Diagnosis	Patients	Intervention & Comparison	Significant key results
Iramain 2011 Spain	9.1 (2-18)	Asthma	106	IB: 250 µg (< 20 kg); 500 µg (≥20 kg)+salbutamol: 2.5 mg (< 20 kg); 5 mg (≥20 kg) vs. Salbutamol: 2.5 mg (< 20 kg); 5 mg (≥20 kg), every 20 minutes for 120 minutes	Improved clinical score (asthma score) at 240 mins after treatment.
Kumaratne 2003 California	1.95 (4 months-6)	Acute asthma	50	IB: 250 µg (< 15kg); 500 µg (≥15kg)+Salbutamol: 0.15 mg/kg vs. Salbutamol: 0.15 mg/kg, the solutions were diluted to 4 mL with normal saline, 20 min/time	No significant differences in clinical score (asthma score) at 30 minutes after the single treatment.
Li 2000 China	9.3 (NR)	Acute asthma	40	Salbutamol (0.5%): 2.5-5 mg (according to patients' age) with IB (0.025%): 0.25-0.5 mg (according to patients' age) vs. Salbutamol (0.5%): 2.5-5 mg (according to patients' age); 5-10 minutes/time	No significant differences in clinical score (wheeze score/wheezing sound score) at 10mins, 60 mins and 120 mins after the treatment.
Liao 2019 China	8.9 (6-11)	Asthma	132	IB: 62.5 µg+ Salbutamol: 2.5 mg vs. Salbutamol: 2.5 mg; 4 times/day for 7 days	Improved clinical score (cough score/wheezing sound score) after 7 days of treatment.<br>No significant differences in clinical score (wheeze score) after 7 days of treatment.
Ji 2003 China	NR (8 months-9)	Acute asthma	70	IB: 0.5 mL (<4 yrs); 1.0 mL (4-8 yrs); 1.5 mL (8-12 yrs)+Salbutamol: 0.25 mL (<4 yrs); 0.5 mL (4-8 yrs); 0.75 mL (8-12 yrs)+Budesonide: 1mg vs. Salbutamol: 0.25 (<4 yrs); 0.5 mL (4-8 yrs); 0.75 mL (8-12 yrs)+Budesonide: 1mg, 2 times/day	Improved clinical score (cough score/dysponea score/wheeze score/ wheezing sound score: P < 0.05) at 30 mins after the treatment.
Chakraborti 2006 India	9.3 (NR)	Asthma	60	IB: 80 µg + Salbutamol: 400 µg vs. Salbutamol: 400 µg+Placebo: 80 µg	No significant differences in clinical score (wheeze score) at 30 mins after the treatment.
Luo 2004 China	7.5 (NR)	Asthma	114	IB: 0.5mL (<4 yrs); 1.0 mL (4-8 yrs); 1.5 mL (8-12 yrs); 2.0mL (> 12 yrs)+Salbutamol: 0.25 mL (<4 yrs); 0.5 mL (4-8 yrs); 0.75 mL (8-12 yrs); 1.0 ml (> 12 yrs)+Budesonide: 1mg vs. Salbutamol: 0.25 mL (<4 yrs); 0.5 mL (4-8 yrs); 0.75 mL (8-12 yrs); 1.0 ml (> 12 yrs)+Budesonide: 1mg, 2 times/day	Improved clinical score (decreased asthma score: 3.3 vs. 2.1, MD 1.2, P < 0.05) after treatment.
Luo 2014 China	7.4 (5-14)	Asthma	156	IB (0.025%): 0.5 ml+Salbutamol (0.5%): 0.02 ml/kg vs. Salbutamol (0.5%): 0.02 ml/kg 0.9 %, Nacl was added to each treatment dose to achieve a total volume of 2 mL, 5 ml/time, 2 times/day for one month	Improved clinical score (daytime symptom scores; nighttime symptom scores) after one month of treatment.
Menon 2016 Pakistan	9.2 (2-14)	Asthma	200	IB: 250 µg/kg/dose+Salbutamol: 0.03 ml/kg/dose vs. Salbutamol: 0.03 ml/kg/dose, every 15 minutes for 45 minutes	No significant differences in clinical score (wheeze score) after 15 minutes of the last dose (about 45 mins of treatment).
Rayner 1987 United Kingdom	6.5 (2-15)	Asthma	40	IB: 250 µg+Salbutamol: 2.5 mg, or 5 mg (over 6 years) (Salbutamol was used on admission and four hourly thereafter; IB was used at 30 minutes after first dose of salbutamol and eight hourly thereafter.) vs. Salbutamol: 2.5 mg, or 5 mg (over 6 years)+Saline: 3 ml (Salbutamol was used on admission and four hourly thereafter; saline was used at 30 minutes after first dose of salbutamol and eight hourly thereafter)	No significant differences in clinical score (based on clinical examination, activity, and speech) at 45 minutes after the first administration of the trial drug and the next morning (12-24 hours later).
Coskun 2001 Turkey	7 (4-15)	Asthma	43	IB: 250 µg+Albuterol: 0.075 mg/kg, maximum 2.5 mg vs. Albuterol: 0.075 mg/kg, maximum 2.5 mg, every 20 minutes for 40 minutes	No significant differences in clinical scores (respiratory distress scores) after treatment (about 60 mins of treatment).
