# Supplementary material for: Combination of ipratropium bromide and salbutamol in children and adolescents with asthma: A meta-analysis
Source: PLoS One. 2021 Feb 23;16(2):e0237620. doi: 10.1371/journal.pone.0237620 (PMC7901745; doi:10.1371/journal.pone.0237620)
Supplement: S10 Appendix — (PDF) [file pone.0237620.s010.pdf]

Summary of findings:

Ipratropium bromide and Salbutamol compared to Salbutamol for Treatment of Asthma in Children

Patient or population: Treatment of Asthma in Children

Setting:

Intervention: Ipratropium bromide and Salbutamol

Comparison: Salbutamol

Outcomes	Anticipated absolute effects* (95% CI)		Relative effect (95% CI)	№ of participants (studies)	Certainty of the evidence (GRADE)	Comments
	Risk with Salbutamol	Risk with Ipratropium bromide and Salbutamol				
Hospital Admission	294 per 1,000	**232 per 1,000** (194 to 279)	**RR 0.79** (0.66 to 0.95)	2754 (15 RCTs)	⨁⨁◯◯ LOW [a,b]	
Specific adverse events - Dry mouth	213 per 1,000	**215 per 1,000** (160 to 289)	**RR 1.01** (0.75 to 1.36)	380 (6 RCTs)	⨁⨁◯◯ LOW [a,d]	
Specific adverse events - Nausea	89 per 1,000	**54 per 1,000** (35 to 83)	**RR 0.60** (0.39 to 0.93)	993 (6 RCTs)	⨁⨁⨁⨁ HIGH	
Specific adverse events - Tremor	95 per 1,000	**103 per 1,000** (68 to 155)	**RR 1.09** (0.72 to 1.64)	763 (7 RCTs)	⨁⨁⨁◯ MODERATE [a]	
Specific adverse events - Vomit	34 per 1,000	**37 per 1,000** (19 to 72)	**RR 1.10** (0.56 to 2.13)	1491 (8 RCTs)	⨁⨁⨁◯ MODERATE [c]	
Predicted forced expiratory volume in one second (predicted FEV1 in %, endpoint data) - 60 mins after the combined ipratropium bromide and salbutamol assessed with: percentage	One study showed the predict FEV1 significantly improved 11.61 % at 60 mins after the combined IB and salbutamol treatment. The other two studies showed no significant difference at 60 mins after the combined IB and salbutamol treatment.			351 (3 RCTs)	⨁⨁⨁⨁ HIGH	

Summary of findings:

Ipratropium bromide and Salbutamol compared to Salbutamol for Treatment of Asthma in Children

Patient or population: Treatment of Asthma in Children

Setting:

Intervention: Ipratropium bromide and Salbutamol

Comparison: Salbutamol

Outcomes	Anticipated absolute effects* (95% CI)		Relative effect (95% CI)	№ of participants (studies)	Certainty of the evidence (GRADE)	Comments
	Risk with Salbutamol	Risk with Ipratropium bromide and Salbutamol				
Predicted forced expiratory volume in one second (predicted FEV1 in %, endpoint data) - 120 mins after the combined ipratropium bromide and salbutamol assessed with: percentage	This study showed the predict FEV1 significantly improved 7.781% at 120 mins after the treatment of combined IB and salbutamol.			98 (1 RCT)	⨁⨁⨁◯ MODERATE [d]	

***The risk in the intervention group** (and its 95% confidence interval) is based on the assumed risk in the comparison group and the **relative effect** of the intervention (and its 95% CI).

CI: Confidence interval; **RR:** Risk ratio; **MD:** Mean difference

GRADE Working Group grades of evidence
High certainty: We are very confident that the true effect lies close to that of the estimate of the effect
Moderate certainty: We are moderately confident in the effect estimate: The true effect is likely to be close to the estimate of the effect, but there is a possibility that it is substantially different
Low certainty: Our confidence in the effect estimate is limited: The true effect may be substantially different from the estimate of the effect
Very low certainty: We have very little confidence in the effect estimate: The true effect is likely to be substantially different from the estimate of effect

Explanations

a. Downgrade once for risk of bias because the lack of information on blinding of participants and outcomes.

b. Downgrade once for publication bias because the Egger's test indicated the presence of funnel plot asymmetry.

c. Downgrade once for imprecision because the confidence interval crosses threshold.

d. Downgrade once for imprecision because of less than 200 participants in each treatment arm.
