# Supplementary material for: Combination of ipratropium bromide and salbutamol in children and adolescents with asthma: A meta-analysis
Source: PLoS One. 2021 Feb 23;16(2):e0237620. doi: 10.1371/journal.pone.0237620 (PMC7901745; doi:10.1371/journal.pone.0237620)

Appendix 11. Sensitivity analysis for primary outcomes

Sensitivity analysis with the assumption of lost binary data

Hospital admittion – with ITT dataset

Study or Subgroup	IB+Salbutamol Events	IB+Salbutamol Total	Salbutamol Events	Salbutamol Total	Weight	Risk Ratio M-H, Random, 95% CI
Anthracopoulos 2005	4	10	1	10	0.7%	4.00 [0.54, 29.80]
Benito Fernandez 2000	18	51	27	51	8.7%	0.67 [0.42, 1.05]
BI [pers comm]	19	246	23	254	6.1%	0.85 [0.48, 1.53]
Ducbarme 1998	23	155	24	143	7.1%	0.88 [0.52, 1.49]
Iramain 2011	13	53	27	53	6.8%	0.48 [0.28, 0.83]
Kumaratne 2003	0	24	1	26	0.3%	0.36 [0.02, 8.43]
Peterson 1996	19	82	25	81	7.4%	0.75 [0.45, 1.25]
Qureshi 1997	9	45	14	45	4.3%	0.64 [0.31, 1.33]
Qureshi 1998	87	243	98	237	16.7%	0.87 [0.69, 1.09]
Reisman 1988	2	11	3	13	1.0%	0.79 [0.16, 3.90]
Schuh 1995	15	40	19	41	7.2%	0.81 [0.48, 1.36]
Sharma 2004	1	25	4	25	0.6%	0.25 [0.03, 2.08]
Watanasomsiri 2006	2	38	3	33	0.9%	0.58 [0.10, 3.26]
Wyatt 2015	157	209	145	207	21.6%	1.07 [0.95, 1.21]
Zorc 1999	38	211	48	216	10.7%	0.81 [0.55, 1.19]
**Total (95% CI)**		**1443**		**1435**	**100.0%**	**0.82 [0.69, 0.97]**
Total events	407		462			

Heterogeneity: $Tau^2 = 0.03$; $Chi^2 = 22.89$, df = 14 (P = 0.06); $I^2 = 39\%$
Test for overall effect: Z = 2.35 (P = 0.02)

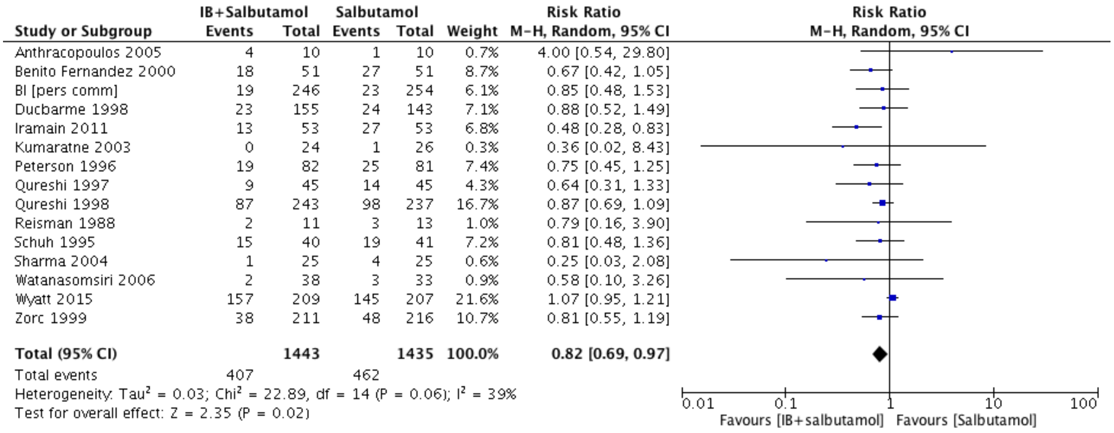

Hospital admission – without ITT dataset

Study or Subgroup	IB+salbutamol Events	IB+salbutamol Total	Salbutamol Events	Salbutamol Total	Weight	Risk Ratio M-H, Random, 95% CI
Anthracopoulos 2005	4	10	1	10	0.8%	4.00 [0.54, 29.80]
Benito Fernandez 2000	18	51	27	51	9.4%	0.67 [0.42, 1.05]
BI [pers comm]	19	246	23	254	6.8%	0.85 [0.48, 1.53]
Ducbarme 1998	23	155	24	143	7.8%	0.88 [0.52, 1.49]
Iramain 2011	9	49	22	48	5.6%	0.40 [0.21, 0.78]
Kumaratne 2003	0	24	1	26	0.3%	0.36 [0.02, 8.43]
Peterson 1996	19	82	25	81	8.1%	0.75 [0.45, 1.25]
Qureshi 1997	9	45	14	45	4.9%	0.64 [0.31, 1.33]
Qureshi 1998	59	215	80	219	14.7%	0.75 [0.57, 0.99]
Reisman 1988	2	11	3	13	1.2%	0.79 [0.16, 3.90]
Schuh 1995	15	40	19	41	7.9%	0.81 [0.48, 1.36]
Sharma 2004	1	25	4	25	0.7%	0.25 [0.03, 2.08]
Watanasomsiri 2006	2	38	3	33	1.1%	0.58 [0.10, 3.26]
Wyatt 2015	122	174	111	173	19.5%	1.09 [0.94, 1.27]
Zorc 1999	38	211	48	216	11.3%	0.81 [0.55, 1.19]
**Total (95% CI)**		**1376**		**1378**	**100.0%**	**0.79 [0.66, 0.95]**
Total events	340		405			

Heterogeneity: $Tau^2 = 0.04$; $Chi^2 = 23.36$, df = 14 (P = 0.05); $I^2 = 40\%$
Test for overall effect: Z = 2.48 (P = 0.01)

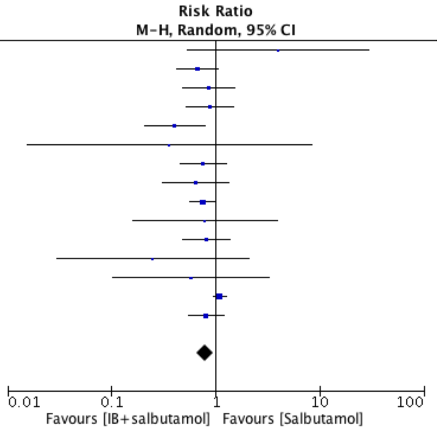

Any adverse event – with ITT dataset

Study or Subgroup	IB+salbutamol Events	IB+salbutamol Total	Salbutamol Events	Salbutamol Total	Weight	Risk Ratio M-H, Random, 95% CI
Qureshi 1997	32	45	35	45	45.3%	0.91 [0.72, 1.17]
Wyatt 2015	58	209	42	207	40.4%	1.37 [0.97, 1.94]
Yi 2015	2	42	0	42	2.4%	5.00 [0.25, 101.11]
Yin 2018	3	38	4	38	9.3%	0.75 [0.18, 3.13]
Zhang 2012	5	50	0	50	2.7%	11.00 [0.62, 193.80]
**Total (95% CI)**		**384**		**382**	**100.0%**	**1.18 [0.73, 1.90]**
Total events	100		81			

Heterogeneity: $Tau^2 = 0.12$; $Chi^2 = 9.50$, df = 4 (P = 0.05); $I^2 = 58\%$
Test for overall effect: Z = 0.66 (P = 0.51)

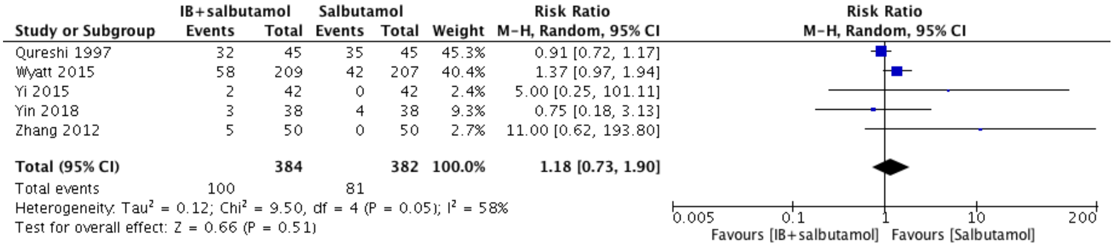

Any adverse event – without ITT dataset

Study or Subgroup	IB+salbutamol Events	IB+salbutamol Total	Salbutamol Events	Salbutamol Total	Weight	Risk Ratio M-H, Random, 95% CI
Qureshi 1997	32	45	35	45	33.1%	0.91 [0.72, 1.17]
Wyatt 2015	23	174	8	173	28.3%	2.86 [1.31, 6.21]
Yi 2015	2	42	0	42	8.7%	5.00 [0.25, 101.11]
Yin 2018	3	38	4	38	20.5%	0.75 [0.18, 3.13]
Zhang 2012	5	50	0	50	9.3%	11.00 [0.62, 193.80]
**Total (95% CI)**		**349**		**348**	**100.0%**	**1.77 [0.63, 4.98]**
Total events	65		47			

Heterogeneity: $Tau^2 = 0.82$; $Chi^2 = 17.60$, df = 4 (P = 0.001); $I^2 = 77\%$
Test for overall effect: Z = 1.09 (P = 0.28)

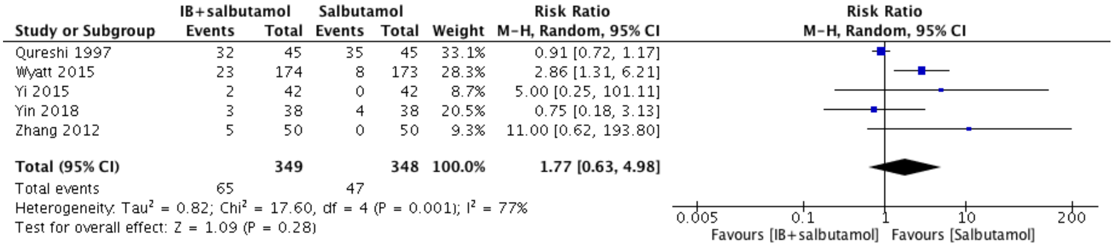

Sensitivity analysis with the assumption of risk of bias

Hospital admission – with high risk

Study or Subgroup	IB+Salbutamol Events	IB+Salbutamol Total	Salbutamol Events	Salbutamol Total	Weight	Risk Ratio M-H, Random, 95% CI
Anthracopoulos 2005	4	10	1	10	0.8%	4.00 [0.54, 29.80]
Benito Fernandez 2000	18	51	27	51	9.4%	0.67 [0.42, 1.05]
BI [pers comm]	19	246	23	254	6.8%	0.85 [0.48, 1.53]
Ducbarme 1998	23	155	24	143	7.8%	0.88 [0.52, 1.49]
Iramain 2011	9	49	22	48	5.6%	0.40 [0.21, 0.78]
Kumaratne 2003	0	24	1	26	0.3%	0.36 [0.02, 8.43]
Peterson 1996	19	82	25	81	8.1%	0.75 [0.45, 1.25]
Qureshi 1997	9	45	14	45	4.9%	0.64 [0.31, 1.33]
Qureshi 1998	59	215	80	219	14.7%	0.75 [0.57, 0.99]
Reisman 1988	2	11	3	13	1.2%	0.79 [0.16, 3.90]
Schuh 1995	15	40	19	41	7.9%	0.81 [0.48, 1.36]
Sharma 2004	1	25	4	25	0.7%	0.25 [0.03, 2.08]
Watanasomsiri 2006	2	38	3	33	1.1%	0.58 [0.10, 3.26]
Wyatt 2015	122	174	111	173	19.5%	1.09 [0.94, 1.27]
Zorc 1999	38	211	48	216	11.3%	0.81 [0.55, 1.19]
**Total (95% CI)**		**1376**		**1378**	**100.0%**	**0.79 [0.66, 0.95]**
Total events	340		405			

Heterogeneity: Tau² = 0.04; Chi² = 23.36, df = 14 (P = 0.05); I² = 40%
Test for overall effect: Z = 2.48 (P = 0.01)

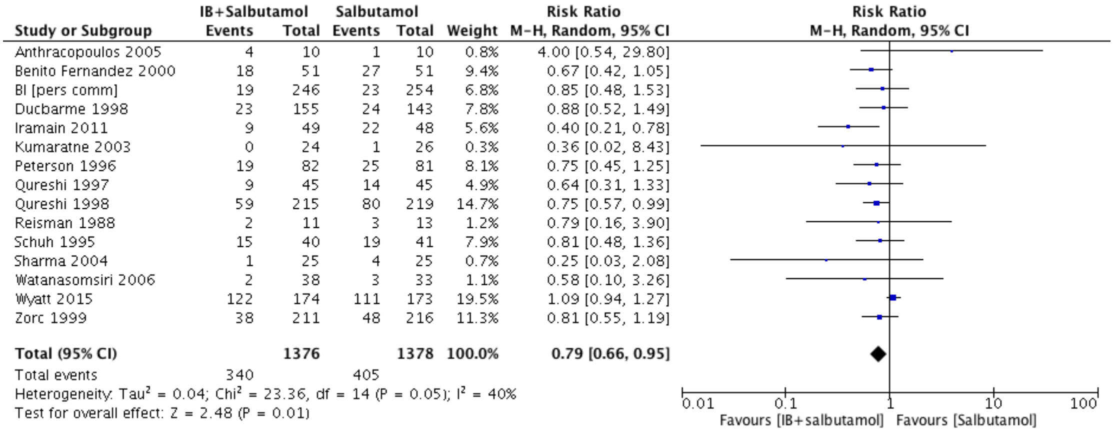

Hospital admission – without high risk

Study or Subgroup	IB+salbutamol Events	IB+salbutamol Total	Salbutamol Events	Salbutamol Total	Weight	Risk Ratio M-H, Random, 95% CI
Anthracopoulos 2005	4	10	1	10	0.8%	4.00 [0.54, 29.80]
Benito Fernandez 2000	18	51	27	51	9.4%	0.67 [0.42, 1.05]
BI [pers comm]	19	246	23	254	6.8%	0.85 [0.48, 1.53]
Ducbarme 1998	23	155	24	143	7.8%	0.88 [0.52, 1.49]
Iramain 2011	9	49	22	48	5.5%	0.40 [0.21, 0.78]
Kumaratne 2003	0	24	1	26	0.3%	0.36 [0.02, 8.43]
Peterson 1996	19	82	25	81	8.1%	0.75 [0.45, 1.25]
Qureshi 1997	9	45	14	45	4.8%	0.64 [0.31, 1.33]
Qureshi 1998	59	215	80	219	14.9%	0.75 [0.57, 0.99]
Reisman 1988	2	11	3	13	1.2%	0.79 [0.16, 3.90]
Schuh 1995	15	40	19	41	7.9%	0.81 [0.48, 1.36]
Watanasomsiri 2006	2	38	3	33	1.0%	0.58 [0.10, 3.26]
Wyatt 2015	122	174	111	173	20.1%	1.09 [0.94, 1.27]
Zorc 1999	38	211	48	216	11.4%	0.81 [0.55, 1.19]
**Total (95% CI)**		**1351**		**1353**	**100.0%**	**0.80 [0.67, 0.96]**
Total events	339		401			

Heterogeneity: Tau² = 0.04; Chi² = 21.75, df = 13 (P = 0.06); I² = 40%
Test for overall effect: Z = 2.40 (P = 0.02)

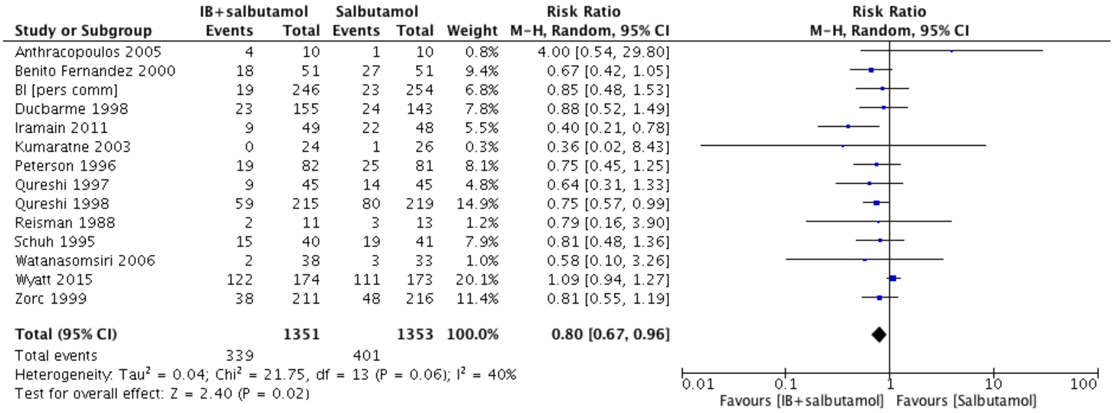

Supplement: S11 Appendix — (PDF) [file pone.0237620.s011.pdf]
