# Supplementary material for: Combination of ipratropium bromide and salbutamol in children and adolescents with asthma: A meta-analysis
Source: PLoS One. 2021 Feb 23;16(2):e0237620. doi: 10.1371/journal.pone.0237620 (PMC7901745; doi:10.1371/journal.pone.0237620)
Supplement: S12 Appendix — (PDF) [file pone.0237620.s012.pdf]

Appendix 12. Publication bias assessment

Funnel plot for primary outcome – hospital admission

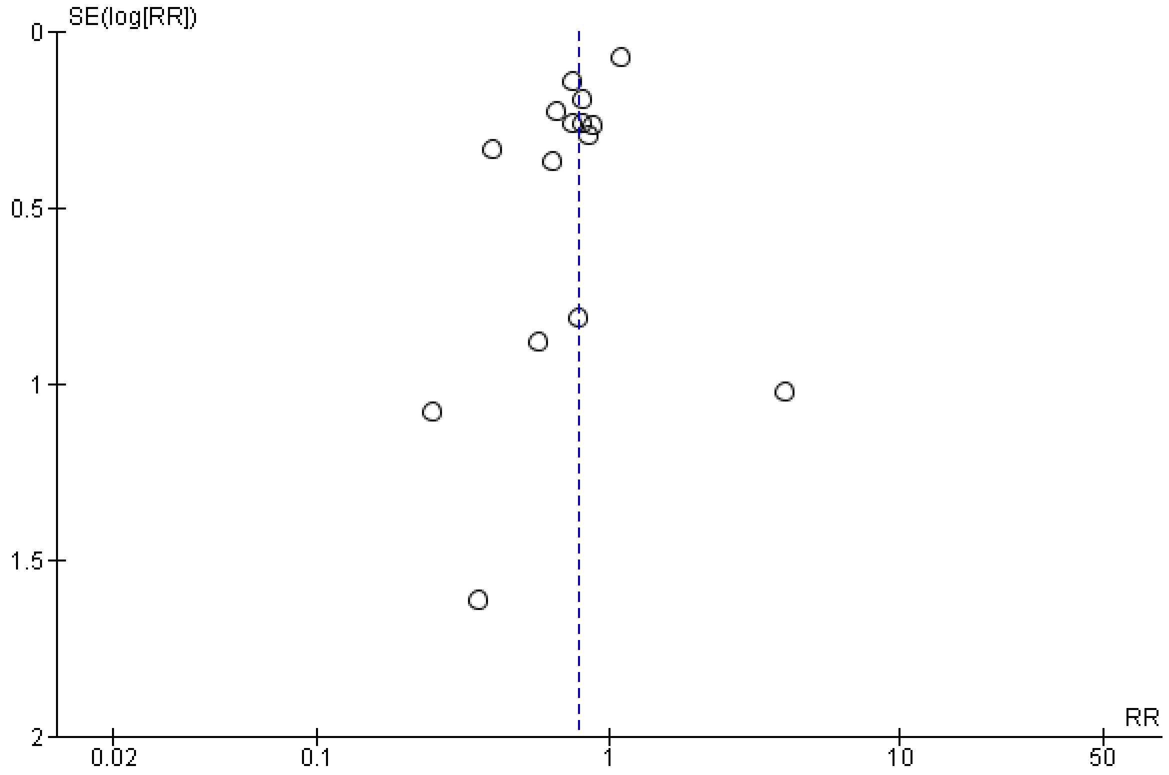

Egger's test	
Intercept	-1.024
95% CI	-1.86, -0.19
T value	-2.404
Significance level (p value)	0.03186752

Funnel plot for secondary outcome – relapse rate

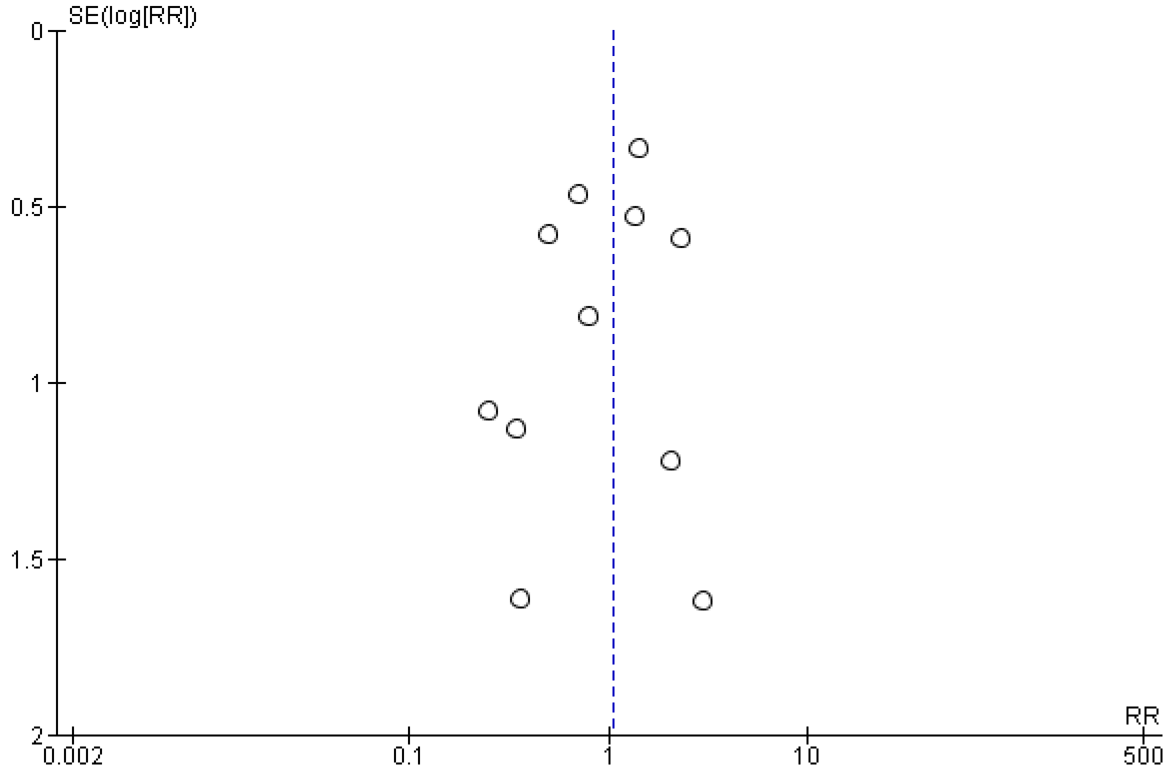

Egger's test	
Intercept	-0.505
95% CI	-1.67, -0.66
T value	-0.851
Significance level	0.416998
